# Supplementary material for: Aminopeptidase Expression in Multiple Myeloma Associates with Disease Progression and Sensitivity to Melflufen
Source: Cancers (Basel). 2021 Mar 26;13(7):1527. doi: 10.3390/cancers13071527 (PMC8036322; doi:10.3390/cancers13071527)
Supplement: Supplementary file 1 [file cancers-13-01527-s001.pdf]

Supplementary Materials

Aminopeptidase Expression in Multiple Myeloma Associates with Disease Progression and Sensitivity to Melflufen

Juho J. Miettinen, Romika Kumari, Gunnhildur Asta Traustadottir, Maiju-Emilia Huppunen, Philipp Sergeev, Muntasir M. Majumder, Alexander Schepsky, Thorarinn Gudjonsson, Juha Lievonen, Despina Bazou, Paul Dowling, Peter O`Gorman, Ana Slipicevic, Pekka Anttila, Raija Silvennoinen, Nina N. Nupponen, Fredrik Lehmann, and Caroline A. Heckman

TABLE OF CONTENTS

Supplementary figures

Figure S1. Drug plate layout and drug concentrations used in the flow cytometry-based drug sensitivity testing.

Figure S2. Flow cytometry gating strategy used in drug sensitivity testing.

Figure S3. Aminopeptidase gene family genes showed similar expression in both the CoMMpass and FIMM datasets.

Figure S4. Correlation of aminopeptidase genes *LAP3*, *ERAP2*, *METAP2*, *TPP2*, *DPP7*, *ERAP1*, *LTA4H*, *LNPEP* (Group I) expression with myeloma patient cytogenetics and age in the FIMM dataset.

Figure S5. Aminopeptidase gene expression positively correlates with aminopeptidase protein expression, especially for *LAP3*, *BLMH*, *DPP3*, *DPP7*, *RNPEP*, and *ERAP2* aminopeptidases.

Figure S6. CoMMpass dataset confirms that aminopeptidase genes are differentially expressed in NDMM vs. RRMM.

Figure S7. Correlation of aminopeptidase genes differentially expressed between RRMM and NDMM with myeloma patient cytogenetics and ISS stage in the FIMM dataset.

Figure S8. Prognostic significance of three aminopeptidase genes *XPNPEP1*, *RNPEP*, and *DPP3* expression in NDMM and RRMM samples separately in the FIMM dataset.

Figure S9. Prognostic significance of aminopeptidase gene expression in the CoMMpass dataset.

Figure S10. Somatic mutation predictions for aminopeptidase genes in the FIMM dataset samples (n = 169).

Figure S11. Somatic mutation predictions for aminopeptidase genes in CoMMpass dataset (n = 1164).

Figure S12. Aminopeptidase gene CNVs in FIMM dataset MM samples (n = 169).

Figure S13. Aminopeptidase gene CNVs in CoMMpass dataset (n = 1044).

Figure S14. *Ex vivo* sensitivity of MM CD138+CD38+ plasma cells to melflufen, melphalan, bortezomib, selinexor, and 4-HC.

Figure S15. *Ex vivo* sensitivity of MM CD138+CD38+ plasma cells to melflufen in 10 MM patients having also gene expression data available from MM CD138+ plasma cells.

Figure S16. Correlation of the 39 aminopeptidase genes expression with melflufen sensitivity in bone marrow plasma cells in 10 myeloma patient samples.

Figure S17. Correlation of drug sensitivity (EC50 values) to selinexor versus melflufen, melphalan and bortezomib in CD138+CD38+ plasma cells from MM patient samples.

Figure S18. Cytogenetics and melflufen sensitivity in CD138+CD38+ plasma cells from MM patient samples.

Figure S19. Aminopeptidase ANPEP can hydrolyze melflufen to melphalan and 4-F-Phe-OEt.

Supplementary tables

Table S1. FIMM dataset patient sample characteristics and sample analysis information.

Table S2. List of the 39 annotated aminopeptidase genes in the human genome utilizing the Ensembl and NCBI databases and further confirming the molecular function (gene ontology) of the identified genes.

Table S3. List of compounds used in the experiments.

Table S4. Aminopeptidases used in the hydrolysis assay and their incubation buffers.

Table S5. log2(RPKM) expression values for 39 aminopeptidases genes and 17 housekeeping genes in MM patient samples in the FIMM dataset (n = 122).

Table S6. LC-MS/MS-based proteomics label free quantitation intensity values for 17 aminopeptidase proteins in CD138+ cells isolated from MM patient samples in the FIMM dataset (n = 23)

Table S7. Statistical overview of genes identified as prognostic markers ($p \leq 0.05$) in the FIMM dataset (n = 122).

Table S8. Aminopeptidase gene somatic mutation frequencies from MM patient samples in the FIMM dataset (n = 169).

Table S9. Aminopeptidase gene copy number variation scores from MM patient samples in the FIMM dataset (n=169).

Table S10. Summary of live cells, and CD138+CD38+ plasma cells present in the BM-MNC samples after 72 h incubation in DMSO (control).

Table S11. Melflufen, melphalan, selinexor, bortezomib, and 4-HC EC50 values from 15 MM patient sample CD138+CD38+ plasma cells with sample disease stage indicated

Table S12. Mean log2(RPKM) values for the 39 aminopeptidase genes in melflufen high sensitivity (n = 5) and low sensitivity (n = 5) myeloma samples.

SUPPLEMENTARY FIGURES

	1	2	3	4	5	6	7	8	9	10	11	12
A	3	0.1	0.1	0.1		0.1	3	0.1	0.1	0.1		0.1
B	10	1	1	1		1	10	1	1	1		1
C	30	10	10	10		3	30	10	10	10		3
D	DMSO	DMSO	BzCl	DMSO	DMSO	DMSO	DMSO	DMSO	BzCl	DMSO	DMSO	DMSO
E	100	100	100	100		10	100	100	100	100		10
F	300	1000	300	1000		30	300	1000	300	1000		30
G	1000	10000	1000	10000		100	1000	10000	1000	10000		100
H	3000	100000	10000	100000		1000	3000	100000	10000	100000		1000
	Selinexor (1/2)	Melflufen (1/2)	4-HC (1/2)	Melphalan (1/2)		Bortezomib (1/2)	Selinexor (2/2)	Melflufen (2/2)	4-HC (2/2)	Melphalan (2/2)		Bortezomib (2/2)

Supplementary Figure S1. Drug plate layout and drug concentrations used in the flow cytometry-based drug sensitivity testing. The drug plate (96-wells) contains five different drugs (selinexor, melflufen, 4-HC, melphalan, bortezomib) at seven different concentrations (nM concentrations are shown), lighter blue indicating lower concentrations and darker blue higher concentrations. Duplicate columns for each drug are indicated on the plate as (1/2) & (2/2). The plate contains 10 negative control wells (0.2% DMSO) marked in green and 2 positive control wells (10 000 nM BzCl) marked in red.
DMSO: dimethyl sulfoxide; BzCl: benzoyl chloride: 4-HC: 4-hydroperoxycyclophosphamide

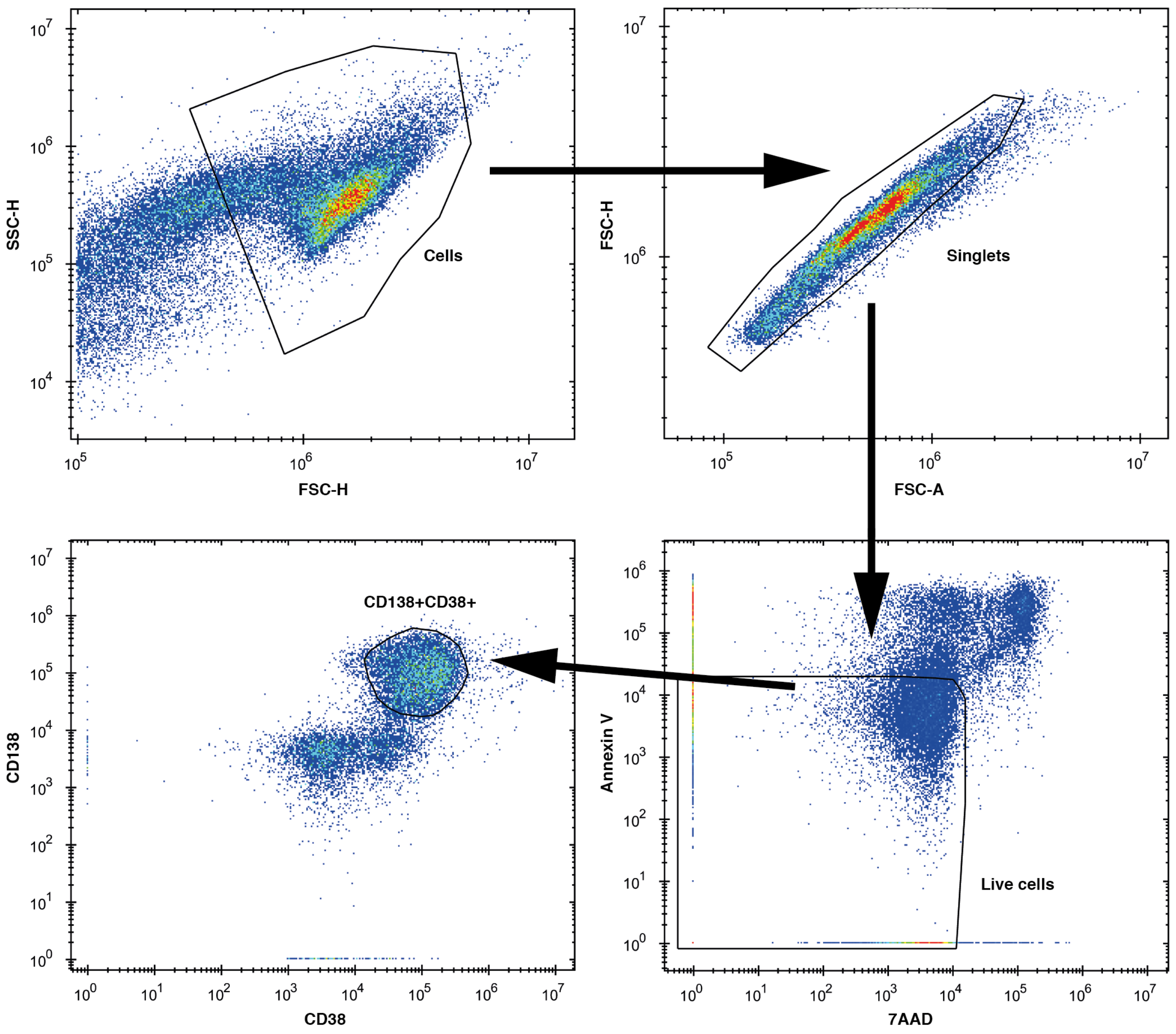

Supplementary Figure S2. Flow cytometry gating strategy used in drug sensitivity testing. Cells are first gated from all detected events. Singlet cells are gated from all cells. Live cells (Annexin V−, 7AAD−) are gated from singlet cells. CD138+CD38+ plasma cells are gated from all live cells. Negative control DMSO well from sample MM128_1 was used as an example for the gating strategy. 7-AAD: 7-amino-actinomycin D; DMSO: dimethyl sulfoxide; FSC-H: forward scatter height; FSC-A: forward scatter area; SSC-H: side scatter height.

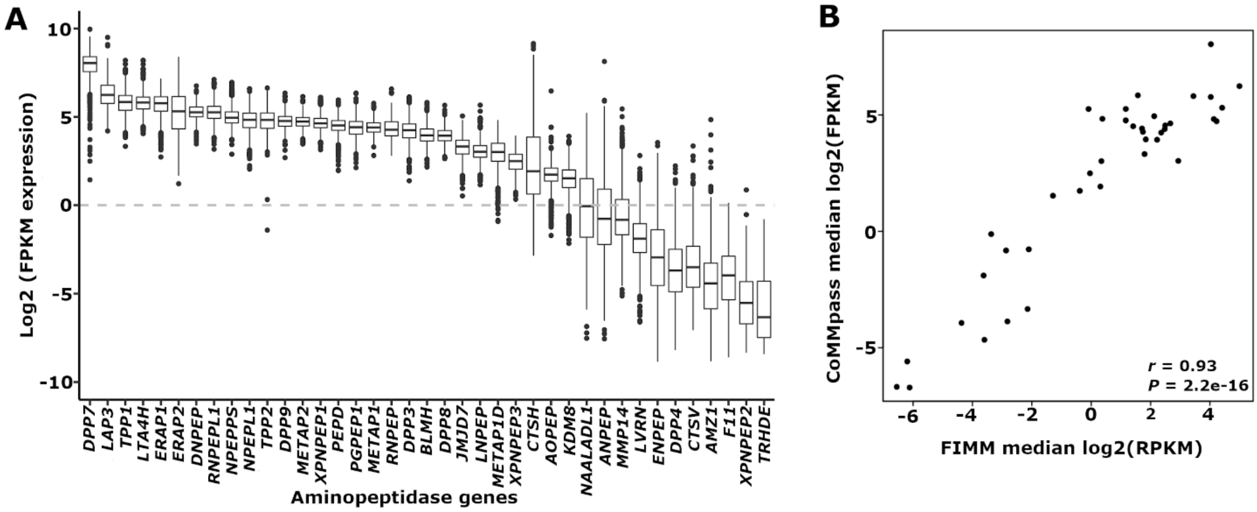

Supplementary Figure S3. Aminopeptidase gene family genes showed similar expression in both the CoMMpass and FIMM datasets. **(A)** Boxplots showing the expression of aminopeptidase genes in CoMMpass dataset (*n* = 892). **(B)** Correlation of median aminopeptidase gene expression between the FIMM dataset and the CoMMpass dataset. Each dot represents a gene (*n* = 39) (r = 0.93, *p* = 2.2 x 10^{-16}).

FPKM: fragments per kilobase of transcript per million mapped reads; RPKM: reads per kilobase of transcript per million mapped reads

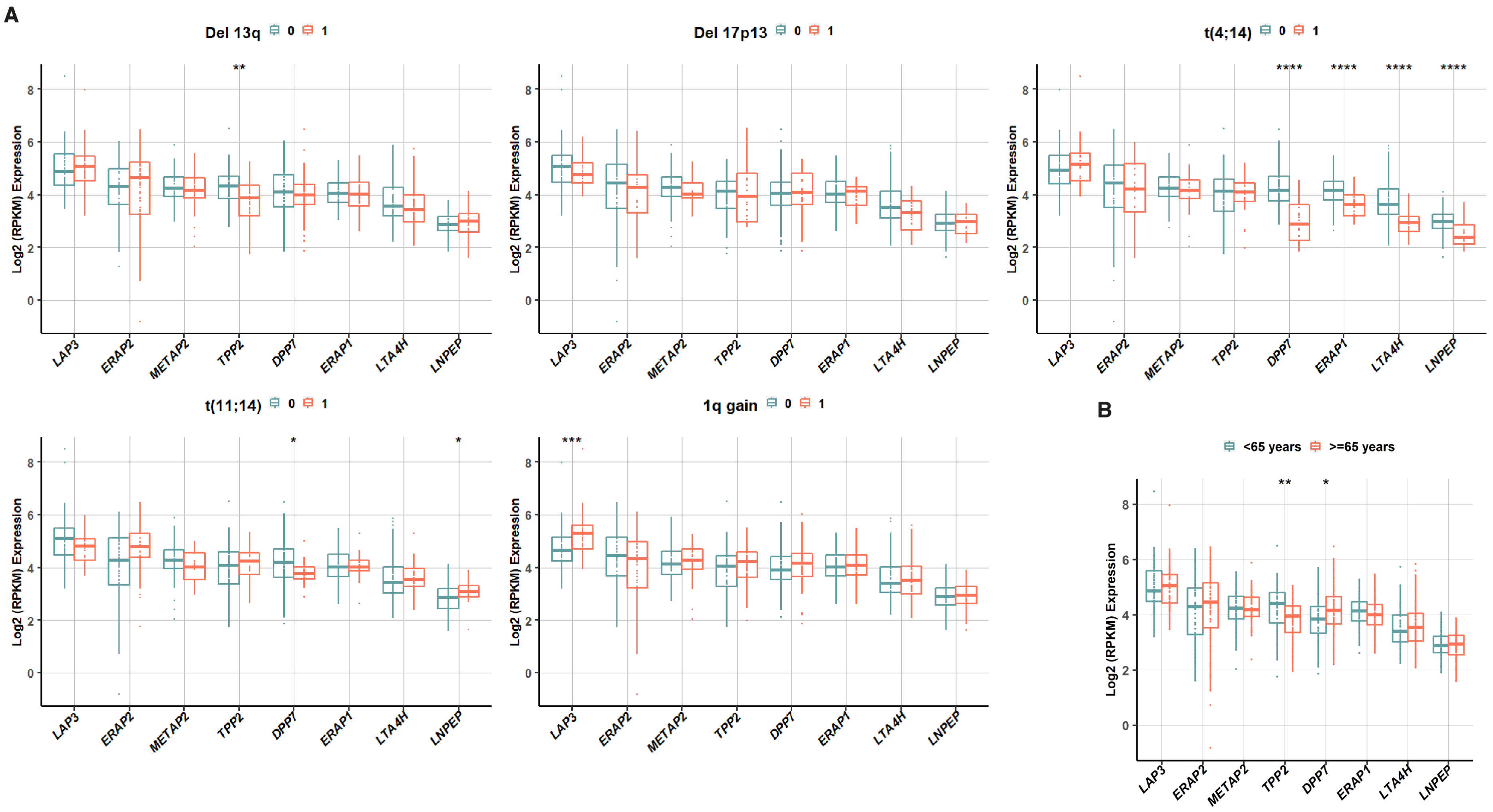

Supplementary Figure S4. Correlation of aminopeptidase genes *LAP3*, *ERAP2*, *METAP2*, *TPP2*, *DPP7*, *ERAP1*, *LTA4H*, *LNPEP* (Group I) expression with myeloma patient cytogenetics and age in the FIMM dataset. **(A)** Correlation of aminopeptidase gene expression with cyto-genetics (Del 13q, Del 17p, t(4;14), t(11;14), 1q gain). **(B)** Correlation of aminopeptidase gene expression with patient age (<65 years of age, ≥65 years of age). Statistical significance is indicated as *adjusted P≤ 0.1; **adjusted P≤ 0.05; ***adjusted P≤ 0.01; ****adjusted P≤ 0.001.
0: patient doesn't have the cytogenetic abnormality (blue box plot); 1: patient has the cytogenetic abnormality (red box plot); RPKM: Reads Per Kilobase of transcript per Million mapped reads

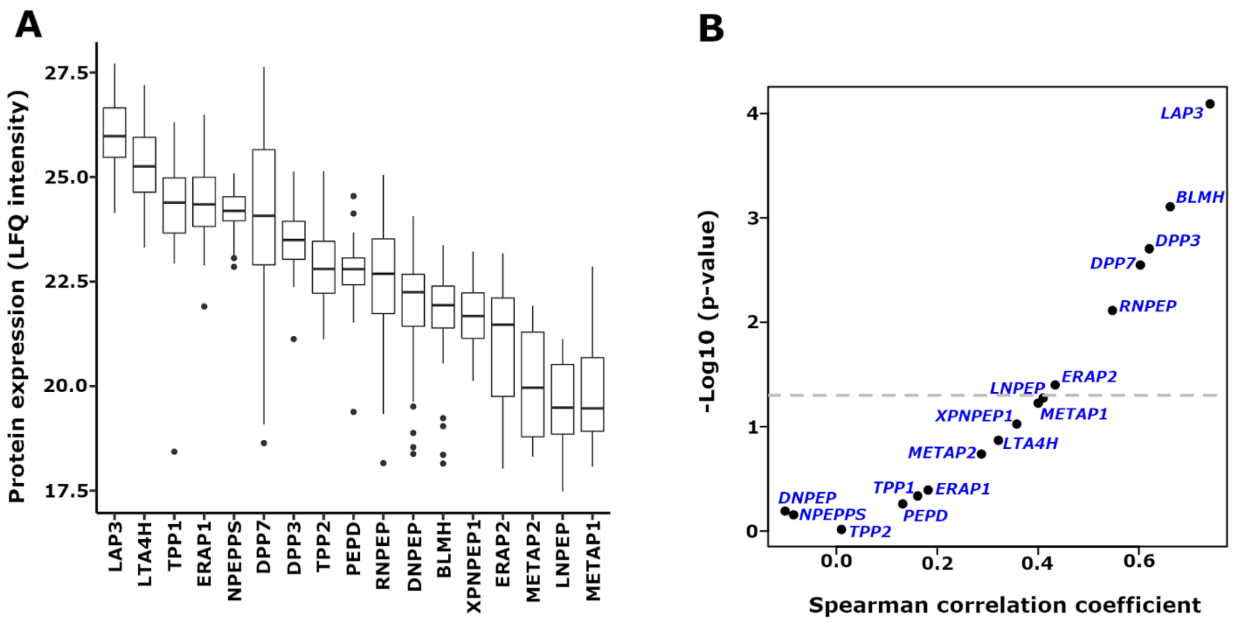

Supplementary Figure S5. Aminopeptidase gene expression positively correlates with aminopeptidase protein expression, especially for LAP3, BLMH, DPP3, DPP7, RNPEP, and ERAP2 aminopeptidases. (A) Aminopeptidase protein expression levels were measured using LC-MS/MS-based label-free quantitative proteomics from CD138+ cells enriched from 23 MM patient bone marrow aspirates. Peptides from 17 aminopeptidase proteins were detected by LC-MS/MS-based proteomics. Protein expression levels are indicated as LFQ intensity values. **(B)** Correlation of protein expression and gene expression levels for the 17 aminopeptidases.

LC-MS/MS: liquid chromatography-tandem mass spectrometry; LFQ: label-free quantification; MM: multiple myeloma.

A

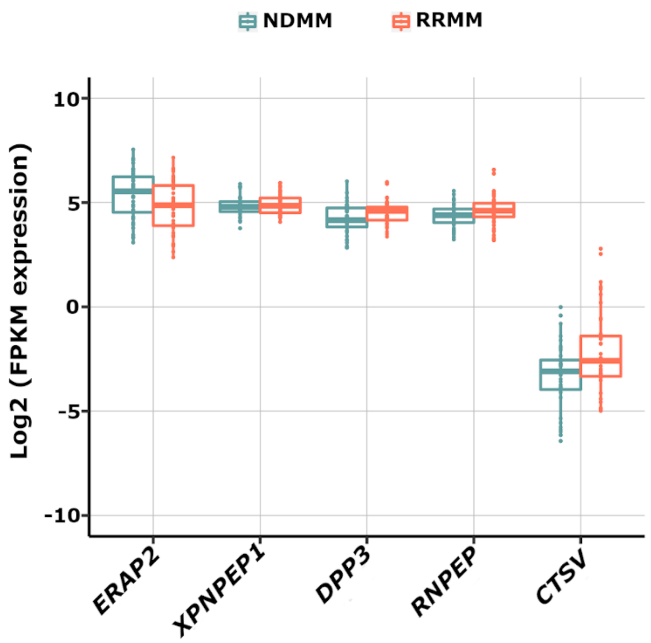

B

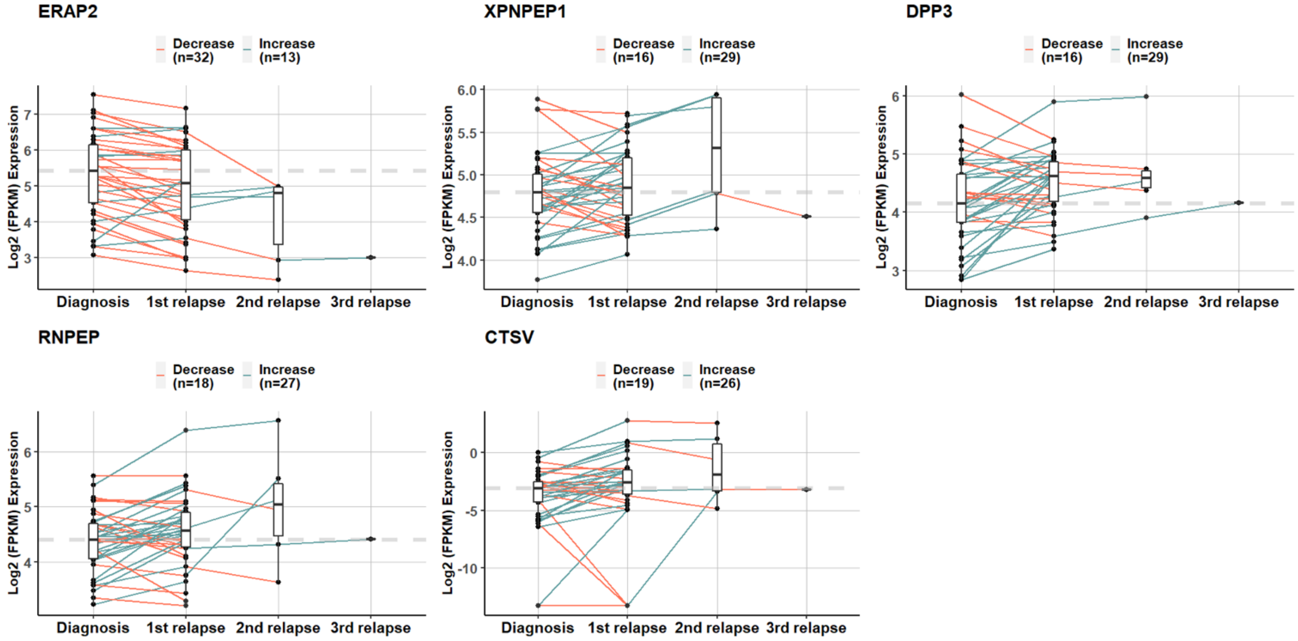

Supplementary Figure S6. CoMMpass dataset confirms that aminopeptidase genes are differentially expressed in NDMM vs. RRMM. The CoMMpass dataset differential gene expression analysis in NDMM *versus* RRMM samples was performed using paired samples (NDMM = 39, RRMM = 45). **(A)** The CoMMpass dataset differential gene expression analysis for the 5 aminopeptidase genes (*ERAP2*, *XPNPEP1*, *DPP3*, *RNPEP*, *CTSV*) confirming that they are differentially expressed in NDMM *versus* RRMM. **(B)** Description of the sequential changes in the gene expression of the 5 aminopeptidase genes (*ERAP2*, *XPNPEP1*, *DPP3*, *RNPEP*, *CTSV*) in the 39 myeloma patients in the CoMMpass dataset having paired samples. The relapse number indicated on the x-axis can be later than indicated. The x-axis provides gene expression data from RRMM in the order in which gene expression data is available from the patient.

NDMM: newly diagnosed multiple myeloma; RRMM: relapsed/refractory multiple myeloma; FPKM: fragments per kilobase of transcript per million mapped reads

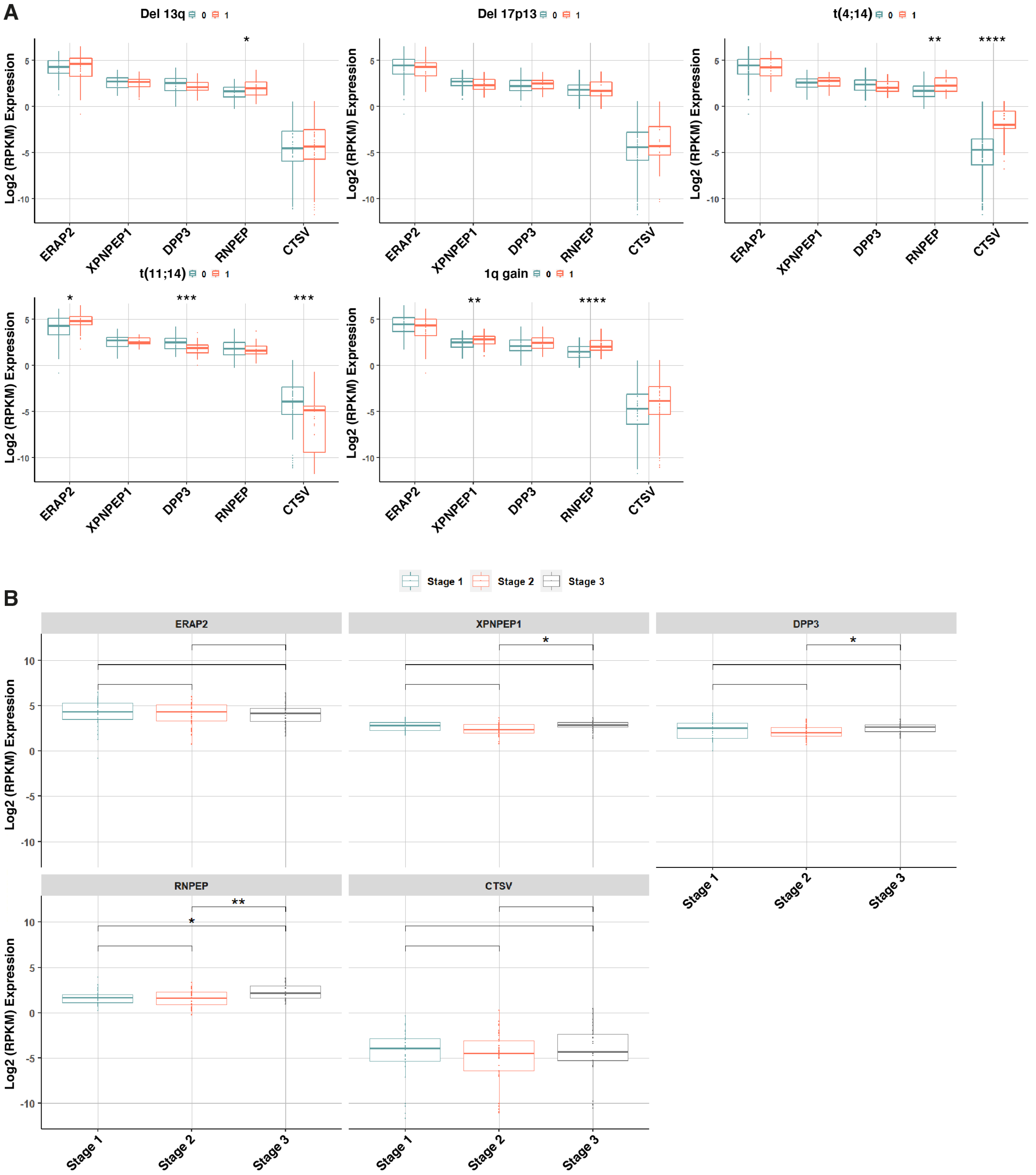

Supplementary Figure S7. Correlation of aminopeptidase genes differentially expressed between RRMM and NDMM with myeloma patient cytogenetics and ISS stage in the FIMM dataset. **(A)** Correlation of aminopeptidase gene (*ERAP2*, *XPNPEP1*, *DPP3*, *RNPEP*, *CTSV*) expression with cytogenetics (del 13q, del 17p, t(4;14), t(11;14), 1q gain). **(B)** Correlation of aminopeptidase gene expression (ERAP2, XPNPEP1, DPP3, RNPEP, CTSV) with International Staging System (ISS) stage. Statistical significance is indicated as *adjusted $p \leq 0.1$; **adjusted $p \leq 0.05$; ***adjusted $p \leq 0.01$; ****adjusted $p \leq 0.001$.

0: patient doesn't have the cytogenetic abnormality; 1: patient has the cytogenetic abnormality; RPKM: reads per kilobase of transcript per million mapped reads

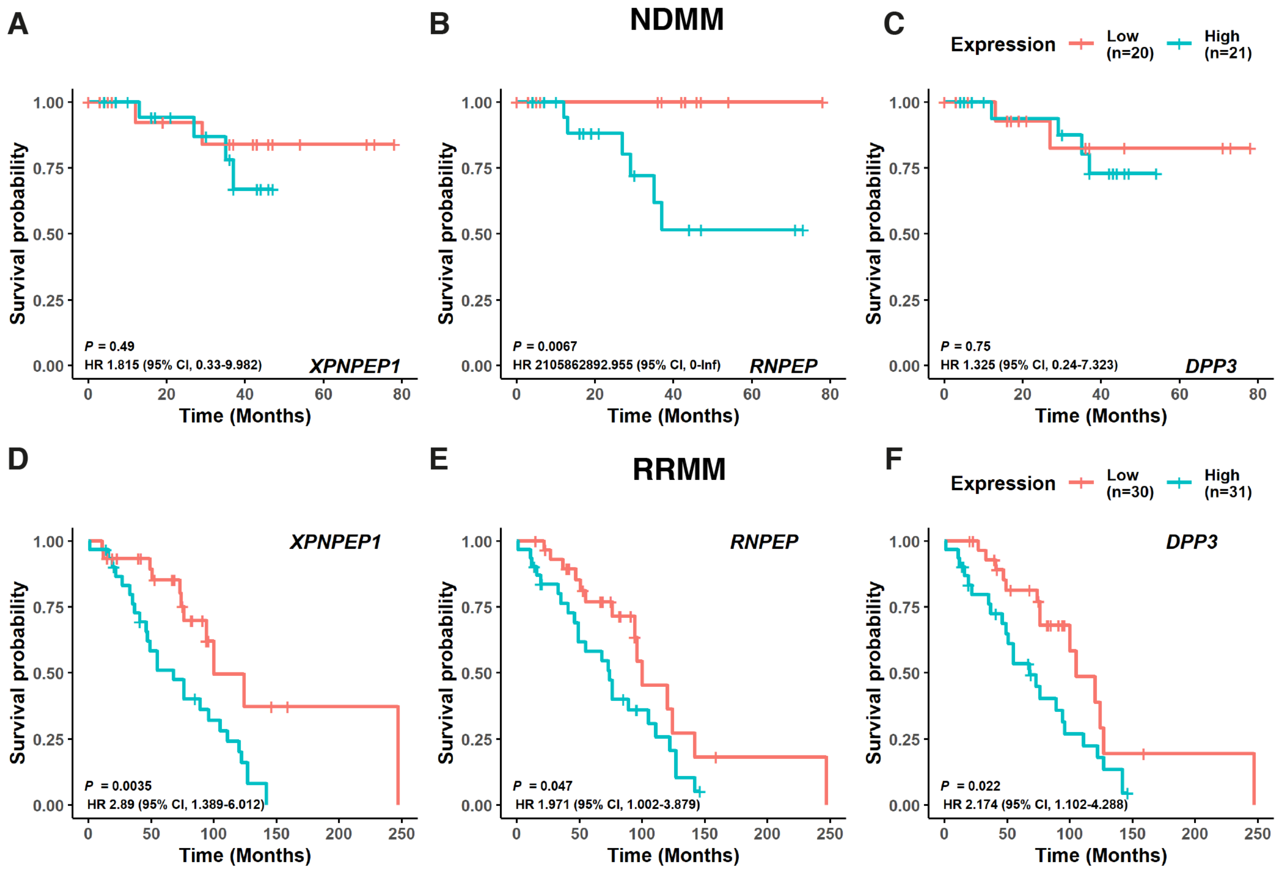

Supplementary Figure S8. Prognostic significance of three aminopeptidase genes *XPNPEP1*, *RNPEP,* and *DPP3* expression in NDMM and RRMM samples separately in the FIMM dataset. Analysis was performed using data from 41 NDMM and 61 RRMM patient samples the FIMM dataset. RRMM samples were selected on the basis of gene expression data availability from the first possible relapse sample available. **(A-C)** Survival curves drawn based on the FIMM dataset including only NDMM for the 3 aminopeptidase genes *XPNPEP1*, *RNPEP* and *DPP3* associated with poor prognosis in both the FIMM and CoMMpass datasets when both NDMM and RRMM samples were pooled together: **(A)** *XPNPEP1*, **(B)** *RNPEP* and **(C)** *DPP3*. **(D-F)** Survival curves drawn based on the FIMM dataset including only RRMM for the 3 aminopeptidase genes *XPNPEP1*, *RNPEP* and *DPP3* associated with poor prognosis ($p \leq 0.05$) in both the FIMM and CoMMpass datasets when both NDMM and RRMM samples were pooled together: **(D)** *XPNPEP1*, **(E)** *RNPEP* and **(F)** *DPP3*. HR: hazard ratio; CI: confidence interval

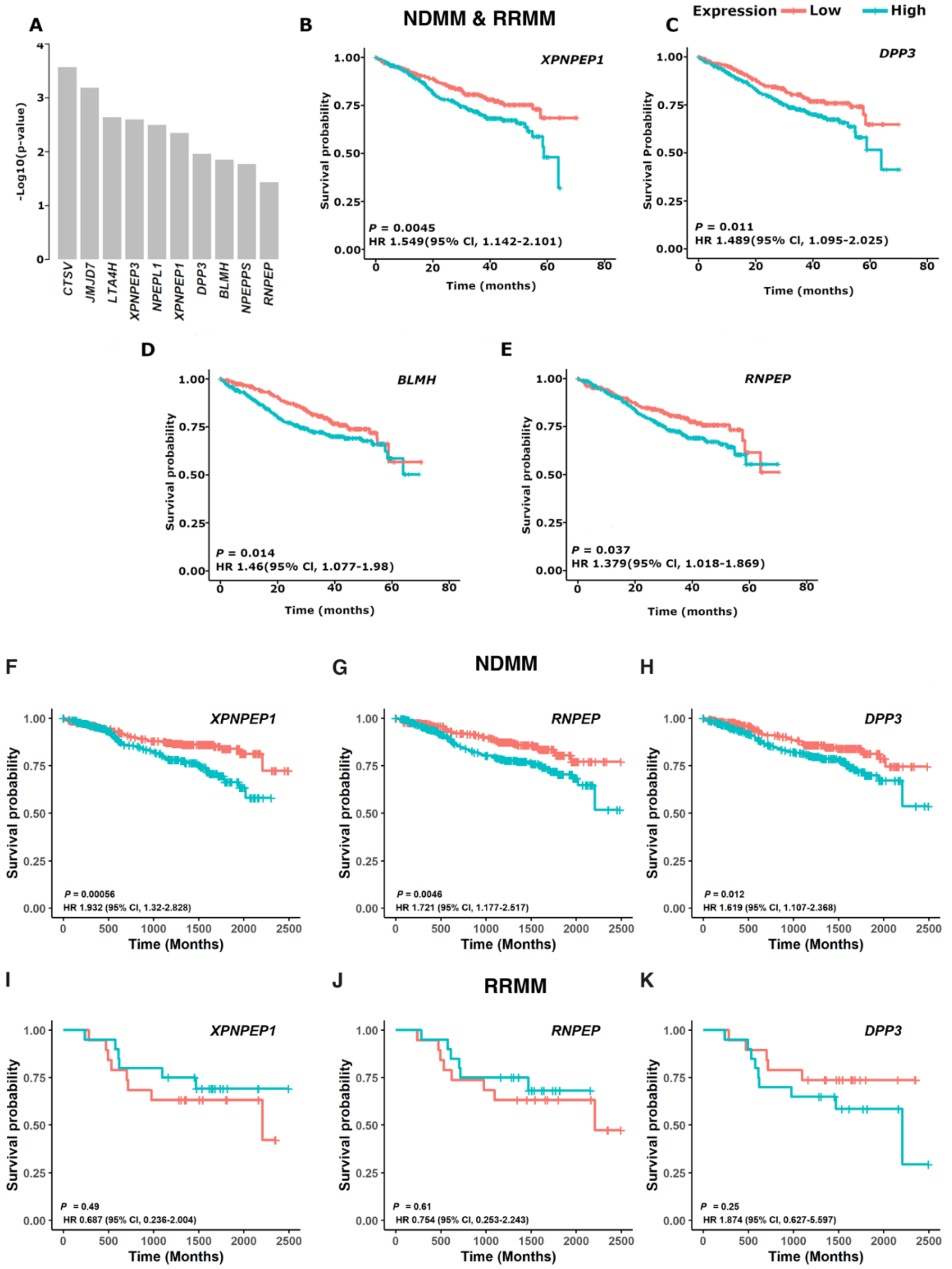

Supplementary Figure S9. Prognostic significance of aminopeptidase gene expression in the CoMMpass dataset. Analysis was performed using data from 768 NDMM patient samples in the CoMMpass dataset. **(A)** *p*-value distribution of the aminopeptidase genes associated with poor prognosis ($p < 0.05$) in the CoMMpass dataset. **(B-E)** Survival curves drawn based on the CoMMpass dataset including both NDMM and RRMM samples for the 4 aminopeptidase genes associated with poor prognosis ($p \leq 0.05$) in both the FIMM and CoMMpass datasets: **(B)** *XPNPEP1*, **(C)** *DPP3,* **(D)** *BLMH* and **(E)** *RNPEP*. **(F-H)** Survival curves drawn based on the CoMMpass dataset including only NDMM for the 3 aminopeptidase genes *XPNPEP1*, *RNPEP* and *DPP3* associated with poor prognosis ($p \leq 0.05$) in both the FIMM and CoMMpass datasets when both NDMM and RRMM samples were pooled together: **(F)** *XPNPEP1*, **(G)** *RNPEP* and **(H)** *DPP3*. **(I-K)** Survival curves drawn based on the CoMMpass dataset including only RRMM for the 3 aminopeptidase genes *XPNPEP1*, *RNPEP* and *DPP3* associated with poor prognosis in both the FIMM and CoMMpass datasets: **(I)** *XPNPEP1*, **(J)** *RNPEP* and **(K)** *DPP3*.
HR: hazard ratio; CI: confidence interval

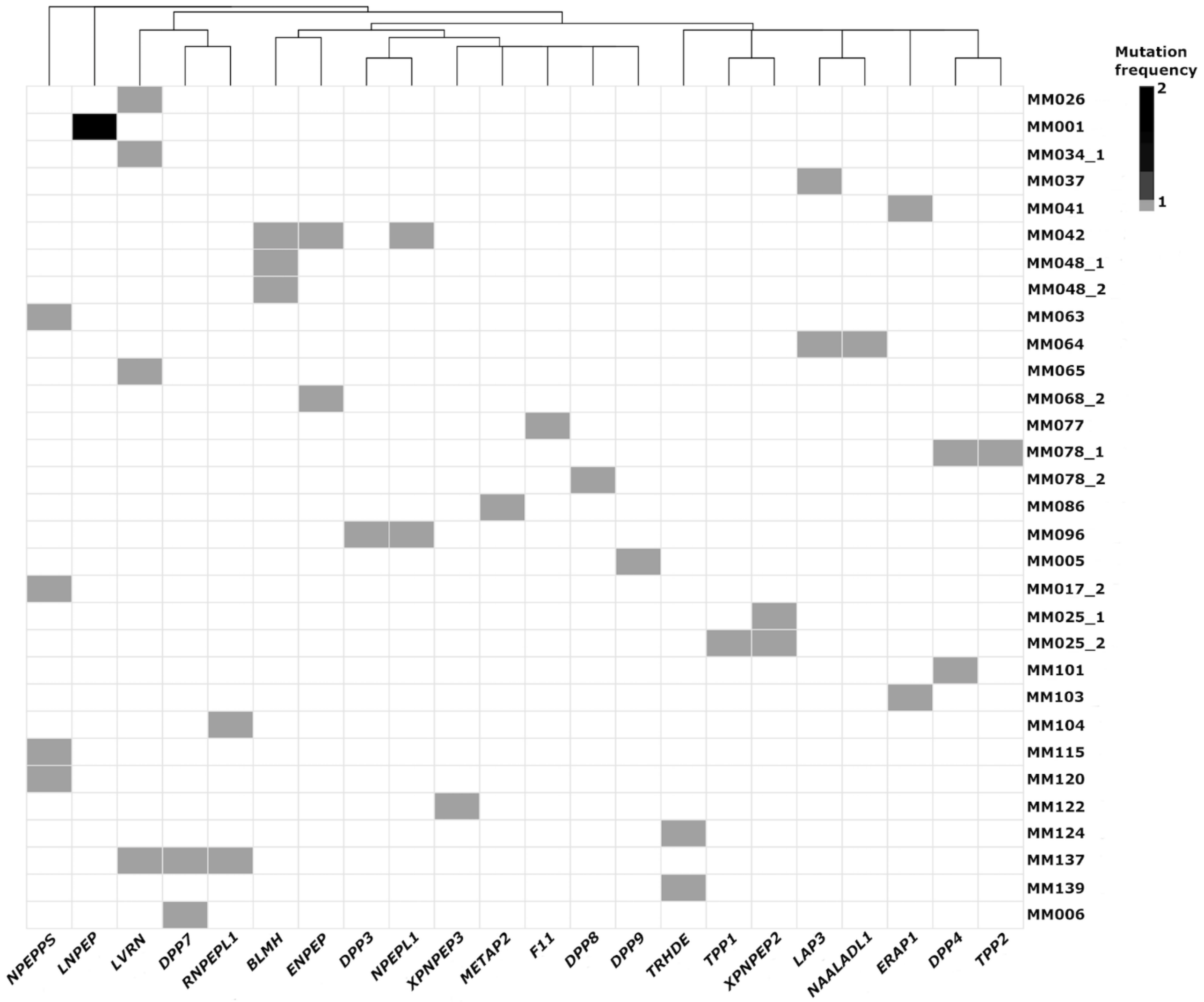

Supplementary Figure S10. Somatic mutation predictions for aminopeptidase genes in the FIMM dataset samples (*n* = 169). Somatic mutations are rare in this gene family with all genes having < 2.3% predicted mutations. Samples (*n* = 31) having one or more predicted somatic mutations in the 39 aminopeptidase genes are shown in the figure. Only those aminopeptidase genes (*n* = 22) predicted to have somatic mutations in the dataset are shown.

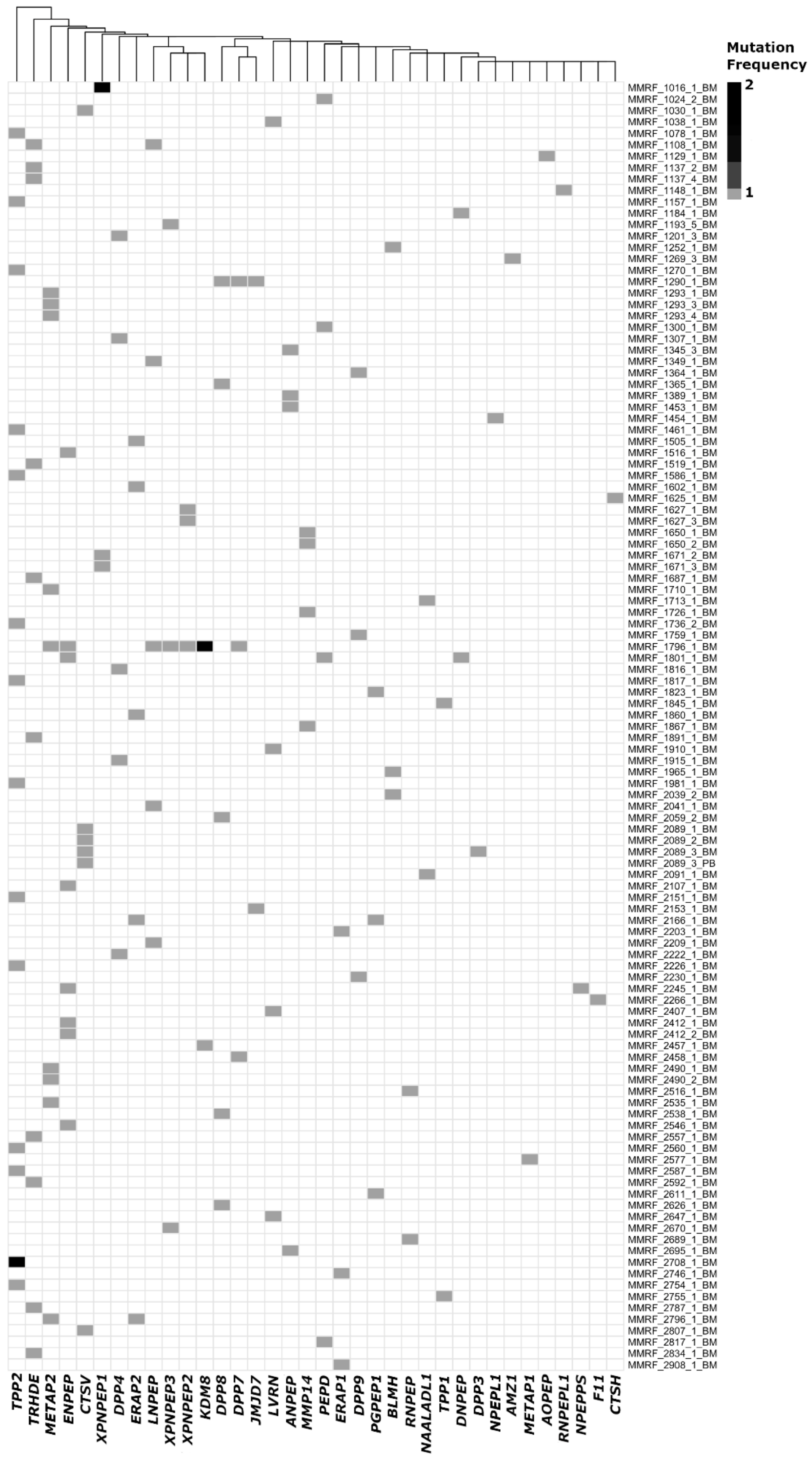

Supplementary Figure S11. Somatic mutation predictions for aminopeptidase genes in CoMMpass dataset (*n* = 1164). Somatic mutations are rare in this gene family with all genes having < 1.2% predicted mutations. Samples (*n* = 113) having one or more predicted somatic mutations in the 39 aminopeptidase genes are shown in the figure. Only those aminopeptidase genes (*n* = 36) predicted to have somatic mutations in the dataset are shown.

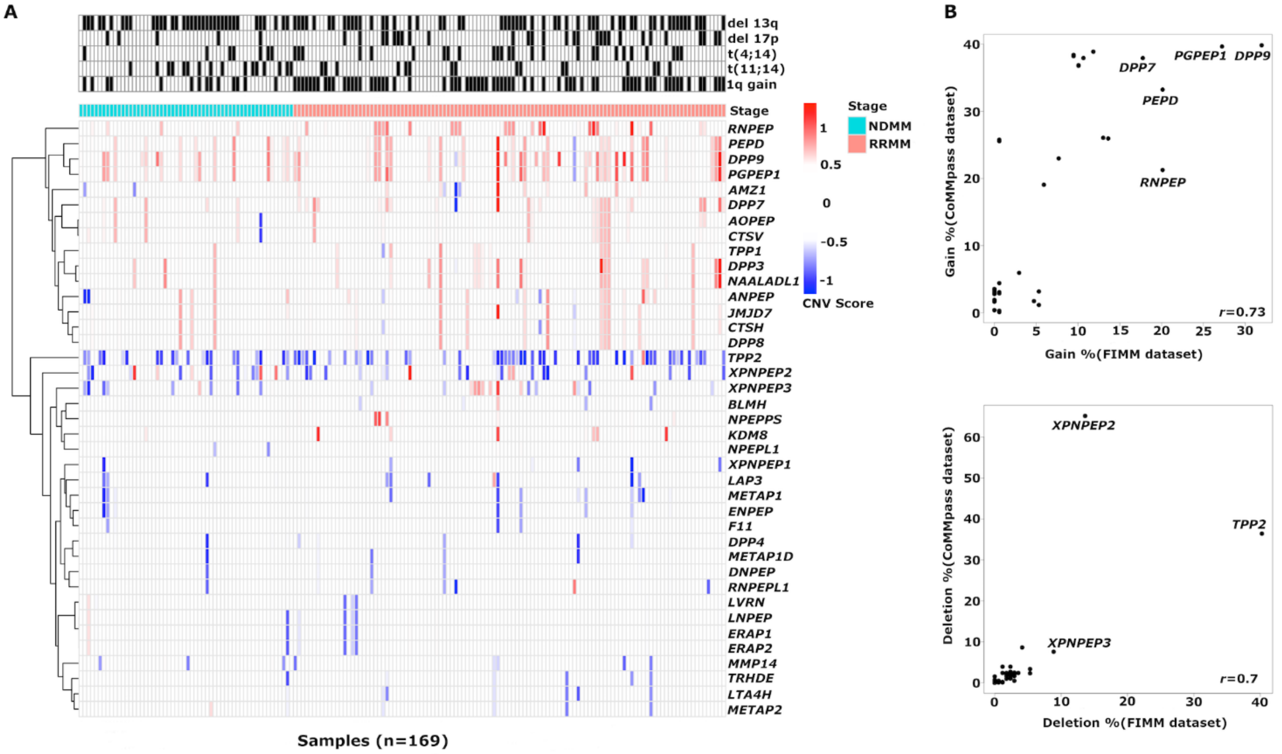

Supplementary Figure S12. Aminopeptidase gene CNVs in FIMM dataset MM samples (*n* = 169). (A) Heatmap showing the copy number variations in 39 aminopeptidase genes from the FIMM dataset of 169 MM samples (NDMM (*n* = 56), RRMM (*n* = 113)). Genes *DPP9, PGPEP1, RNPEP, PEPD* and *DPP7* were found to have a gain in > 15% of the samples, and genes *TPP2* and *XPNPEP2* were found to have a deletion in > 10% of samples. Sample cytogenetics (del 13q, del 17p, t(4;14), t(11;14), 1q gain) are indicated above the heatmap. Disease stage is also indicated (NDMM in cyan, with samples on the left; RRMM in pink, with samples on the right). **(B)** Aminopeptidase gene CNV results were further validated in CoMMpass dataset, where % gain in FIMM and CoMMpass dataset had correlation coefficient of 0.75 and % deletion had 0.69 correlation coefficient.
MM: multiple myeloma; NDMM: newly diagnosed multiple myeloma; RRMM: relapsed/refractory multiple myeloma; CNV: copy number variation

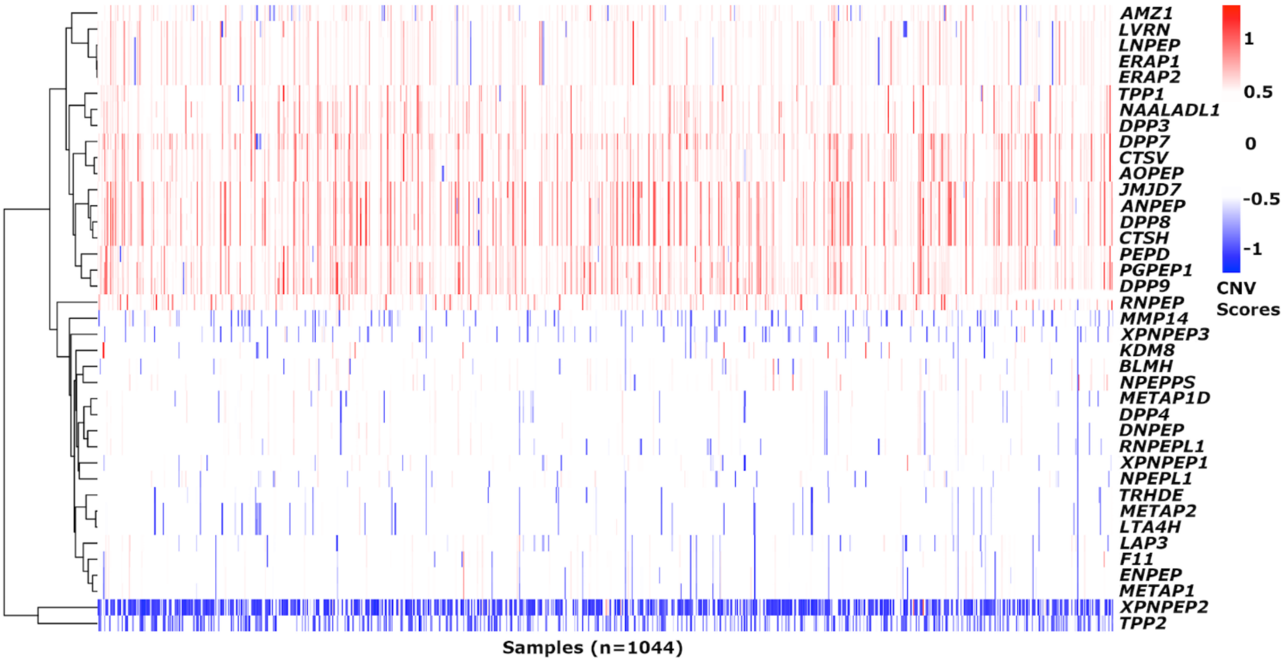

Supplementary Figure S13. Aminopeptidase gene CNVs in CoMMpass dataset (*n* = 1044).
CNV: copy number variation

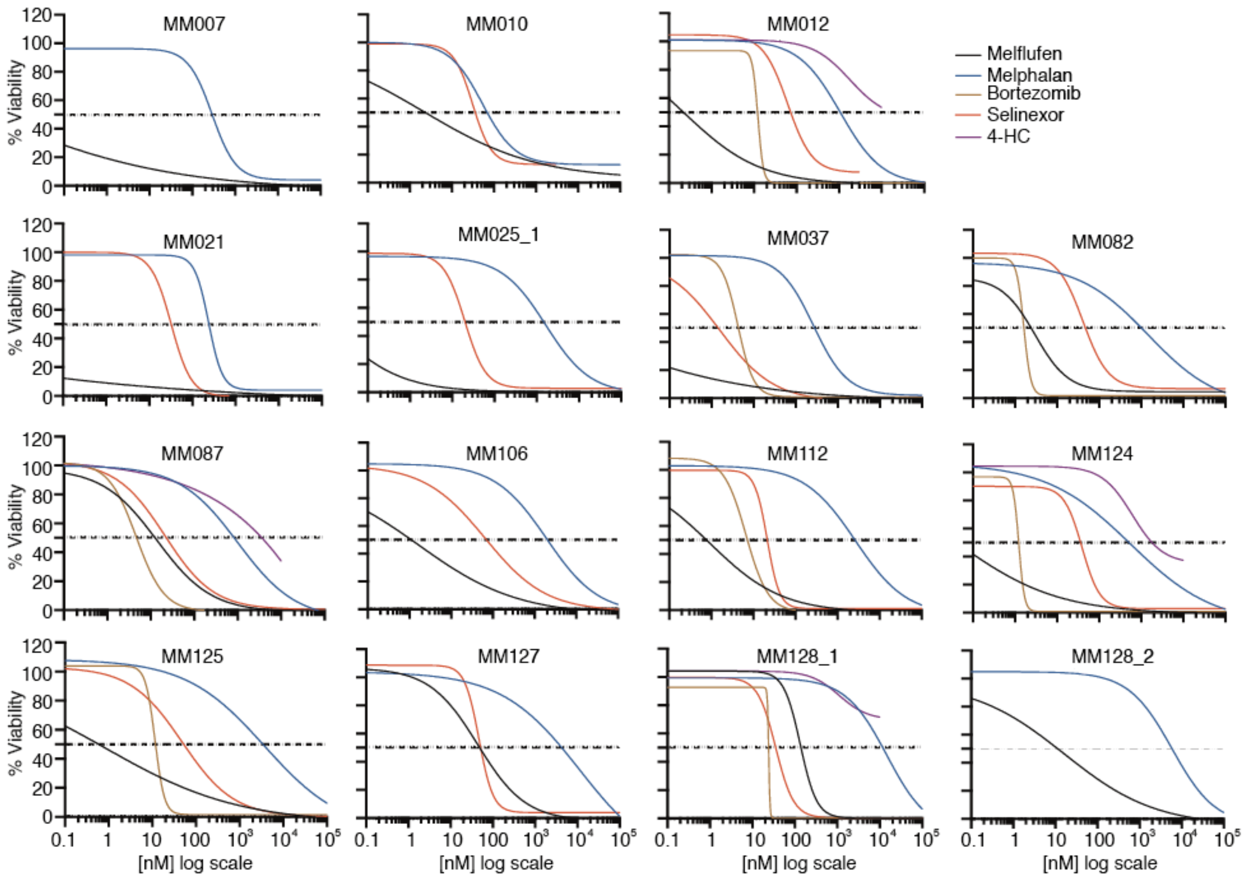

Supplementary Figure S14. ***Ex vivo*** **sensitivity of MM CD138+CD38+ plasma cells to melflufen, melphalan, bortezomib, selinexor, and 4-HC.** Patient samples were treated with drugs (melflufen, melphalan, bortezomib, selinexor, 4-HC), or with DMSO (control) for 72 h. After treatment cell viability was measured by multicolor high throughput flow cytometry, with cell viability (%) calculated and dose-response curves drawn. Drug sensitivity was measured in CD138+CD38+ cells from 15 MM patient samples (NDMM n = 6; RRMM n = 9) with drug dose-response curves shown for each sample: melflufen (black; n = 15), melphalan (blue; n = 15), bortezomib (brown; n = 8), selinexor (red; n = 13), and 4-HC (magenta; n = 4). Some of the samples have only a limited number of drugs tested. This is due to there not being enough viable cells at the time of cell plating in these samples to perform drug testing for all 5 drugs.

DMSO, dimethyl sulfoxide; MM: multiple myeloma; NDMM: newly diagnosed multiple myeloma; RRMM: relapsed/refractory multiple myeloma; 4-HC: 4-hydroperoxycyclophosphamide

A

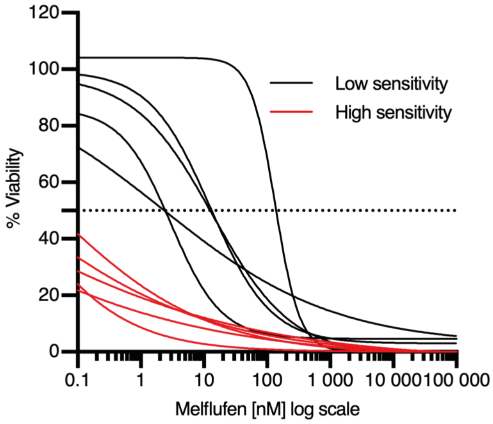

B

Sample ID	Melflufen EC50 [nM]	Melflufen sensitivity group
MM037	**0.000285**	**High sensitivity**
MM007	**0.001414**	**High sensitivity**
*MM135	**0.009226**	**High sensitivity**
MM025_1	**0.01096**	**High sensitivity**
MM124	**0.04291**	**High sensitivity**
MM010	2.185	Low sensitivity
MM082	2.992	Low sensitivity
*MM117_2	12.56	Low sensitivity
MM087	13.81	Low sensitivity
MM128_1	134.5	Low sensitivity

Supplementary Figure S15. *Ex vivo* sensitivity of MM CD138+CD38+ plasma cells to melflufen in 10 MM patients with gene expression data also available from MM CD138+ plasma cells. (A) Drug sensitivity was measured after 72h incubation with melflufen or with DMSO alone (control). Cell viability was assessed by multicolor high throughput flow cytometry using annexin V and 7AAD viability markers. The viability (%) of CD138+CD38+ cells was calculated, and dose response curves drawn. The MM patient samples were divided into two groups based on the sensitivity of their CD138+CD38+ cells to melflufen: high sensitivity (red: EC50 < 0.1 nM) and low sensitivity (black: EC50 > 2 nM) having five samples in each group. **(B)** EC50 values and melflufen sensitivity group are indicated for each of the ten samples.

DMSO: dimethyl sulfoxide; *: myeloma samples having *ex vivo* drug sensitivity data available only from melflufen.

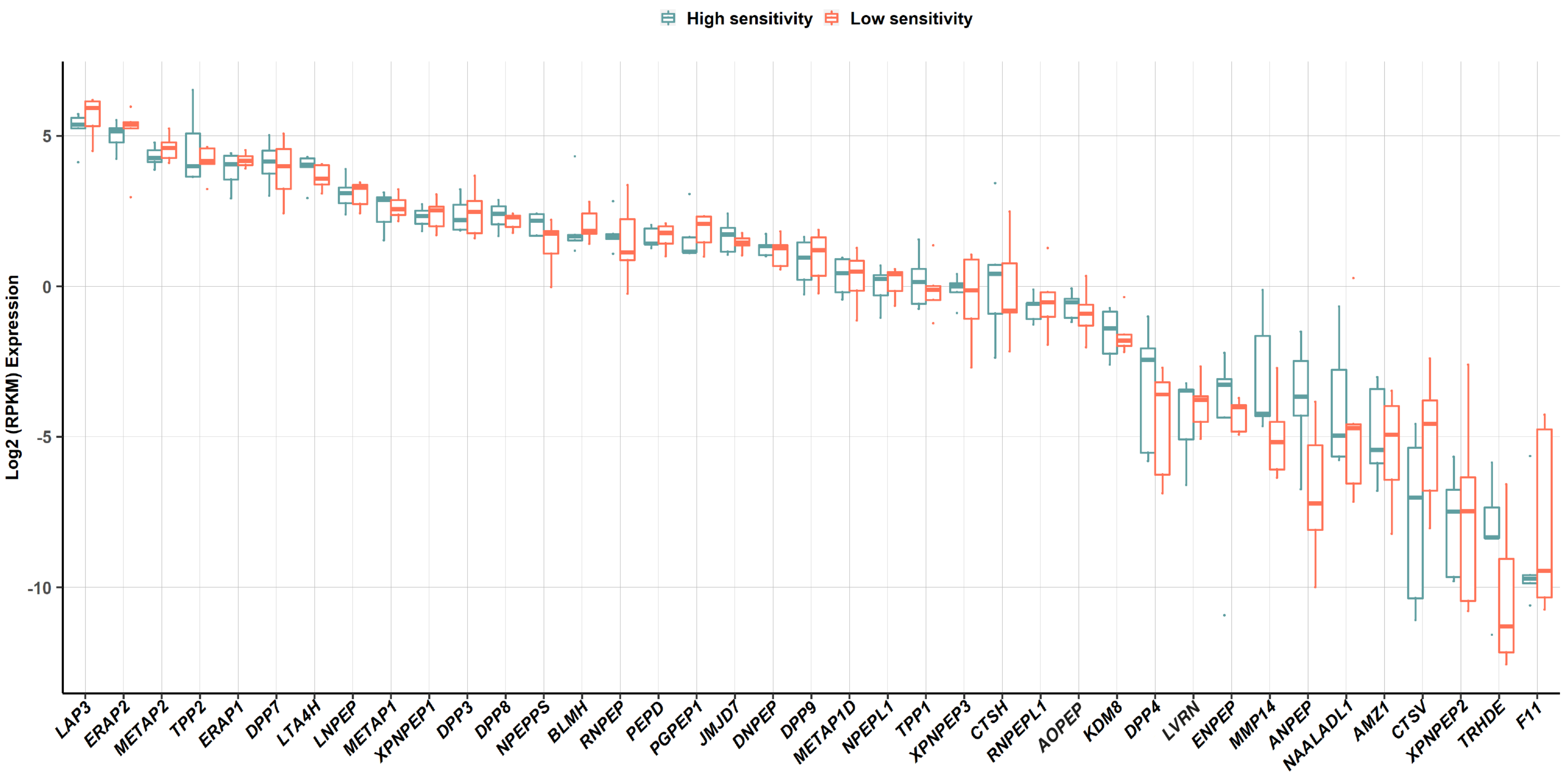

Supplementary Figure S16. Correlation of the 39 aminopeptidase genes expression with melflufen high sensitivity (n=5) and low sensitivity (n=5) sample groups. Bone marrow CD138+ cells were enriched from 10 myeloma patient samples and gene expression analysis performed. Bone marrow mononuclear cells from the same 10 samples flow cytometry-based ex vivo drug sensitivity testing was performed for CD138+CD38+ cells to determine sample sensitivity to melflufen. Ten myeloma patient samples having both aminopeptidase gene expression data (Log2 RPKM) and ex vivo melflufen drug sensitivity data (EC50) were divided into two groups based on their sensitivity to melflufen. EC50 values for the five high sensitivity samples (blue box plots; EC50 < 0.1 nM) and for the five low sensitivity samples (red box plots; EC50 > 2 nM). The mean gene expression levels (Log2 RPKM) of the 39 aminoepeptidase genes were compared between the high sensitivity and low sensi-tivity sample groups.
RPKM: Reads Per Kilobase of transcript per Million mapped reads

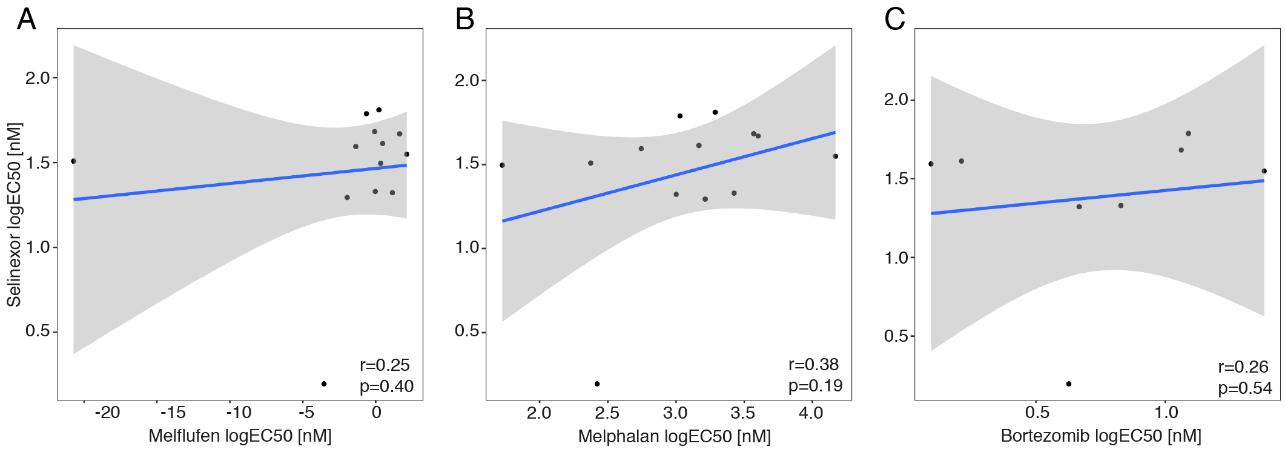

Supplementary Figure S17. Correlation of drug sensitivity (EC50 values) to selinexor versus melflufen, melphalan and bortezomib in CD138+CD38+ plasma cells from MM patient samples. EC50 values were calculated from MM patient samples using multicolor high throughput flow cytometry-based drug sensitivity testing. Correlation of selinexor EC50 with **(A)** melflufen EC50 (n = 13), **(B)** with melphalan EC50 (n = 13), and **(C)** with bortezomib EC50 (n = 8) values. EC50: half-maximal effective concentration; MM: multiple myeloma.

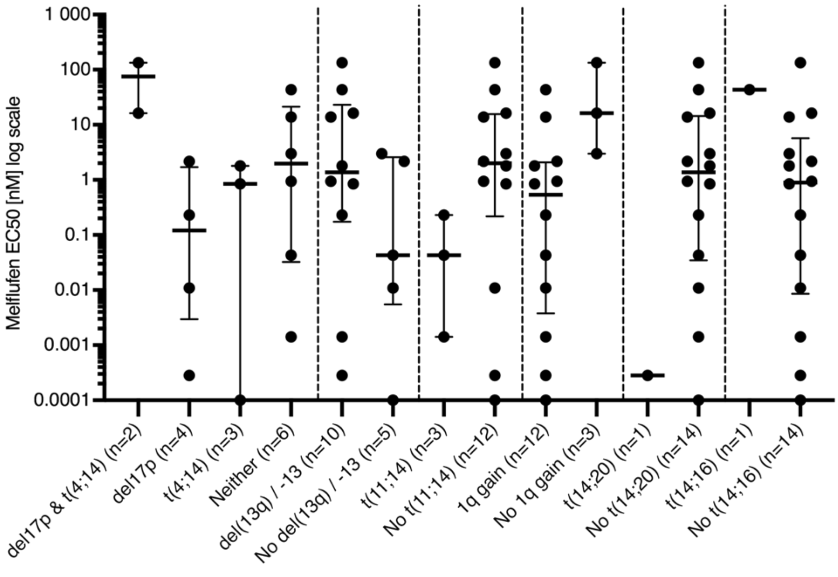

Supplementary Figure S18. Cytogenetics and melflufen sensitivity in CD138+CD38+ plasma cells from MM patient samples. EC50 values were calculated from 15 MM patient samples using multicolor high throughput flow cytometry-based drug sensitivity testing. Samples were grouped based on patient clinical cytogenetics results.

EC50: half-maximal effective concentration; MM: multiple myeloma.

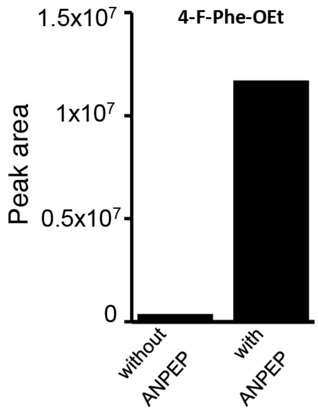

Supplementary Figure S19. Aminopeptidase ANPEP can hydrolyze melflufen to melphalan and 4-F-Phe-OEt. Incubation buffer and substrates were mixed and incubated without and with ANPEP for 2h at 37°C (with duplicate incubations). The incubation was stopped by adding ice cold acetonitrile. 4-F-Phe-OEt was then quantified using LC-HR-MS, with concentrations presented as the peak area in the figure.

4-F-Phe-OEt: para-fluoro-L-phenylalanine ethyl ester; LC-HR-MS: liquid chromatography-high resolution mass spectrometry

SUPPLEMENTARY TABLES

Supplementary Table 1. FIMM dataset patient sample characteristics and sample analysis information (1/15)

Patient sample characteristics													
Patient ID	Sample ID	Sample type	Disease stage	Gender	Age when sample taken	Age at diagnosis	M-component heavy chain type	M-component light chain type	ISS	Clinical sensitivity: Alkylating agents (MEL, CPM)	Clinical sensitivity: Bortezomib	Clinical sensitivity: IMIDs
MM001	MM001	CD138+	RRMM	Female	67	62	IgA	kappa	NA	Exposed	Not exposed	Exposed
MM002	MM002_1	CD138+	RRMM	Female	66	62	IgG	kappa	2	Exposed	Exposed	Exposed
	MM002_2	CD138+	RRMM		68					Exposed	Exposed	Exposed
	MM002_3	CD138+	RRMM		69					Exposed	Refractory	Refractory
MM003	MM003	CD138+	RRMM	Male	76	71	IgA	kappa	2	Exposed	Exposed	Exposed
MM004	MM004	CD138+	RRMM	Female	72	63	IgG	kappa	1	Exposed	Refractory	Exposed
MM005	MM005	CD138+	RRMM	Male	70	67	IgG	lambda	1	Exposed	Refractory	Refractory
MM006	MM006	CD138+	RRMM	Female	71	63	not detected	lambda	2	Exposed	Exposed	Exposed
MM007	MM007	CD138+	RRMM	Male	78	74	not detected	not detected	2	Exposed	Exposed	Not exposed
		BM-MNC										
MM008	MM008	CD138+	RRMM	Female	71	64	IgA	kappa	3	Exposed	Refractory	Exposed
MM009	MM009	CD138+	RRMM	Female	72	64	IgA	kappa	NA	Exposed	Not exposed	Exposed
MM010	MM010	CD138+	RRMM	Male	67	61	NA	kappa	1	Exposed	Not exposed	Exposed
		BM-MNC										
MM011	MM011	CD138+	RRMM	Female	66	57	not detected	lambda	NA	Refractory	Exposed	Refractory
MM012	MM012	BM-MNC	RRMM	Male	80	77	not detected	lambda	1	Refractory	Refractory	Exposed
MM013	MM013	CD138+	RRMM	Male	70	60	IgA	kappa	1	Exposed	Refractory	Refractory
MM014	MM014_1	CD138+	RRMM	Male	71	62	IgA	lambda	NA	Refractory	Refractory	Refractory
	MM014_2	CD138+	RRMM		71					Refractory	Refractory	Refractory
	MM014_3	CD138+	RRMM		71					Refractory	Refractory	Refractory
	MM014_4	CD138+	RRMM		72					Refractory	Refractory	Refractory

Supplementary Table 1. FIMM dataset patient sample characteristics and sample analysis information (2/15)

Patient ID	Sample ID	Sample type	Disease stage	Gender	Age when sample taken	Age at diagnosis	M-component heavy chain type	M-component light chain type	ISS	Clinical sensitivity: Alkylating agents (MEL, CPM)	Clinical sensitivity: Bortezomib	Clinical sensitivity: IMIDs
MM015	MM015	CD138+	RRMM	Male	66	60	not detected	not detected	1	Exposed	Not exposed	Exposed
MM016	MM016	CD138+	RRMM	Male	77	69	IgG	kappa	NA	Exposed	Refractory	Refractory
MM017	MM017_1	CD138+	RRMM	Female	69	60	IgA	lambda	1	Exposed	Exposed	Not exposed
	MM017_2	CD138+	RRMM		70					Exposed	Refractory	Not exposed
		BM-MNC										
	MM017_3	CD138+	RRMM		70					Exposed	Refractory	Refractory
MM018	MM018	CD138+	RRMM	Male	63	44	IgG	kappa	NA	Exposed	Not exposed	Refractory
MM019	MM019	CD138+	RRMM	Male	72	65	IgG	kappa	2	Exposed	Exposed	Refractory
MM020	MM020	CD138+	RRMM	Female	64	55	not detected	lambda	NA	Exposed	Exposed	Exposed
MM021	MM021	BM-MNC	RRMM	Male	47	37	NA	NA	NA	Exposed	Refractory	Refractory
MM022	MM022_1	CD138+	RRMM	Male	72	67	IgA	lambda	3	Exposed	Exposed	Refractory
	MM022_2	CD138+	RRMM		75					Exposed	Exposed	Refractory
	MM022_3	CD138+	RRMM		76					Refractory	Refractory	Refractory
MM023	MM023	CD138+	RRMM	Male	67	50	IgG	kappa	1	Refractory	Refractory	Refractory
MM024	MM024	CD138+	RRMM	Male	56	45	IgG	kappa	NA	Exposed	Refractory	Refractory
MM025	MM025_1	CD138+	RRMM	Male	63	56	IgG	kappa	NA	Exposed	Exposed	Refractory
		BM-MNC										
	MM025_2	CD138+	RRMM		63					Exposed	Refractory	Refractory
MM026	MM026	CD138+	RRMM	Male	73	61	not detected	not detected	1	Exposed	Refractory	Refractory
MM027	MM027	CD138+	RRMM	Female	72	68	NA	NA	NA	Refractory	Refractory	Refractory
MM028	MM028	CD138+	RRMM	Male	65	58	NA	NA	NA	Exposed	Exposed	Not exposed
MM029	MM029	CD138+	RRMM	Female	55	50	IgA	lambda	3	Exposed	Exposed	Exposed
MM030	MM030_1	CD138+	RRMM	Male	72	68	IgA	lambda	3	Refractory	Exposed	Refractory
	MM030_2	CD138+	RRMM		74					Refractory	Refractory	Refractory
MM031	MM031	CD138+	RRMM	Male	81	75	IgG	kappa	2	Exposed	Exposed	Exposed

Supplementary Table 1. FIMM dataset patient sample characteristics and sample analysis information (3/15)

Patient ID	Sample ID	Sample type	Disease stage	Gender	Age when sample taken	Age at diagnosis	M-component heavy chain type	M-component light chain type	ISS	Clinical sensitivity: Alkylating agents (MEL, CPM)	Clinical sensitivity: Bortezomib	Clinical sensitivity: IMIDs
MM032	MM032_1	CD138+	RRMM	Male	66	64	not detected	kappa	1	Not exposed	Not exposed	Not exposed
	MM032_2	CD138+	RRMM		69					Exposed	Exposed	Refractory
MM033	MM033_1	CD138+	RRMM	Male	70	68	IgA	lambda	2	Refractory	Refractory	Exposed
	MM033_2	CD138+	RRMM		71					Refractory	Refractory	Refractory
	MM033_3	CD138+	RRMM		71					Refractory	Refractory	Refractory
	MM033_4	CD138+	RRMM		72					Refractory	Refractory	Refractory
MM034	MM034_1	CD138+	RRMM	Female	68	65	IgG	kappa	1	Exposed	Exposed	Not exposed
	MM034_2	CD138+	RRMM		70					Exposed	Exposed	Refractory
MM035	MM035	CD138+	RRMM	Male	55	52	IgA	kappa	1	Exposed	Exposed	Exposed
MM036	MM036_1	CD138+	RRMM	Male	69	63	IgA	kappa	1	Exposed	Exposed	Not exposed
	MM036_2	CD138+	RRMM		69					Exposed	Exposed	Not exposed
MM037	MM037	CD138+	RRMM	Female	63	58	IgG	kappa	2	Exposed	Exposed	Exposed
		BM-MNC										
MM038	MM038	CD138+	RRMM	Female	69	63	IgG	kappa	2	Exposed	Exposed	Exposed
MM039	MM039	CD138+	RRMM	Female	69	64	IgG	kappa	2	Exposed	Exposed	Refractory
MM040	MM040	CD138+	RRMM	Female	69	68	IgG	lambda	3	Refractory	Refractory	Not exposed
MM041	MM041	CD138+	RRMM	Female	60	59	not detected	kappa	2	Exposed	Exposed	Not exposed
MM042	MM042	CD138+	RRMM	Male	76	75	IgG	kappa	2	Exposed	Exposed	Refractory
MM043	MM043_1	CD138+	RRMM	Female	64	56	IgG	kappa	NA	Exposed	Refractory	Refractory
	MM043_2	CD138+	RRMM		66					Exposed	Refractory	Refractory
MM044	MM044	CD138+	RRMM	Female	71	64	IgG	kappa	3	Exposed	Exposed	Refractory
MM045	MM045	CD138+	RRMM	Male	75	67	IgG	lambda	NA	Exposed	Not exposed	Exposed
MM046	MM046_1	CD138+	RRMM	Male	59	58	not detected	lambda	3	Exposed	Exposed	Refractory
	MM046_2	CD138+	RRMM		59					Exposed	Refractory	Refractory
MM047	MM047	CD138+	RRMM	Male	70	66	IgG	kappa	2	Exposed	Exposed	Exposed

Supplementary Table 1. FIMM dataset patient sample characteristics and sample analysis information (4/15)

Patient ID	Sample ID	Sample type	Disease stage	Gender	Age when sample taken	Age at diagnosis	M-component heavy chain type	M-component light chain type	ISS	Clinical sensitivity: Alkylating agents (MEL, CPM)	Clinical sensitivity: Bortezomib	Clinical sensitivity: IMIDs
MM048	MM048_1	CD138+	RRMM	Male	69	59	IgA	kappa	NA	Exposed	Refractory	Exposed
	MM048_2	CD138+	RRMM		72					Exposed	Refractory	Exposed
MM049	MM049	CD138+	RRMM	Male	62	55	IgG	kappa	2	Not exposed	Exposed	Exposed
MM050	MM050	CD138+	RRMM	Female	70	68	IgG	kappa	1	Exposed	Refractory	Refractory
MM051	MM051_1	CD138+	NDMM (not treated)	Male	56	56	IgA	kappa	1	Not exposed	Not exposed	Not exposed
	MM051_2	CD138+	RRMM		58					Exposed	Exposed	Refractory
MM052	MM052_1	CD138+	RRMM	Male	60	59	IgA	lambda	3	Exposed	Exposed	Refractory
	MM052_2	CD138+	RRMM		61					Exposed	Exposed	Refractory
MM053	MM053	CD138+	NDMM (not treated)	Female	51	51	IgG	kappa	1	Not exposed	Not exposed	Not exposed
MM054	MM054	CD138+	RRMM	Female	82	76	IgG	kappa	1	Exposed	Not exposed	Refractory
MM055	MM055	CD138+	RRMM	Male	72	66	IgG	kappa	2	Refractory	Exposed	Exposed
MM056	MM056_1	CD138+	NDMM (not treated)	Female	69	69	IgA	kappa	1	Not exposed	Not exposed	Not exposed
	MM056_2	CD138+	RRMM		71					Exposed	Exposed	Refractory
MM057	MM057_1	CD138+	RRMM	Male	57	56	not detected	kappa	1	Exposed	Exposed	Not exposed
	MM057_2	CD138+	RRMM		58					Exposed	Refractory	Refractory
	MM057_3	CD138+	RRMM		58					Exposed	Refractory	Refractory
	MM057_4	CD138+	RRMM		59					Refractory	Refractory	Refractory
	MM057_5	CD138+	RRMM		59					Refractory	Refractory	Refractory
	MM057_6	CD138+	RRMM		60					Refractory	Refractory	Refractory
MM058	MM058_1	CD138+	RRMM	Male	64	60	IgG	kappa	1	Exposed	Refractory	Refractory
	MM058_2	CD138+	RRMM		65					Exposed	Refractory	Refractory
MM059	MM059	CD138+	RRMM	Female	58	56	IgG	kappa	2	Exposed	Exposed	Refractory
MM060	MM060	CD138+	RRMM	Male	61	57	IgG	kappa	3	Exposed	Exposed	Not exposed
MM061	MM061_1	CD138+	RRMM	Male	52	49	IgG	kappa	NA	Exposed	Refractory	Refractory
	MM061_2	CD138+	RRMM		53					Exposed	Refractory	Refractory

Supplementary Table 1. FIMM dataset patient sample characteristics and sample analysis information (5/16)

Patient ID	Sample ID	Sample type	Disease stage	Gender	Age when sample taken	Age at diagnosis	M-component heavy chain type	M-component light chain type	ISS	Clinical sensitivity: Alkylating agents (MEL, CPM)	Clinical sensitivity: Bortezomib	Clinical sensitivity: IMIDs
MM062	MM062	CD138+	NDMM (not treated)	Male	68	68	IgA	lambda	NA	Not exposed	Not exposed	Not exposed
MM063	MM063	CD138+	NDMM (<30d treated)	Male	66	66	not detected	kappa	1	Not exposed	Not exposed	Not exposed
MM064	MM064	CD138+	NDMM (not treated)	Female	68	68	IgG	kappa	3	Not exposed	Not exposed	Not exposed
MM065	MM065	CD138+	NDMM (not treated)	Male	61	61	NA	kappa	2	Not exposed	Not exposed	Not exposed
MM066	MM066	CD138+	NDMM (not treated)	Male	67	67	IgG	lambda	3	Not exposed	Not exposed	Not exposed
MM067	MM067	CD138+	NDMM (not treated)	Male	63	63	IgG	kappa	2	Not exposed	Not exposed	Not exposed
MM068	MM068_1	CD138+	RRMM	Female	74	74	not detected	kappa	1	Not exposed	Exposed	Not exposed
	MM068_2	CD138+	RRMM		77					Refractory	Exposed	Refractory
MM069	MM069	CD138+	NDMM (not treated)	Male	55	51	not detected	kappa	1	Not exposed	Not exposed	Not exposed
MM070	MM070	CD138+	NDMM (not treated)	Female	55	55	IgA	lambda	2	Not exposed	Not exposed	Not exposed
MM071	MM071	CD138+	RRMM	Male	68	65	IgG	kappa	3	Exposed	Exposed	Refractory
MM072	MM072	CD138+	NDMM (not treated)	Male	63	63	IgG	kappa	2	Not exposed	Not exposed	Not exposed
MM073	MM073	CD138+	RRMM	Female	78	72	IgG	kappa	2	Exposed	Not exposed	Exposed
MM074	MM074	CD138+	NDMM (not treated)	Female	59	59	IgG	lambda	2	Not exposed	Not exposed	Not exposed
MM075	MM075	CD138+	NDMM (not treated)	Male	71	71	IgA	kappa	2	Not exposed	Not exposed	Not exposed
MM076	MM076	CD138+	NDMM (not treated)	Female	59	59	IgG	kappa	2	Not exposed	Not exposed	Not exposed
MM077	MM077	CD138+	RRMM	Female	66	65	IgA	lambda	3	Refractory	Exposed	Exposed
MM078	MM078_1	CD138+	NDMM (<30d treated)	Male	66	66	not detected	not detected	3	Not exposed	Not exposed	Not exposed
	MM078_2	CD138+	RRMM		67					Exposed	Exposed	Not exposed
MM079	MM079	CD138+	NDMM (not treated)	Female	61	61	IgG	kappa	NA	Not exposed	Not exposed	Not exposed
MM080	MM080	CD138+	RRMM	Male	84	81	IgG	kappa	3	Exposed	Refractory	Refractory
MM081	MM081	CD138+	RRMM	Female	75	66	IgA	lambda	NA	Exposed	Exposed	Not exposed
MM082	MM082	CD138+	RRMM	Female	58	55	IgG	kappa	NA	Exposed	Exposed	Exposed
		BM-MNC										
MM083	MM083_1	CD138+	NDMM (<30d treated)	Male	66	66	IgG	kappa	2	Not exposed	Not exposed	Not exposed
	MM083_2	CD138+	RRMM		67					Exposed	Exposed	Not exposed

Supplementary Table 1. FIMM dataset patient sample characteristics and sample analysis information (6/15)

Patient ID	Sample ID	Sample type	Disease stage	Gender	Age when sample taken	Age at diagnosis	M-component heavy chain type	M-component light chain type	ISS	Clinical sensitivity: Alkylating agents (MEL, CPM)	Clinical sensitivity: Bortezomib	Clinical sensitivity: IMIDs
MM084	MM084	CD138+	RRMM	Male	68	66	IgG	lambda	2	Exposed	Exposed	Exposed
MM085	MM085	CD138+	NDMM (not treated)	Male	66	66	IgG	kappa	2	Not exposed	Not exposed	Not exposed
MM086	MM086	CD138+	NDMM (not treated)	Male	64	64	ei tutkittu	ei tutkittu	3	Not exposed	Not exposed	Not exposed
MM087	MM087	CD138+<br>BM-MNC	NDMM (not treated)	Female	70	70	not detected	not detected	3	Not exposed	Not exposed	Not exposed
MM088	MM088	CD138+	NDMM (not treated)	Female	54	54	IgA	lambda	2	Not exposed	Not exposed	Not exposed
MM089	MM089	CD138+	NDMM (not treated)	Male	46	46	not detected	kappa	1	Not exposed	Not exposed	Not exposed
MM090	MM090	CD138+	NDMM (not treated)	Female	69	69	IgA	lambda	2	Not exposed	Not exposed	Not exposed
MM091	MM091	CD138+	RRMM	Male	61	55	IgG	kappa	NA	Exposed	Exposed	Refractory
MM092	MM092	CD138+	RRMM	Male	75	71	IgG	lambda	2	Exposed	Not exposed	Exposed
MM093	MM093	CD138+	NDMM (<30d treated)	Male	58	58	IgA	lambda	2	Not exposed	Not exposed	Not exposed
MM094	MM094	CD138+	NDMM (not treated)	Female	67	67	IgG	kappa	2	Not exposed	Not exposed	Not exposed
MM095	MM095	CD138+	RRMM	Male	49	41	IgG	lambda	NA	Exposed	Refractory	Refractory
MM096	MM096	CD138+	NDMM (<30d treated)	Female	69	69	not detected	not detected	NA	Not exposed	Not exposed	Not exposed
MM097	MM097	CD138+	RRMM	Female	77	75	not detected	kappa	2	Exposed	Exposed	Not exposed
MM098	MM098	CD138+	RRMM	Male	57	57	IgG	lambda	3	Refractory	Refractory	Exposed
MM099	MM099	CD138+	RRMM	Male	80	78	IgA	kappa	3	Refractory	Refractory	Refractory
MM100	MM100	CD138+	NDMM (not treated)	Male	53	53	not detected	kappa	1	Not exposed	Not exposed	Not exposed
MM101	MM101	CD138+	NDMM (not treated)	Male	65	64	IgA	kappa	2	Not exposed	Not exposed	Not exposed
MM102	MM102	CD138+	RRMM	Male	68	66	NA	NA	NA	Exposed	Exposed	Exposed
MM103	MM103	CD138+	RRMM	Male	66	66	not detected	kappa	1	Not exposed	Not exposed	Not exposed
MM104	MM104	CD138+	NDMM (not treated)	Female	66	66	not detected	kappa	1	Not exposed	Not exposed	Not exposed
MM105	MM105	CD138+	NDMM (not treated)	Female	76	76	IgA	kappa	1	Not exposed	Not exposed	Not exposed
MM106	MM106	BM-MNC	NDMM (not treated)	Male	58	58	IgG	kappa	NA	Not exposed	Not exposed	Not exposed
MM107	MM107	CD138+	RRMM	Female	67	66	IgG	kappa	NA	Exposed	Exposed	Not exposed

Supplementary Table 1. FIMM dataset patient sample characteristics and sample analysis information (7/15)

Patient ID	Sample ID	Sample type	Disease stage	Gender	Age when sample taken	Age at diagnosis	M-component heavy chain type	M-component light chain type	ISS	Clinical sensitivity: Alkylating agents (MEL, CPM)	Clinical sensitivity: Bortezomib	Clinical sensitivity: IMIDs
MM108	MM108	CD138+	NDMM (not treated)	Male	73	73	IgG	lambda	2	Not exposed	Not exposed	Not exposed
MM109	MM109	CD138+	RRMM	Male	72	65	IgG	kappa	2	Refractory	Refractory	Refractory
MM110	MM110	CD138+	NDMM (not treated)	Female	84	84	IgA	lambda	3	Not exposed	Not exposed	Not exposed
MM111	MM111_1	CD138+	RRMM	Male	60	60	IgA	lambda	3	Exposed	Refractory	Exposed
	MM111_2	CD138+	RRMM		61					Exposed	Refractory	Exposed
MM112	MM112	BM-MNC	NDMM (not treated)	Female	71	71	IgG	kappa	2	Not exposed	Not exposed	Not exposed
MM113	MM113_1	CD138+	NDMM (not treated)	Male	58	58	IgG	kappa	3	Not exposed	Not exposed	Not exposed
	MM113_2	CD138+	RRMM		59					Exposed	Exposed	Exposed
MM114	MM114	CD138+	NDMM (<30d treated)	Female	61	61	not detected	kappa	2	Not exposed	Not exposed	Not exposed
MM115	MM115	CD138+	NDMM (<30d treated)	Female	59	59	not detected	not detected	2	Not exposed	Not exposed	Not exposed
MM116	MM116	CD138+	NDMM (not treated)	Female	55	55	not detected	kappa	2	Not exposed	Not exposed	Not exposed
MM117	MM117_1	CD138+	NDMM (not treated)	Male	51	51	IgG	kappa	3	Not exposed	Not exposed	Not exposed
	MM117_2	CD138+	RRMM		53					Exposed	Exposed	Exposed
MM118	MM118	CD138+	NDMM (not treated)	Male	63	62	IgA	kappa	2	Not exposed	Not exposed	Not exposed
MM119	MM119	CD138+	NDMM (not treated)	Female	75	73	NA	NA	NA	Not exposed	Not exposed	Not exposed
MM120	MM120	CD138+	NDMM (<30d treated)	Female	69	69	IgG	kappa	2	Not exposed	Not exposed	Not exposed
MM121	MM121	CD138+	NDMM (not treated)	Female	79	78	IgG	kappa	NA	Not exposed	Not exposed	Not exposed
MM122	MM122	CD138+	NDMM (not treated)	Male	68	67	IgG	lambda	NA	Not exposed	Not exposed	Not exposed
MM123	MM123	CD138+	NDMM (not treated)	Female	61	61	NA	NA	2	Not exposed	Not exposed	Not exposed
MM124	MM124	CD138+	RRMM	Male	76	73	IgA	lambda	NA	Refractory	Refractory	Refractory
		BM-MNC										
MM125	MM125	BM-MNC	NDMM (not treated)	Male	55	55	IgG	lambda	2	Not exposed	Not exposed	Not exposed
MM126	MM126	CD138+	NDMM (not treated)	Female	80	80	IgG	lambda	3	Not exposed	Not exposed	Not exposed
MM127	MM127	BM-MNC	NDMM (not treated)	Female	69	69	not detected	kappa	3	Not exposed	Not exposed	Not exposed
MM128	MM128_1	CD138+	NDMM (not treated)	Male	77	77	IgA	kappa	2	Not exposed	Not exposed	Not exposed
		BM-MNC										

Supplementary Table 1. FIMM dataset patient sample characteristics and sample analysis information (8/15)

Patient ID	Sample ID	Sample type	Disease stage	Gender	Age when sample taken	Age at diagnosis	M-component heavy chain type	M-component light chain type	ISS	Clinical sensitivity: Alkylating agents (MEL, CPM)	Clinical sensitivity: Bortezomib	Clinical sensitivity: IMIDs
MM128	MM128_2	BM-MNC	RRMM	Male	79	77	IgA	kappa	2	Not exposed	Exposed	Refractory
MM129	MM129	CD138+	RRMM	Male	59	59	IgA	kappa	3	Refractory	Refractory	Not exposed
MM130	MM130	CD138+	RRMM	Female	56	51	IgA	lambda	NA	Exposed	Exposed	Exposed
MM131	MM131	CD138+	NDMM (not treated)	Male	63	63	IgA	lambda	2	Not exposed	Not exposed	Not exposed
MM132	MM132	CD138+	NDMM (not treated)	Male	74	68	ei tutkittu	ei tutkittu	NA	Not exposed	Not exposed	Not exposed
MM133	MM133	CD138+	NDMM (not treated)	Male	75	70	IgA	lambda	1	Not exposed	Not exposed	Not exposed
MM134	MM134	CD138+	NDMM (not treated)	Male	50	50	IgG	kappa	3	Not exposed	Not exposed	Not exposed
MM135	MM135	CD138+<br>BM-MNC	RRMM	Male	61	60	IgA	lambda	2	Exposed	Exposed	Refractory
MM136	MM136	CD138+	RRMM	Female	26	26	not detected	kappa	2	Exposed	Exposed	Not exposed
MM137	MM137	CD138+	RRMM	Male	70	68	NA	NA	NA	Refractory	Refractory	Not exposed
MM138	MM138	CD138+	NDMM (not treated)	Female	65	65	not detected	kappa	2	Not exposed	Not exposed	Not exposed
MM139	MM139	CD138+	NDMM (not treated)	Female	77	77	IgA	kappa	2	Not exposed	Not exposed	Not exposed
MM140	MM140	CD138+	NDMM (not treated)	Male	50	50	IgG	kappa	1	Not exposed	Not exposed	Not exposed

Supplementary Table 1. FIMM dataset patient sample characteristics and sample analysis information (9/15)

	Patient sample characteristics (cytogenetics)								Patient sample analysis information				
Sample ID	t(11;14)	t(4;14)	t(14;16)	t(14;20)	del17p	del(13q) / -13	1q gain	No findings	RNA sequencing	LC-MS/MS Proteomics	Exome sequencing	Copy number analysis	Flow cytometry-based drug sensitivity testing
MM001	0	1	0	0	1	1	1	0	0	0	1	1	0
MM002_1	0	0	0	0	1	0	0	0	1	0	1	1	0
MM002_2	0	0	0	0	1	0	0	0	1	0	1	1	0
MM002_3	0	0	0	0	1	0	0	0	1	0	1	1	0
MM003	0	1	0	0	0	0	0	0	1	1	1	1	0
MM004	0	0	0	0	1	1	0	0	0	0	1	1	0
MM005	0	0	0	1	0	0	1	0	1	0	1	1	0
MM006	0	0	1	0	1	1	1	0	1	0	1	1	0
MM007	1	0	0	0	0	1	1	0	1	1	1	1	0
									0	0	0	0	1
MM008	1	0	0	0	0	0	0	0	1	0	1	1	0
MM009	0	0	0	0	0	1	0	0	0	0	1	1	0
MM010	0	0	0	0	1	0	1	0	1	0	1	1	0
									0	0	0	0	1
MM011	1	0	0	0	0	1	1	0	1	1	1	1	0
MM012	1	0	0	0	1	1	1	0	0	0	0	0	1
MM013	0	1	0	0	0	1	1	0	0	0	1	1	0
MM014_1	0	1	0	0	0	1	1	0	0	0	1	1	0
MM014_2	0	1	0	0	0	1	1	0	1	0	1	1	0
MM014_3	0	1	0	0	0	1	1	0	0	0	1	1	0
MM014_4	0	1	0	0	0	1	1	0	1	0	1	1	0
MM015	0	0	0	0	0	1	0	0	0	0	1	1	0
MM016	0	1	0	0	0	1	1	0	0	0	1	1	0
MM017_1	0	0	0	0	0	1	1	0	1	0	1	1	0
MM017_2	0	0	0	0	0	1	1	0	1	0	1	1	0
MM017_3	0	0	0	0	0	1	1	0	1	0	1	1	0
MM018	1	0	0	0	0	1	1	0	1	0	0	0	0
MM019	0	0	0	0	0	0	0	0	1	0	1	1	0
MM020	0	0	0	0	0	0	1	0	1	1	1	1	0

Supplementary Table 1. FIMM dataset patient sample characteristics and sample analysis information (10/15)

Sample ID	t(11;14)	t(4;14)	t(14;16)	t(14;20)	del17p	del(13q) / -13	1q gain	No findings	RNA sequencing	LC-MS/MS Proteomics	Exome sequencing	Copy number analysis	Flow cytometry-based drug sensitivity testing
MM021	0	1	0	0	0	0	1	0	0	0	0	0	1
MM022_1	0	0	0	0	0	1	0	0	1	0	1	1	0
MM022_2	0	0	0	0	1	1	1	0	0	0	1	1	0
MM022_3	0	0	0	0	1	1	1	0	1	0	1	1	0
MM023	0	0	0	0	0	0	0	0	0	0	1	1	0
MM024	0	0	0	0	0	0	0	0	1	0	1	1	0
MM025_1	0	0	0	0	1	0	1	0	1	1	1	1	0
									0	0	0	0	1
MM025_2	0	0	0	0	1	0	1	0	0	0	1	1	0
MM026	0	0	0	0	0	1	1	0	1	0	1	1	0
MM027	0	0	0	0	0	0	1	0	1	0	1	1	0
MM028	1	0	0	0	0	0	0	0	1	0	1	1	0
MM029	1	0	0	0	0	0	1	0	0	0	1	1	0
MM030_1	0	0	0	0	0	1	1	0	1	1	1	1	0
MM030_2	0	0	0	0	0	1	1	0	1	0	1	1	0
MM031_2	1	0	0	0	0	0	0	0	1	0	1	1	0
MM032_1	0	0	0	0	0	0	0	0	1	1	1	1	0
MM032_2	0	0	0	0	0	0	0	0	0	0	1	1	0
MM033_1	0	0	0	0	0	1	1	0	1	0	1	1	0
MM033_2	0	0	0	0	0	1	1	0	1	1	1	1	0
MM033_3	0	0	0	0	0	1	1	0	0	0	1	1	0
MM033_4	0	0	0	0	1	1	1	0	1	0	1	1	0
MM034_1	0	0	0	0	0	0	0	0	1	0	1	1	0
MM034_2	0	0	0	0	0	0	0	0	1	0	1	1	0
MM035	0	1	0	0	0	1	1	0	0	0	1	1	0
MM036_1	0	0	0	0	0	0	1	0	1	0	1	1	0
MM036_2	0	0	0	0	0	0	1	0	1	0	1	1	0
MM037	0	0	0	1	1	1	1	0	1	0	1	1	0
									0	0	0	0	1

Supplementary Table 1. FIMM dataset patient sample characteristics and sample analysis information (11/15)

Sample ID	t(11;14)	t(4;14)	t(14;16)	t(14;20)	del17p	del(13q) / -13	1q gain	No findings	RNA sequencing	LC-MS/MS Proteomics	Exome sequencing	Copy number analysis	Flow cytometry-based drug sensitivity testing
MM038	0	0	0	0	1	1	1	0	1	0	1	1	0
MM039	0	0	0	0	0	0	0	0	1	0	1	1	0
MM040	0	0	1	0	1	1	1	0	0	0	1	1	0
MM041	1	0	0	0	1	0	0	0	1	1	1	1	0
MM042	0	0	0	0	1	0	0	0	1	0	1	1	0
MM043_1	1	0	0	0	0	0	0	0	1	1	1	1	0
MM043_2	1	0	0	0	1	1	1	0	1	0	1	1	0
MM044	0	1	0	0	0	0	0	0	1	0	1	1	0
MM045	0	0	0	0	0	1	0	0	1	1	1	1	0
MM046_1	0	0	0	0	0	0	0	0	1	1	1	1	0
MM046_2	0	0	1	0	0	0	0	0	1	0	1	1	0
MM047	0	0	0	0	1	0	0	0	0	0	1	1	0
MM048_1	0	0	0	0	0	0	0	0	0	0	1	1	0
MM048_2	0	0	0	0	0	0	0	0	1	0	1	1	0
MM049	0	0	0	0	0	1	0	0	1	0	1	1	0
MM050	0	1	0	0	0	1	1	0	1	0	1	1	0
MM051_1	0	1	0	0	0	1	1	0	1	0	1	1	0
MM051_2	0	1	0	0	0	0	0	0	1	0	1	1	0
MM052_1	1	0	0	0	0	0	1	0	0	0	1	1	0
MM052_2	1	0	0	0	0	0	1	0	0	0	1	1	0
MM053	0	0	0	0	0	1	0	0	1	0	1	1	0
MM054	0	1	0	0	0	1	1	0	1	0	1	1	0
MM055	0	0	0	0	0	1	0	0	0	0	1	1	0
MM056_1	0	0	0	0	0	1	1	0	1	0	1	1	0
MM056_2	0	0	0	0	0	0	1	0	1	1	1	1	0
MM057_1	0	0	0	0	0	0	1	0	1	1	1	1	0
MM057_2	0	0	0	0	0	0	1	0	1	0	1	1	0
MM057_3	0	0	0	0	0	0	0	0	1	0	1	1	0
MM057_4	0	0	0	0	0	0	1	0	1	0	1	1	0
MM057_5	0	0	0	0	0	0	1	0	1	0	1	1	0

Supplementary Table 1. FIMM dataset patient sample characteristics and sample analysis information (12/15)

Sample ID	t(11;14)	t(4;14)	t(14;16)	t(14;20)	del17p	del(13q) / -13	1q gain	No findings	RNA sequencing	LC-MS/MS Proteomics	Exome sequencing	Copy number analysis	Flow cytometry-based drug sensitivity testing
MM057_6	0	0	0	0	0	0	1	0	1	0	1	1	0
MM058_1	0	1	0	0	0	0	1	0	1	0	1	1	0
MM058_2	0	1	0	0	0	0	1	0	0	0	1	1	0
MM059	0	1	0	0	0	1	1	0	1	0	1	1	0
MM060	0	0	0	0	0	1	1	0	1	0	1	1	0
MM061_1	0	1	0	0	0	1	1	0	1	0	1	1	0
MM061_2	0	1	0	0	1	1	1	0	0	0	1	1	0
MM062	0	0	0	0	0	0	0	1	0	0	1	1	0
MM063	0	0	0	0	0	1	0	0	1	0	1	1	0
MM064	0	0	0	0	0	1	0	0	1	1	1	1	0
MM065	0	0	0	0	1	0	1	0	1	1	1	1	0
MM066	0	0	0	0	0	1	1	0	1	1	1	1	0
MM067	0	0	0	0	0	0	0	0	1	1	1	1	0
MM068_1	1	0	0	0	0	1	0	0	1	0	1	1	0
MM068_2	1	0	0	0	0	1	0	0	1	0	1	1	0
MM069	0	0	0	0	1	0	0	0	0	0	1	1	0
MM070	0	0	0	0	0	1	0	0	1	1	1	1	0
MM071	0	0	0	0	0	1	1	0	1	0	1	1	0
MM072	0	0	0	0	0	1	0	0	0	0	1	1	0
MM073	0	0	0	0	1	0	0	0	0	0	1	1	0
MM074	1	0	0	0	0	1	0	0	1	1	1	1	0
MM075	0	0	0	0	0	0	0	0	1	1	1	1	0
MM076	0	0	0	0	0	0	0	0	0	0	1	1	0
MM077	0	1	0	0	1	1	1	0	0	0	1	1	0
MM078_1	0	0	0	0	0	0	0	0	1	1	1	1	0
MM078_2	0	0	0	0	0	0	0	0	0	0	1	1	0
MM079	0	0	0	0	0	0	0	0	0	0	1	1	0
MM080	0	0	0	0	0	0	0	0	1	0	1	1	0
MM081	0	1	0	0	1	1	1	0	1	0	1	1	0
MM082	0	0	0	0	0	0	0	0	1	0	1	1	0
									0	0	0	0	1

Supplementary Table 1. FIMM dataset patient sample characteristics and sample analysis information (13/15)

Sample ID	t(11;14)	t(4;14)	t(14;16)	t(14;20)	del17p	del(13q) / -13	1q gain	No findings	RNA sequencing	LC-MS/MS Proteomics	Exome sequencing	Copy number analysis	Flow cytometry-based drug sensitivity testing
MM083_1	0	0	0	0	0	0	0	1	0	0	1	1	0
MM083_2	0	0	0	0	0	0	0	1	0	0	1	1	0
MM084	0	0	0	0	0	1	0	0	0	0	1	1	0
MM085	0	0	0	0	0	1	0	0	1	0	1	1	0
MM086	1	0	0	0	1	1	0	0	1	0	1	1	0
MM087	0	0	0	0	0	1	1	0	1	0	1	1	0
									0	0	0	0	1
MM088	0	0	0	0	0	1	0	0	1	0	1	1	0
MM089	1	0	0	0	0	0	0	0	1	0	1	1	0
MM090	1	0	0	0	0	1	0	0	1	0	1	1	0
MM091	0	0	0	0	0	1	1	0	1	0	1	1	0
MM092	1	0	0	0	0	0	0	0	0	0	1	1	0
MM093	0	0	0	0	0	1	0	0	1	0	1	1	0
MM094	0	0	0	0	0	0	0	0	1	0	1	1	0
MM095	1	0	0	0	1	1	1	0	1	1	1	1	0
MM096	1	0	0	0	0	1	0	0	1	0	1	1	0
MM097	0	0	0	0	0	0	1	0	0	0	1	1	0
MM098	0	0	0	0	1	0	1	0	1	0	1	1	0
MM099	0	1	0	0	1	1	0	0	0	0	1	1	0
MM100	1	0	0	0	0	1	0	0	0	0	1	1	0
MM101	0	0	0	0	0	1	1	0	1	0	1	1	0
MM102	0	0	0	0	0	0	1	0	1	0	1	1	0
MM103	0	0	0	0	0	1	0	0	0	0	1	1	0
MM104	1	0	0	0	0	1	1	0	0	0	1	1	0
MM105	0	0	0	0	0	1	0	0	0	0	1	1	0
MM106	0	1	0	0	0	1	1	0	0	0	0	0	1
MM107	0	0	0	0	0	0	0	0	0	0	1	1	0
MM108	0	1	0	0	0	1	1	0	0	0	1	1	0
MM109	0	0	0	0	0	1	0	0	1	0	1	1	0
MM110	1	0	0	0	0	1	1	0	0	0	1	1	0

Supplementary Table 1. FIMM dataset patient sample characteristics and sample analysis information (14/15)

Sample ID	t(11;14)	t(4;14)	t(14;16)	t(14;20)	del17p	del(13q) / -13	1q gain	No findings	RNA sequencing	LC-MS/MS Proteomics	Exome sequencing	Copy number analysis	Flow cytometry-based drug sensitivity testing
MM111_1	0	1	0	0	0	1	1	0	1	0	1	1	0
MM111_2	0	1	0	0	1	1	1	0	1	0	1	1	0
MM112	0	0	0	0	0	1	1	0	0	0	0	0	1
MM113_1	0	0	0	0	0	1	0	0	0	0	1	1	0
MM113_2	0	0	0	0	0	1	0	0	1	0	1	1	0
MM114	0	0	0	0	1	1	1	0	1	0	1	1	0
MM115	1	0	0	0	0	1	0	0	1	0	1	1	0
MM116	0	0	0	0	0	1	0	0	0	0	1	1	0
MM117_1	0	1	0	0	0	1	0	0	1	0	1	1	0
MM117_2	0	1	0	0	0	1	1	0	1	0	1	1	1
MM118	0	1	0	0	0	1	1	0	1	0	1	1	0
MM119	0	0	1	0	0	1	1	0	1	0	1	1	0
MM120	0	0	0	0	0	0	0	0	0	0	1	1	0
MM121	1	0	0	0	0	0	1	0	1	0	1	1	0
MM122	0	0	0	0	0	0	0	0	1	0	1	1	0
MM123	1	0	0	0	0	0	0	0	1	0	1	1	0
MM124	1	0	0	0	0	0	1	0	1	0	1	1	0
									0	0	0	0	1
MM125	0	1	0	0	0	1	1	0	0	0	0	0	1
MM126	1	0	0	0	0	1	0	0	1	0	1	1	0
MM127	0	0	1	0	0	1	1	0	0	0	0	0	1
MM128_1	0	1	0	0	1	1	0	0	1	0	1	1	0
									0	0	0	0	1
MM128_2	0	1	0	0	1	1	0	0	0	0	0	0	1
MM129	0	1	0	0	0	1	1	0	1	0	1	1	0
MM130	0	0	0	0	0	1	1	0	0	0	1	1	0
MM131	1	0	0	0	0	1	0	0	1	0	1	1	0
MM132	0	1	0	0	0	0	0	0	1	0	1	1	0
MM133	0	1	0	0	0	0	0	0	1	0	1	1	0
MM134	0	0	0	0	0	1	1	0	1	0	1	1	0

Supplementary Table 1. FIMM dataset patient sample characteristics and sample analysis information (15/15)

Sample ID	t(11;14)	t(4;14)	t(14;16)	t(14;20)	del17p	del(13q) / -13	1q gain	No findings	RNA sequencing	LC-MS/MS Proteomics	Exome sequencing	Copy number analysis	Flow cytometry-based drug sensitivity testing
MM135	0	0	0	0	1	0	0	0	1	0	1	1	1
MM136	1	0	0	0	0	0	0	0	1	0	1	1	0
MM137	0	1	0	0	0	0	1	0	1	0	1	1	0
MM138	0	0	0	0	0	1	0	0	1	0	1	1	0
MM139	1	0	0	0	0	1	0	0	1	0	1	1	0
MM140	1	0	0	0	0	0	0	0	1	0	1	1	0

Some of the samples have two different sample types indicated for the same sample. In these samples the CD138+ cells have been selected from the BM-MNCs directly after sampling from the MM patient. The BM-MNCs indicated in the table are from the same sample as the CD138+ cells, but the BM-MNCs have been viably frozen and used later for the *ex vivo* drug sensitivity testing experiments.

BM: bone marrow mononuclear cell; NDMM: newly diagnosed multiple myeloma; RRMM: relapsed/refractory multiple myeloma; ISS: international staging system; MEL: melphalan; CPM: cyclophosphamide IMiD: immunomodulatory drug; LC-MS/MS: liquid chromatography-tandem mass spectrometry.

Supplementary table S2. List of the 39 annotated aminopeptidase genes in the human genome utilizing the Ensembl and NCBI databases and further confirming the molecular function (gene ontology) of the identified genes. (1/3)

Gene symbol	Gene name	ENSEMBL ID	UniProt ID	Protein/ peptidase family	Protein cellular compartment	Aminopeptidase gene expression-based clustering as indicated in Fig. 1 (Cluster no.)	MM poor prognosis indicators (both in FIMM & CoMMpass datasets)	Protein detected with LC-MS/MS in MM patient samples
LAP3	leucine aminopeptidase 3	ENSG00000002549	P28838	M	Cytoplasm, Nucleoplasm, Nucleus, Cytosol	1	0	1
ERAP2	endoplasmic reticulum aminopeptidase 2	ENSG00000164308	Q6P179	M	ER, Cytoplasm	1	0	1
METAP2	methionyl aminopeptidase 2	ENSG00000111142	P50579	M	Cytoplasm	1	0	1
TPP2	tripeptidyl peptidase 2	ENSG00000134900	P29144	S	Nucleus, Cytoplasm	1	0	1
DPP7	dipeptidyl peptidase 7	ENSG00000176978	Q9UHL4	S	Golgi, Cytosol	1	0	1
ERAP1	endoplasmic reticulum aminopeptidase 1	ENSG00000164307	Q9NZ08	M	ER, Cytosol	1	0	1
LTA4H	leukotriene a4 hydrolase	ENSG00000111144	P09960	M	Cytoplasm, Nucleoplasm, Nucleus, Cytosol	1	0	1
LNPEP	leucyl and cystinyl aminopeptidase	ENSG00000113441	Q9UIQ6	M	Cytoplasm	1	0	1
XPNPEP1	X-prolyl aminopeptidase 1	ENSG00000108039	Q9NQW7	M	Cytoplasm	2	1	1
METAP1	methionyl aminopeptidase 1	ENSG00000164024	P53582	M	Cytoplasm	2	0	1
DPP3	dipeptidyl peptidase 3	ENSG00000254986	Q9NY33	M	Cytosol	2	1	1
DPP8	dipeptidyl peptidase 8	ENSG00000074603	Q6V1X1	S	Cytoplasm	2	0	0
NPEPPS	aminopeptidase puromycin sensitive	ENSG00000141279	P55786	M	Cytoplasm, Nucleus	2	0	1
BLMH	bleomycin hydrolase	ENSG00000108578	Q13867	C	Cytoplasm, Nucleus	2	1	1
JMJD7	jumonji domain containing 7	ENSG00000243789	P0C870	*	Nucleus	2	0	0
RNPEP	arginyl aminopeptidase	ENSG00000176393	Q9H4A4	M	Pklasma membrane, Cytoplasm	2	1	1
PGPEP1	pyroglutamyl-peptidase I	ENSG00000130517	Q9NXJ5	C	Cytoplasm	2	0	1
TPP1	tripeptidyl peptidase 1	ENSG00000166340	O14773	S	Golgi, Lysosome, Cytoplasm	2	0	1

C: cysteine peptidase; M: metallopeptidase; S: serine peptidase; * Jumonji oxygenase family

Supplementary table S2. List of the 39 annotated aminopeptidase genes in the human genome utilizing the Ensembl and NCBI databases and further confirming the molecular function (gene ontology) of the identified genes. (2/3)

Gene symbol	Gene name	ENSEMBL ID	UniProt ID	Protein/ peptidase family	Protein cellular compartment	Aminopeptidase gene expression-based clustering as indicated in Fig. 1 (Cluster no.)	MM poor prognosis indicators (both in FIMM & CoMMpass datasets)	Protein detected with LC-MS/MS in MM patient samples
PEPD	peptidase D	ENSG00000124299	P12955	M	Extracellular region or secreted	2	0	1
DNPEP	aspartyl aminopeptidase	ENSG00000123992	Q9ULA0	M	Nucleus, Cytosol	2	0	1
DPP9	dipeptidyl peptidase 9	ENSG00000142002	Q86TI2	S	Cytosol, Nucleus	2	0	0
NPEPL1	aminopeptidase-like 1	ENSG00000215440	Q8NDH3	M	Nucleus, Cytoplasm	3	0	0
METAP1D	methionyl aminopeptidase type 1D, mitochondrial	ENSG00000172878	Q6UB28	M	Mitochondrion	3	0	0
CTSH	cathepsin H	ENSG00000103811	P09668	C	Lysosome, Cytoplasm	3	0	0
XPNPEP3	X-prolyl aminopeptidase 3	ENSG00000196236	Q9NQH7	M	Cytoplasm, Mitochondrion	3	0	0
RNPEPL1	aminopeptidase RNPEPL1	ENSG00000142327	Q9HAU8	M	Cytosol	3	0	0
AOPEP	aminopeptidase O	ENSG00000148120	Q8N6M6	M	Nucleus, Cytosol	3	0	0
KDM8	lysine demethylase 8	ENSG00000155666	Q8N371	*	Nucleus, Nucleoplasm, Cytosol	3	0	0
ANPEP	alanyl aminopeptidase, membrane	ENSG00000166825	P15144	M	Extracellular, Cytoplasm	4	0	0
ENPEP	glutamyl aminopeptidase	ENSG00000138792	Q07075	M	Plasma membrane, Cytoplasm	4	0	0
DPP4	dipeptidyl peptidase 4	ENSG00000197635	P27487	S	Many different membranes	4	0	0
MMP14	matrix metallopeptidase 14	ENSG00000157227	P50281	M	Cytoplasm	4	0	0
NAALADL1	N-acetylated alpha-linked acidic dipeptidase like 1	ENSG00000168060	Q9UQQ1	M	Plasma membrane	4	0	0
AMZ1	archaelysin family metallopeptidase 1	ENSG00000174945	Q400G9	M	Cytosol, Nucleus	4	0	0
LVRN	laeverin	ENSG00000172901	Q6Q4G3	M	Cytoplasm, Membrane	4	0	0
CTSV	cathepsin V/Cathepsin L2	ENSG00000136943	O60911	C	Lysosome	4	0	0

C: cysteine peptidase; M: metallopeptidase; S: serine peptidase; * Jumonji oxygenase family

Supplementary table S2. List of the 39 annotated aminopeptidase genes in the human genome utilizing the Ensembl and NCBI databases and further confirming the molecular function (gene ontology) of the identified genes. (3/3)

Gene symbol	Gene name	ENSEMBL ID	UniProt ID	Protein/ peptidase family	Protein cellular compartment	Aminopeptidase gene expression-based clustering as indicated in Fig. 1 (Cluster no.)	MM poor prognosis indicators (both in FIMM & CoMMpass datasets)	Protein detected with LC-MS/MS in MM patient samples
XPNPEP2	X-prolyl aminopeptidase 2/Xaa-Pro aminopeptidase 2	ENSG00000122121	O43895	M	Extracellular, Plasma membrane, Cytoplasm	4	0	0
F11	coagulation factor XI	ENSG00000088926	P03951	S	Extracellular region or secreted, Plasma membrane	4	0	0
TRHDE	thyrotropin releasing hormone degrading enzyme	ENSG00000072657	Q9UKU6	M	Membrane, Cytoplasm	4	0	0

M: metallopeptidase; S: serine peptidase;

Supplementary Table S3. List of compounds used in the experiments.

Drug name	Mechanism/Targets	Approval status	Supplier	Supplier Reference	Label name	Solvent	Conc. range (nM)
Melflufen	Peptide-conjugated nitrogen mustard alkylating agent	Phase 3 clincal trial	Recipharm/ Oncopeptides			DMSO	0.1-100000
Melphalan	Nitrogen mustard alkylating agent	Approved	Sigma-Aldrich	M2011	Melphalan	DMSO	0.1-100000
Selinexor	XPO1/CRM1 inhibitor	Approved	Selleck	S7252	XPOVIO	DMSO	3-3000
Bortezomib	Proteasome inhibitor (26S subunit)	Approved	ChemieTek	CT-BZ001	Bortezomib	DMSO	0.1-1000
4-hydroperoxy-cyclo-phophosphamide	Alkylating agent	Approved	Santa Cruz Biotechnology	sc-206885		DMSO	0.1-10000
Tosedostat*	Aminopeptidase inhibitor	Phase 2 clincal trial	Cayman Chemical	23395		DMSO	
Bestatin*	Aminopeptidase inhibitor	Phase 3 clincal trial	Sigma-Aldrich	B8385	Ubenimex	DMSO	

* Tosedostat and bestatin were used in the viability assay.

Supplementary Table S4. Aminopeptidases used in the hydrolysis assay and their incubation buffers.

Enzyme	Provider/cat.nr	Control substrate	Incubation buffer; [S]:[E] ratio
DPP3	R&D Systems #8087	Arg-Arg-AMC	50 mM Tris, 150 mM NaCl, 0.02% (w/v) Brij-35, pH 9.0; 1000:1
XPNPEP1	R&D Systems #2970-ZN	Lys(Abz)-Pro-Pro-pNA	50 mM TrisHCl, 250 mM NaCl, 0.5 mM MnCl2, pH 8.0; 1000:1
LTA4H	R&D Systems #4008-ZN	Ala-AMC	50 mM TrisHCl, 150 mM NaCl, 10 mM CaCl2, pH 7.5; 50:1
LAP3	Origene #TP309052	Leu-AMC	50 mM Tris, 4 mM MgCl2, 1 mM MnCl2, pH 8.00; 100:1
DPP7	R&D Systems #3438-SE	Lys-Pro-AMC	5 mM MES in water, pH 6.0; 1000:1
RNPEP	R&D Systems #8089-ZN	H-Arg-AMC	50 mM Tris, 100 mM KCl, 1 mM DTT, pH 7.5; 50:1
ANPEP	R&D Systems #3815-ZN	Ala-AMC	50 mM Tris in dH20; pH 7; 1000:1

Aminopeptidases that hydrolysed melflufen in the *in vitro* assay are highlighted in green
* Hydrolyzes terminal ester
Cat. Nr: catalogue number; S: substrate; E: enzyme;

Supplementary Table S5. log2(RPKM) expression values for 39 aminopeptidases genes and 17 housekeeping genes in MM patient samples in the FIMM dataset (*n* = 122) (1/21)

	Aminopeptidese genes									
**Gene symbol**	*AMZ1*	*ANPEP*	*LVRN*	*BLMH*	*AOPEP*	*CTSH*	*CTSV*	*DNPEP*	*DPP3*	**Gene symbol**
**Sample_ID**	ENSG00000174945	ENSG00000166825	ENSG00000172901	ENSG00000108578	ENSG00000148120	ENSG00000103811	ENSG00000136943	ENSG00000123992	ENSG00000254986	ENSEMBL ID
**MM032_1**	-3.506306653	0.307412236	-1.826946714	0.842958268	-0.673242943	1.103550463	-2.075294876	0.468305635	1.34453481	
**MM051_1**	-2.914160783	-0.13637137	-4.882788911	0.547913796	0.156128284	2.062664107	-2.375420627	2.107968725	0.955867401	
**MM053**	-3.195376852	-2.191713492	-3.892487248	0.619972059	-1.054516352	0.99861052	-3.944521145	2.496171535	2.948862407	
**MM056_1**	-3.919011629	-2.79845111	1.081776968	1.425332524	-0.230671856	0.494367495	-3.661424541	0.16710151	2.626076074	
**MM063**	-3.799589483	-2.387844013	-5.253715334	1.650001921	-0.423380476	-1.350899403	-5.231400214	1.408026565	2.569586534	
**MM064**	-5.610572819	-5.933167474	-3.883568214	2.532869857	0.110536808	-2.543224081	-4.335641878	2.127915064	2.666980619	
**MM065**	-0.321244015	-0.816679226	-1.401826016	1.87124996	1.397257198	0.687769079	-2.133067606	1.628844758	2.602930185	
**MM066**	-4.340517577	-0.988860198	-3.625367559	1.381516703	0.194596581	0.435835921	-9.785925912	1.491964481	1.898515895	
**MM067**	-2.661007156	-5.789376563	-2.938635352	2.14389882	-0.140422222	-0.716103756	-3.937588849	1.127239802	2.191350865	
**MM070**	-1.481081989	-0.482798163	-3.723703735	2.467822817	-0.582166741	3.664201756	-4.977662976	0.602410557	2.464190809	
**MM074**	-2.744287597	1.468127166	-4.705889192	0.379947224	-1.280631392	0.835621624	-4.688565022	0.463520606	0.692850491	
**MM075**	-5.809964903	-3.045990303	-3.237119081	2.176747587	-0.974758236	-0.463473737	-6.978432598	0.838464451	2.81544268	
**MM078_1**	-2.74800672	-0.25840519	-4.70252525	1.820310496	-1.7099817	3.376316646	-5.945352198	1.109024472	2.927913947	
**MM085**	-4.585657725	-1.662544791	-3.724696964	0.896421763	-1.172142655	0.190175918	-6.396968836	-0.075923896	1.236833838	
**MM086**	-2.872044691	-4.651745912	-3.911745523	2.043308583	0.031340762	-1.213035834	-4.368863139	1.737499008	2.565862121	
**MM087**	-3.976489833	-9.990246392	-5.078790291	2.416899268	-0.906011693	-0.870323145	-4.581137161	1.270685436	2.46876798	
**MM088**	-5.518922878	-4.004661184	-4.450757087	1.902621962	-2.224646425	-1.644769454	-10.96433121	-0.473888282	1.62233143	
**MM089**	-2.936352388	-0.83131124	-1.666893955	1.533468418	-0.214929402	3.589966157	-4.814844678	-0.054254451	0.020177311	
**MM090**	-2.841172501	-3.241180932	-3.403883342	2.735234856	0.863122641	2.253797163	-4.367960947	1.072417257	1.657137461	
**MM093**	-3.799165462	0.451252628	-2.1011196	1.965637299	-0.771758825	0.768122462	-4.130012285	1.15971249	1.395665343	
**MM094**	-3.889684056	-2.190387901	-2.14052631	2.575357366	0.977028402	0.112086399	-4.874035811	1.429111961	2.568052246	
**MM096**	-4.528943032	-4.295144338	-5.776287188	3.257222647	-1.596963689	-1.010926355	-11.2093714	1.06244935	1.899852616	
**MM101**	-2.272201483	-2.382191159	-2.079961606	1.534256973	0.993937478	0.680512265	-2.005288477	0.758787881	2.051797148	
**MM114**	-3.198171408	-2.308846972	-5.650056303	2.182573608	1.004226702	-0.261458325	-3.639590459	1.523014986	2.047589401	
**MM115**	-5.224324131	-3.159047268	-4.453140077	2.506452479	-1.054102657	-0.9832046	-6.582269624	1.261283056	2.068351086	
**MM117_1**	-5.684917919	-4.122040962	-4.298950232	0.578004366	-1.992548649	-0.734589203	-2.0071578	0.353103917	1.743334317	
**MM118**	-2.43247643	-0.980005701	-1.370203279	1.879110405	0.557649954	1.259226305	-2.295605824	1.114478487	1.860816105	
**MM119**	0.115642229	-4.214477723	-3.238872397	1.113663632	0.321855922	-2.715125007	-6.415307659	0.913619445	2.400210414	
**MM121**	-3.802626738	0.833575352	-3.511167153	1.107660794	-0.109903256	1.713723467	-4.839484624	0.321255307	1.320720069	
**MM122**	-2.220329596	1.831655963	-3.819700183	1.451297988	-1.456458139	3.555343881	-3.322047054	0.007762567	1.570514624	
**MM123**	-3.597328639	-0.070141978	-3.165006518	1.752950173	-0.244488956	1.253299741	-2.964335126	1.280406928	1.347852019	
**MM126**	-3.384325396	-0.369538303	-3.740114199	1.431586604	-0.096423997	0.423485533	-10.20824535	1.025124729	1.432012765	
**MM128_1**	-3.46319861	-7.220350215	-2.662982263	1.842349695	-2.026963709	2.474552208	-6.781769866	0.557600245	1.599664212	
**MM131**	-5.744634839	-0.50041434	-2.982255904	0.943363117	-0.502777193	-1.151154081	-10.65952846	1.88681812	2.707026646	
**MM132**	-5.93488026	-3.501011558	-2.829209208	0.648271231	-1.968447622	-1.17492391	-5.931106115	-0.376472667	1.014705567	
**MM133**	-3.451539403	-1.256285753	-2.475965842	0.463157837	-0.282115077	1.637170097	-1.389646243	-0.602234043	1.208344506	
**MM134**	-3.768989156	-0.838101851	-2.949137323	0.950117114	-1.326382999	2.003681331	-5.31349319	0.235665816	1.601058707	
**MM136**	-4.551728516	-2.151857147	-5.22339462	1.391629917	-1.041006028	-1.049640608	-10.08329349	0.758837065	1.882626818	
**MM138**	-4.720041042	-1.493181298	-4.869760207	1.177902657	-0.601461273	0.555834297	-4.091086926	1.037250318	1.932645097	
**MM139**	-4.00145691	-0.813089645	-4.770147468	1.029703927	-2.036678571	0.406685065	-6.408009309	-0.233382689	0.915603809	
**MM140**	-3.393175903	-3.312337103	-3.052164209	1.121202974	0.686472287	-0.680709291	-4.701352468	0.721344293	1.256394704	
**MM026**	-4.337040947	-6.73540311	-4.257734599	1.379652376	0.318381006	2.028169511	-3.009757492	1.164101911	2.62401493	
**MM027**	-2.902040595	0.073880019	-3.461468004	1.543833511	-3.031067618	1.810917102	-10.85255812	0.813440815	2.994017334	
**MM028**	-2.719187373	0.157603454	-1.799251871	1.208933622	-0.821115091	1.983382514	-4.974601277	0.660621448	1.281481018	

Supplementary Table S5. log2(RPKM) expression values for 39 aminopeptidases genes and 17 housekeeping genes in MM patient samples in the FIMM dataset (*n* = 122) (2/21)

**Gene symbol**	***AMZ1***	***ANPEP***	***LVRN***	***BLMH***	***AOPEP***	***CTSH***	***CTSV***	***DNPEP***	***DPP3***	**Gene symbol**
**Sample_ID**	ENSG00000174945	ENSG00000166825	ENSG00000172901	ENSG00000108578	ENSG00000148120	ENSG00000103811	ENSG00000136943	ENSG00000123992	ENSG00000254986	ENSEMBL ID
**MM030_1**	-2.457772665	-1.016369399	-3.398184551	3.606842928	0.825692461	-0.341967447	-3.192982315	1.528509773	2.121567817	
**MM030_2**	-3.453352086	2.651257137	-6.543970739	2.266021927	-0.378381316	4.030384623	-4.500349241	0.932876626	2.454123401	
**MM031**	-5.648700614	-6.501809986	-3.161309527	0.857586426	-1.255282317	-2.428683777	-11.09410895	0.610166929	1.603482345	
**MM033_4**	-2.627882879	0.253747926	-0.942080868	2.097575765	1.297919867	1.107825529	-4.248224841	1.400080164	2.036395517	
**MM033_1**	-4.228667625	-2.190452659	-4.863057092	2.043328036	0.996040881	1.261764603	-5.83933515	3.085267203	3.177529628	
**MM033_2**	-5.429396951	-2.080245339	-5.906483951	2.244371989	0.022843346	-0.099582805	-3.881583819	1.232244069	1.981298732	
**MM034_2**	-1.556902746	1.11886743	-3.836479214	0.576564827	-0.566383021	4.778401642	-5.51265554	0.512247546	1.908233745	
**MM034_1**	-3.488281566	2.150846826	-4.573362035	1.739327759	-1.947306081	2.59827215	-7.168155154	0.76085226	2.529964464	
**MM037**	-5.885803055	-2.468156984	-3.45001139	1.176391608	-1.191518279	-0.902901337	-10.3532377	1.332026651	1.858207939	
**MM041**	-1.592908107	-4.219755964	-4.833308613	2.707736034	-0.636915833	-1.002881559	-4.427285959	1.909155756	2.9220473	
**MM042**	-1.201941693	1.543736039	-2.822017756	2.499938478	-0.906959562	1.070963443	-4.951147303	3.272073424	3.356238706	
**MM043_1**	-2.855574265	-2.975189637	-4.10129525	1.928039673	0.890177824	0.090479486	-4.414880477	1.480249494	1.737067646	
**MM043_2**	-4.013043899	-1.392484414	-6.113061249	2.656749462	1.385659746	1.302509187	-0.781030024	2.642356483	2.852409235	
**MM044**	-3.327014765	-0.666831341	-3.416802611	1.857044888	-0.616010791	-0.007187986	-3.372055167	2.257919034	3.215831637	
**MM045**	-1.32251248	1.695619792	-1.619300433	2.723368526	-0.496706384	2.327986022	-3.880257892	1.235427294	1.726976735	
**MM046_1**	0.237871261	1.977849078	-0.677149182	1.925347426	0.253138018	0.309828274	-1.423183252	1.470086893	2.856491599	
**MM046_2**	2.64890619	1.596061986	-2.486498843	1.883712688	0.387431452	0.653195743	-2.44325466	1.697932146	2.97480913	
**MM048_2**	-5.076462798	-10.81784709	-5.241575178	2.439312882	-2.099285039	-0.716261145	-4.933399847	-0.044521385	1.788990335	
**MM050**	-3.267319232	-0.380729455	-1.544554734	1.492619293	0.225490533	1.487067775	-1.35851619	-0.091134148	1.089244227	
**MM051_2**	-2.789104112	2.372308246	-3.768012377	1.80812473	0.48362149	1.533796649	-2.000270084	1.168608967	1.407503922	
**MM054**	-4.889199279	-1.10224155	-5.696236148	0.024959178	-1.768395561	-0.009696721	-5.198583019	2.034624802	2.169492634	
**MM056_2**	-5.018547324	-3.66189953	-2.180887954	1.980153024	-0.102837631	-0.288796737	-3.78951115	1.374314054	2.42912743	
**MM057_4**	-2.793624964	-3.713460449	-2.559872346	3.76640483	1.666472538	-0.818074895	-5.983831318	2.205064557	3.897810958	
**MM057_5**	-4.633626323	-2.723958921	-3.490189637	2.644675096	-0.347938082	-0.503617914	-10.32345339	2.021774059	4.014653633	
**MM057_6**	-6.144246724	-4.634964138	-1.833985941	2.336146953	-0.410499118	-1.776058278	-4.869315784	2.018752065	4.190837386	
**MM057_1**	-2.081498813	-1.702523601	-3.945311443	3.716193446	1.52994328	-1.283555409	-10.04757116	2.427104459	3.743314007	
**MM057_2**	-1.665299081	3.806613873	-1.973015856	2.403646111	-0.257950604	3.448478567	-3.847476578	1.489677251	2.980193975	
**MM057_3**	-5.166596609	-6.788035745	-2.841814226	3.274753359	-0.195589577	-1.371413288	-11.12879594	1.886809752	4.176800726	
**MM058_1**	-3.673295428	-1.169499633	-2.645590276	2.845462563	-0.408206861	1.148489733	-0.373869926	2.008667332	3.425941495	
**MM059**	-4.820747095	-4.152740448	-6.920764446	1.823659458	-0.61497843	0.848515965	-0.977244425	0.783378215	1.998655609	
**MM060**	-1.171574662	-1.608664107	-3.729261517	2.181131885	-1.159576477	0.132667839	-5.841232943	1.770419928	2.136324137	
**MM061_1**	-4.085275083	-3.223276687	-2.673548266	0.61968958	-1.449043562	-0.604891718	-2.451529579	0.966806293	1.828465839	
**MM068_1**	-1.736198503	-1.797888191	-3.859093559	1.176671583	-1.156378029	1.019665848	-5.534633144	0.86850949	1.7074967	
**MM068_2**	-5.467144958	-2.101925777	-5.109985489	1.414035586	-1.384733052	1.849711756	-11.71036444	0.859730737	2.093931211	
**MM091**	-4.391791773	-3.057274141	-6.538993954	2.022436583	-0.629206374	-1.038313902	-4.662829201	1.09271454	2.356885238	
**MM095**	-1.655301928	-1.40858603	-4.344066774	2.055045742	-0.227630674	0.050542527	-7.546853363	1.449307534	2.387807375	
**MM002_1**	-4.640271064	-2.267913187	-1.894060601	1.00234157	-0.845789485	-0.626907573	-2.258635764	0.318606887	2.407985425	
**MM002_2**	-5.007795199	-1.215849756	-4.055724039	0.695084953	-1.446172684	-1.429394332	-1.311759861	0.229352609	2.503540915	
**MM002_3**	-3.539082215	-1.957902506	-4.078044454	0.331010608	-0.788511901	-1.683439358	-2.548582586	-0.090183056	2.137663911	
**MM003**	-3.132931423	-2.388391196	-3.37897053	3.662687767	1.094968419	0.825355839	0.270756971	1.2702669	2.517658713	
**MM005**	-0.010921177	-3.995352711	-2.80964125	2.727480725	-1.503366233	2.947741682	-3.00954993	0.851629236	3.10721542	
**MM007**	-3.400797361	-1.51439745	-5.087025759	1.668685534	-0.41189335	0.714858502	-4.589372629	1.043443271	2.204490095	
**MM010**	-8.220066943	-3.853362437	-3.779304135	2.800977587	0.341228291	0.765129143	-3.801805211	1.812513383	3.662839986	
**MM011**	-2.713080946	-4.00196411	-4.004930207	3.021224175	0.447755321	-3.130185169	-5.782284125	1.802164015	3.521012097	

Supplementary Table S5. log2(RPKM) expression values for 39 aminopeptidases genes and 17 housekeeping genes in MM patient samples in the FIMM dataset (*n* = 122) (3/21)

**Gene symbol**	***AMZ1***	***ANPEP***	***LVRN***	***BLMH***	***AOPEP***	***CTSH***	***CTSV***	***DNPEP***	***DPP3***	**Gene symbol**
**Sample_ID**	ENSG00000174945	ENSG00000166825	ENSG00000172901	ENSG00000108578	ENSG00000148120	ENSG00000103811	ENSG00000136943	ENSG00000123992	ENSG00000254986	ENSEMBL ID
**MM014_4**	-3.520731115	0.125056677	-3.798786555	3.263391523	-0.361042088	1.466412297	0.496720856	1.494986768	2.491445922	
**MM014_2**	-3.630625123	-3.301811538	-1.877272731	2.191817455	-0.977620087	-1.08754328	-2.15352954	1.036313171	2.061679241	
**MM017_1**	-6.234500811	-6.109636489	-4.859738579	1.320133351	-0.292230515	-0.704977365	-3.301056016	0.971098892	2.519362861	
**MM017_2**	-5.943073229	-2.339014033	-2.833004627	2.225281128	-0.385268114	0.185364604	-2.789466518	1.053795647	3.144396212	
**MM017_3**	-4.665769093	-3.565252127	-5.557316399	3.11225745	-1.394212239	-0.592904591	-1.256436233	1.11976234	3.089409266	
**MM018**	-3.804876246	0.334889423	-1.390126996	1.757365706	1.810633236	0.309225408	-0.961350671	2.005586293	1.837891169	
**MM019**	-3.66114104	-5.826054289	-3.35654238	1.506092709	-0.629337557	-1.024749979	-1.424428488	1.187866255	3.505983803	
**MM020**	-0.621181484	2.774741819	-2.686358215	2.309097194	0.591274806	2.009894035	-2.920462389	1.276834572	2.716441067	
**MM022_1**	-2.558121529	-0.986515487	-3.77117016	0.806521076	1.410203009	0.473014096	-2.842882676	3.549524157	2.745016293	
**MM022_3**	-4.775939597	-6.093811842	-2.492944266	-0.328875603	0.599965296	-0.646849956	-10.12971746	1.321874544	1.81386834	
**MM024**	-1.624396894	-2.342372089	0.865709003	2.373807927	1.808996472	1.128353381	0.477256332	1.550375081	3.508422499	
**MM025_1**	-5.443130982	-3.675367893	-3.221530751	1.52311246	-0.080304909	0.410905812	-5.365742514	1.369967403	3.211551853	
**MM036_1**	-11.29685702	-10.77145476	-2.64390348	1.87852254	-1.249759127	-3.751373462	-5.105187693	1.117152417	1.434346088	
**MM036_2**	-6.553136863	-6.027734611	-2.081513092	1.700029289	-1.065110976	-3.095116151	-4.132902889	1.006112134	1.166734468	
**MM038**	-3.860118703	-1.945492644	-2.886959759	2.334299184	-1.357718116	0.940545421	-5.048693971	1.043515813	1.073202184	
**MM039**	-2.288255301	-1.129864106	-6.18188994	2.807503471	0.617899648	0.417290808	-4.703865617	1.442701597	2.802745678	
**MM049**	-3.711542345	-5.256529421	-1.684703416	1.576945078	0.577485952	-0.780462364	-4.356975288	1.084020062	1.71689492	
**MM071**	-3.485901881	-3.079307059	-4.913855676	2.184886784	0.120410825	-0.562501928	-4.552085975	1.109241776	2.524749692	
**MM080**	-6.113279099	0.382446964	-3.610761319	1.619418664	1.057469552	-2.814053273	-10.58071374	1.367802467	2.753849078	
**MM081**	-2.723805524	-2.919687244	-7.727239606	2.285003428	0.989394547	-0.135957005	0.453018031	2.108004965	1.762193736	
**MM082**	-6.430158555	-8.092383306	-3.658555661	1.753410772	-1.307224807	-2.159637224	-8.04082608	0.680094002	1.767687732	
**MM098**	-3.591155418	-1.47286375	-2.808660487	1.754065126	0.818756892	1.408399256	-1.059615065	1.856228235	3.467949983	
**MM102**	-2.653934114	2.436604188	-2.902416512	1.3527241	0.816489568	4.284549322	-6.315574348	0.466850191	1.362074903	
**MM109**	-4.041912759	-0.743749366	-4.927840992	0.933766364	-0.328184597	1.587630831	-3.161439805	1.565625709	3.240966075	
**MM111_1**	-5.76072136	-4.601447007	-5.181844002	1.838778338	0.427950463	-0.819017642	-0.763293419	1.74916236	2.866570659	
**MM111_2**	-2.463359112	-3.684770241	-4.130866144	2.457979054	1.78067491	-0.712422705	0.435528461	2.466057716	2.734507007	
**MM113_2**	-2.480981989	2.45861784	-7.148319149	2.729996661	-0.606261882	2.705556693	-4.736395893	0.915201333	3.030224954	
**MM117_2**	-4.936492183	-5.292445435	-4.516148917	1.41386643	-0.609436462	-0.795321667	-2.383780251	1.370379172	2.838853603	
**MM124**	-6.80593167	-6.746192991	-3.429788208	1.716296091	-0.530870975	-2.365925949	-11.08695319	0.999692363	1.879606288	
**MM129**	-3.60592906	-5.204854943	-3.155255684	3.237420918	0.695502925	0.843254872	-0.108903597	2.064737255	3.003442374	
**MM135**	-3.005417216	-4.310828859	-6.600199258	4.314405739	-1.046032375	3.423198081	-7.020083969	1.735125493	2.70134986	
**MM137**	-5.866753339	-1.9778565	-4.670263451	0.795104893	-1.667852279	1.000403818	-3.844556124	0.321746338	2.423299135	
**MM006**	-4.417870066	0.055767662	-4.793933311	0.362596957	-1.774706285	-1.793929525	-5.06330301	1.085284126	1.345139348	
**MM008**	-0.955058716	1.733851623	-4.384201974	1.710508201	0.667615943	2.337242548	-2.712654941	1.939285179	2.150028301	

Supplementary Table S5. log2(RPKM) expression values for 39 aminopeptidases genes and 17 housekeeping genes in MM patient samples in the FIMM dataset (*n* = 122) (4/21)

	Aminopeptidese genes									
**Gene symbol**	***DPP4***	***DPP7***	***DPP8***	***DPP9***	***ENPEP***	***ERAP1***	***ERAP2***	***F11***	***JMJD7***	**Gene symbol**
**Sample_ID**	ENSG00000197635	ENSG00000176978	ENSG00000074603	ENSG00000142002	ENSG00000138792	ENSG00000164307	ENSG00000164308	ENSG00000088926	ENSG00000243789	ENSEMBL ID
**MM032_1**	-0.345841867	4.658874835	2.073701507	0.98362785	0.316821745	3.64610416	5.661564841	-3.104151463	2.158472978	
**MM051_1**	-3.958530548	2.246319877	1.093776756	1.81111536	-2.342778814	3.198410456	3.502090059	-3.497396252	2.933521986	
**MM053**	-3.376723131	5.699727029	0.554206821	1.847065192	-5.169518999	3.466637346	5.25469558	-9.57056255	3.662422601	
**MM056_1**	-1.984750723	4.901526998	3.354337055	1.323364588	-1.588905255	4.631687804	5.851711891	-1.28144403	2.223855745	
**MM063**	-6.593351233	4.743562257	2.361861101	0.023663474	-2.147707952	4.748996524	5.158454346	-10.10646917	1.8869719	
**MM064**	-5.002618448	3.905095009	2.199161227	1.4380239	-8.086724677	3.884290794	5.204025901	-10.04298339	1.138717895	
**MM065**	-0.628607098	4.004203701	2.749766657	2.191982988	-0.857140459	4.29322544	2.456762714	-1.000582933	1.965014503	
**MM066**	-2.143078146	4.33972971	2.590662497	1.544592335	-2.190679666	4.471100458	4.986587669	-5.603003143	1.608636839	
**MM067**	-6.295344764	5.115266111	1.49197505	0.872192683	-4.361199288	3.548292953	5.132951947	-7.791389185	1.818434396	
**MM070**	-3.184388787	3.423768044	2.4516495	0.78053485	-2.309535591	4.638947591	4.683668011	-10.10988977	1.796428106	
**MM074**	1.170030017	3.855198792	1.890855308	0.802723077	-5.492811725	3.91520709	4.953939168	-4.818011519	1.581219594	
**MM075**	-2.166235448	4.178431228	2.814228677	0.866253142	0.263954921	4.553403115	5.990255757	-10.58341239	0.956848863	
**MM078_1**	-4.175597537	3.623036511	2.343249403	1.936969759	-4.633208008	4.669077818	4.594110298	-7.65049615	0.218785862	
**MM085**	-4.100560101	4.357869554	2.091078774	0.349609906	-3.715769032	3.464084187	3.366505269	-5.358092437	1.074222972	
**MM086**	-3.012672438	4.211965362	2.810840289	2.120971025	-2.92814373	3.970384221	6.386172402	-4.498309636	2.214347843	
**MM087**	-2.699634142	3.988255377	2.414229398	1.869008289	-4.836982393	4.513111167	5.370042769	-9.456206115	1.592331215	
**MM088**	-1.159860664	3.716339645	1.827013697	0.589618389	-4.776651217	3.675856693	4.040663234	-10.48184816	0.575959529	
**MM089**	-2.137319461	3.505488649	1.947851027	0.409562559	-1.378533744	4.181492102	4.445638886	-4.332361627	2.271778317	
**MM090**	-2.287088267	3.675186286	2.528478273	0.642087937	-3.83400212	5.28822485	5.34030656	-5.074511721	2.260506487	
**MM093**	-2.822377492	4.308515567	2.345045237	1.465712742	0.264156438	4.48492739	5.936855842	-4.747064908	1.246314537	
**MM094**	-3.404586433	4.86464691	3.060597874	1.984256947	-2.710105625	4.878618183	4.04004847	-5.661641923	3.062777365	
**MM096**	-4.481054289	4.12570487	1.559694982	1.429153566	-4.974770353	3.530626224	4.442537815	-6.639425504	1.314072705	
**MM101**	-2.334026999	4.673118203	2.727876608	1.464687076	-0.834139405	4.265806367	4.275338129	-2.611975935	2.473921863	
**MM114**	-5.957960844	3.722490733	2.189734685	0.716977452	-2.430566785	4.216505619	4.744090873	-10.02747213	2.205768752	
**MM115**	0.244731758	4.298829337	2.17060635	0.949889764	-4.221006617	3.85933054	5.997944737	-7.017324413	1.917714941	
**MM117_1**	-3.934678227	2.663588946	1.433740549	-0.103241358	-5.013820214	3.762892766	5.964816204	-10.4583654	0.58551431	
**MM118**	-1.39206958	3.147091199	2.37801051	1.299832047	0.82643108	3.758049985	2.357262727	-2.449532039	2.123962304	
**MM119**	-6.507169513	4.49753766	1.767603763	0.616959326	-3.641496704	3.996973022	6.094152699	-7.698359354	1.578305185	
**MM121**	0.190422485	3.593921677	1.791348691	0.80177472	-1.442634476	3.648537566	4.859886142	-5.700955974	1.978109059	
**MM122**	-0.896675498	3.841070703	1.735596308	0.282076292	-1.305172557	3.109794051	5.156656462	-5.014338404	1.331820362	
**MM123**	-1.857553163	3.844858795	1.879967673	1.162583176	-2.234838203	3.888846309	4.410517384	-9.196097593	2.886332996	
**MM126**	-0.465636159	4.031676324	1.964828582	0.602403095	-0.723895596	4.01954304	2.865728676	-9.725762303	1.817802201	
**MM128_1**	-6.873631721	2.417758145	1.778008221	0.350886711	-4.942204466	3.91026546	5.234482179	-4.278225199	1.453950041	
**MM131**	-1.202568404	3.428514395	2.393585685	1.580092866	-2.21828419	4.63286983	2.963037036	-10.17704541	1.122658634	
**MM132**	-3.054090488	3.27892171	1.958067652	-0.176268402	-0.259537236	3.049723286	4.452623827	-3.750892633	1.012880414	
**MM133**	-2.809614767	2.727016135	1.081053435	0.366996081	-0.800381836	3.239342002	4.115537605	-3.909631447	1.542929108	
**MM134**	-3.516776328	3.783518675	1.288584042	0.102101774	-3.691887373	2.621759408	2.583710266	-6.870538503	0.871616195	
**MM136**	-0.641825617	3.545645618	2.146181448	0.950459897	-3.848692452	4.574170412	4.800016838	-6.430885442	2.056898181	
**MM138**	-4.288882226	4.3908613	1.589677005	0.44930307	-1.954198821	4.514226906	5.962619962	-7.258857836	1.830809369	
**MM139**	-1.10028574	3.569169412	2.010876321	0.438298916	-3.261745733	2.627295944	1.744193812	-6.882457536	0.84093407	
**MM140**	-0.954653533	3.241281718	1.616604723	0.315162389	-5.21435554	3.962939508	4.980916994	-9.833579261	1.935555796	
**MM026**	-1.752034909	4.874659242	1.688262068	0.407241574	-1.08780673	5.468715872	5.999351211	-10.28882567	1.604605157	
**MM027**	-3.237060846	4.297837069	1.741973829	1.013914855	-11.58374136	3.701555407	4.245814862	-10.37007507	0.577348006	
**MM028**	0.265002265	3.763858683	2.347342317	0.589239178	2.270677319	4.332919961	5.130395278	-6.936903068	0.904020389	

Supplementary Table S5. log2(RPKM) expression values for 39 aminopeptidases genes and 17 housekeeping genes in MM patient samples in the FIMM dataset (*n* = 122) (5/21)

**Gene symbol**	***DPP4***	***DPP7***	***DPP8***	***DPP9***	***ENPEP***	***ERAP1***	***ERAP2***	***F11***	***JMJD7***	**Gene symbol**
**Sample_ID**	ENSG00000197635	ENSG00000176978	ENSG00000074603	ENSG00000142002	ENSG00000138792	ENSG00000164307	ENSG00000164308	ENSG00000088926	ENSG00000243789	ENSEMBL ID
**MM030_1**	-1.689115238	3.804017395	2.080136572	1.646539109	-1.641675773	4.351204483	4.108653369	-6.8643046	2.334048616	
**MM030_2**	-2.994133095	3.429550899	1.854993094	1.370334359	-2.142907583	3.80528993	4.279152976	-10.20769075	1.670122121	
**MM031_2**	-0.530440081	3.371308655	2.017795746	-0.279836775	-2.223521402	4.095525053	5.705414749	-7.441700896	1.901512304	
**MM033_4**	-5.11060485	3.602662504	2.777393436	1.794118074	0.338784072	3.309616354	2.237813426	-4.536259944	2.545201777	
**MM033_1**	-9.101122007	5.696102245	2.495458384	3.097784539	-1.690594844	4.186549497	2.142565824	-8.526777101	3.620055341	
**MM033_2**	-4.730174523	4.155991149	2.88776793	1.398906732	-1.496157874	3.718312404	0.740134375	-9.013810612	2.213793733	
**MM034_2**	-1.779675046	3.527756703	2.227828599	-0.170771834	-1.669176034	3.724207937	4.019497216	-10.38772449	1.276068252	
**MM034_1**	-0.575165277	4.748463451	2.381181938	1.019277988	-1.919366876	4.011897116	4.349329107	-10.77313494	1.135746583	
**MM037**	-1.008388009	4.499503117	1.673462109	-0.273219283	-4.37017542	2.909748165	5.151048535	-9.870754645	1.154342769	
**MM041**	-2.357128011	2.873047845	2.307322277	1.342147046	-1.738267357	4.245060145	3.71398936	-2.344158785	1.703869456	
**MM042**	-2.94064744	5.086507309	1.990792678	3.470068787	-3.503006845	4.248824383	3.147144487	-4.855687375	2.464236289	
**MM043_1**	-2.800034558	4.043958146	2.885410008	1.39150873	-0.310787901	4.26082636	4.808067351	-5.01646169	1.67030914	
**MM043_2**	-2.575564296	3.820568478	2.523281393	2.05394882	-0.690902809	4.274505111	4.583654235	-9.22038791	2.336314487	
**MM044**	-3.684605136	4.329384437	1.793824369	2.205478919	-1.568597492	3.364513155	3.522569836	-5.636806045	2.638837277	
**MM045**	-0.673870649	4.211324416	2.835054378	1.348136175	-0.952897501	4.314588967	4.737098021	-2.674292476	2.46060896	
**MM046_1**	0.426117069	3.978087527	2.384024371	1.708989605	-0.158394496	4.345380376	3.66209073	-0.632784197	2.041017393	
**MM046_2**	-1.776256566	3.758739378	2.218185675	2.098996619	-1.116822013	3.891531085	3.34992014	-1.870749738	2.108699969	
**MM048_2**	-1.878012138	3.870504665	2.028049552	1.676612172	-3.790113971	4.016724193	4.102192617	-7.113881808	1.28591492	
**MM050**	-2.864098616	3.63674115	1.869291967	0.993802371	-3.54616965	4.276838199	5.56600154	-4.178332427	1.544596019	
**MM051_2**	-2.179155001	2.129348015	2.13620756	0.862502194	0.535241815	4.311108626	4.310863964	-6.027342131	1.769970095	
**MM054**	-4.720129149	4.517136362	1.249808748	1.529760907	-10.97416038	2.878549452	3.239424001	-4.268640991	1.860160943	
**MM056_2**	-10.80023624	6.032833623	2.378409058	1.654516132	-11.43955763	4.609822754	5.344320077	-2.265889404	2.416108597	
**MM057_4**	-7.454204796	4.654581389	2.486500343	1.416296275	-2.455452345	4.999114362	4.260961203	-6.87985989	2.396804649	
**MM057_5**	-4.075465238	4.756767336	1.65271697	0.492627244	-2.76461778	4.847979211	2.730463627	-9.840970335	1.556901247	
**MM057_6**	-4.961177639	4.741936446	2.175235275	0.210651082	-2.59803077	4.784213699	2.258366031	-10.57665729	1.656583588	
**MM057_1**	-4.117065197	4.909848346	2.803952098	1.967213285	-2.728905848	5.209346236	4.211073224	-9.565088104	2.649623437	
**MM057_2**	-0.937743414	4.185085273	2.329053202	1.467911233	3.480983748	4.368180262	3.64926902	-10.18517249	1.351777927	
**MM057_3**	-5.387767782	4.305408745	2.471040929	0.464703991	-1.892752924	4.942214444	3.161746761	-10.64631289	0.722058468	
**MM058_1**	-2.161158552	1.876191124	2.616820491	1.205208141	0.377924047	4.034494269	4.984397741	-2.814317481	0.688061707	
**MM059**	-4.493911556	3.851301097	1.793281592	1.454547035	-3.679514975	3.351628919	5.672150026	-3.761304566	1.178829084	
**MM060**	-4.45122579	4.443757307	1.57973745	0.898497478	-0.986417637	4.470830362	4.724058173	-7.046805885	1.346495864	
**MM061_1**	-2.670303547	3.516615081	0.957474767	0.671339921	-2.748816294	2.985063149	3.8823857	-1.462086539	1.023510681	
**MM068_1**	-2.194080931	3.675499764	2.294998047	0.740500992	-1.640927701	3.799577692	6.45652497	-10.66685994	1.292360584	
**MM068_2**	-0.568007619	3.782166845	2.446404821	-0.346082778	-0.671296757	3.431440778	5.811062825	-6.183487272	0.726383539	
**MM091**	-6.689596028	4.765208798	2.497264881	1.953327948	-0.484904022	4.215097834	4.884943983	-5.558857773	1.805148166	
**MM095**	-1.656355377	3.862242875	2.657247808	1.301937664	-2.00294676	4.47422522	4.387001387	-4.303557975	2.043262197	
**MM002_1**	-1.611388347	5.326149815	1.84699646	-0.247072531	-1.071177404	3.558230127	3.51935412	-2.83638782	1.81617767	
**MM002_2**	-4.189571275	5.209150922	1.923715678	0.351590105	-2.403665941	3.789838207	3.240410132	-5.822821788	1.595715236	
**MM002_3**	-2.604930154	4.887655445	1.907688855	-0.005705793	-0.950664591	3.661540045	3.540023967	-10.39252902	1.758640725	
**MM003**	-2.684709802	2.400375963	2.676349145	2.535752856	0.399212778	4.629655521	3.832340208	-3.972436988	2.654157806	
**MM005**	-2.403849975	3.243646423	2.602852412	1.22375546	-3.39655227	3.81150963	1.263632339	-4.188786578	2.084789778	
**MM007**	-5.52923139	3.737389736	2.858135569	0.958731148	-3.077699347	4.339157703	4.771657574	-9.598742674	1.724215517	
**MM010**	-6.261038131	4.554939615	2.345517207	1.626940533	-3.951758671	4.323569842	5.445809988	-10.33054942	1.363284917	
**MM011**	-5.160450165	4.351200628	2.718397541	1.679426394	-4.890537016	4.06961898	4.362344582	-9.943657264	1.934155606	

Supplementary Table S5. log2(RPKM) expression values for 39 aminopeptidases genes and 17 housekeeping genes in MM patient samples in the FIMM dataset (*n* = 122) (6/21)

**Gene symbol**	***DPP4***	***DPP7***	***DPP8***	***DPP9***	***ENPEP***	***ERAP1***	***ERAP2***	***F11***	***JMJD7***	**Gene symbol**
**Sample_ID**	ENSG00000197635	ENSG00000176978	ENSG00000074603	ENSG00000142002	ENSG00000138792	ENSG00000164307	ENSG00000164308	ENSG00000088926	ENSG00000243789	ENSEMBL ID
**MM014_4**	-2.381344921	4.247705836	3.025418264	2.277020471	-0.521354748	4.028053896	4.740927431	-4.62211138	1.325852716	
**MM014_2**	-3.569783529	4.154897506	2.606881729	1.352862876	-0.124546344	3.802203619	5.145267123	-2.464923907	1.223989396	
**MM017_1**	-5.436245302	4.420959932	2.245519052	0.636154358	-0.770450318	4.895082447	1.805864929	-7.049527399	1.840086871	
**MM017_2**	-1.868873375	4.227223705	2.482366943	1.194760584	-0.071871001	4.936007423	3.430278808	-2.97342737	1.89459815	
**MM017_3**	-5.151525124	4.009473175	2.946616994	1.674294298	0.602157746	4.842910494	-0.821570088	-9.221036408	0.944676216	
**MM018**	-0.396014208	3.920833947	2.284446564	1.625870393	0.502644239	3.957970026	3.236022739	-0.658969626	3.028465791	
**MM019**	-3.691584517	5.690414763	2.311201177	0.810020477	-2.961359788	3.938836965	1.825884986	-3.927017195	2.570871648	
**MM020**	-0.496127328	4.760214793	3.958198595	1.186865631	-2.274467675	3.975012267	4.334070467	-4.477507702	2.565147613	
**MM022_1**	-0.946426336	6.481668905	2.517636443	2.504363922	-0.186928734	4.518392899	3.85599496	-8.899558436	2.986084548	
**MM022_3**	-4.606869468	4.491132379	1.876465952	0.789238524	-0.201796735	4.300905321	4.478982685	-9.647234406	1.675723785	
**MM024**	0.996072016	4.404914107	2.951999744	1.345681031	1.489720766	5.283442959	3.328943973	0.493730978	2.278667638	
**MM025_1**	-2.426842115	4.143622983	2.652387176	1.464647592	-2.210072913	4.406806375	5.251736053	-5.653777617	2.412093062	
**MM036_1**	-7.111319674	4.049544323	1.714940055	0.364254137	-6.40668666	4.022717556	5.036185325	-10.23741449	0.303673286	
**MM036_2**	-6.06803924	4.196519743	1.57456663	0.802178122	-7.624898466	3.915216673	4.855732644	-6.411232173	0.038715695	
**MM038**	-0.357037543	5.171636932	2.071835914	0.045132181	-2.069030271	4.639701275	4.725376004	-10.97560186	0.951098805	
**MM039**	-1.574041043	4.924634022	2.405363931	1.158402397	-1.283910653	4.585554245	4.972025633	-4.001816189	1.89191158	
**MM049**	-3.282478757	4.156825402	2.446068952	0.751246599	-3.112406076	4.139301923	5.103769644	-6.196420332	2.959972987	
**MM071**	-4.064934595	4.120935856	1.755304951	1.578212136	-3.408800098	3.695008147	2.071998073	-5.199999561	2.223271678	
**MM080**	-3.415187752	4.546219228	1.901781591	0.847631088	-1.207298228	3.544590605	4.151818946	-5.454374499	2.06587328	
**MM081**	-3.723189008	2.2280984	1.222774667	1.801876956	0.399223808	3.113470395	2.691315426	-9.917028427	3.044354824	
**MM082**	-3.595253804	5.063584602	1.962250955	-0.22439407	-3.727615202	4.03135605	2.954115673	-10.72826803	1.764508363	
**MM098**	-4.297000577	3.752456164	2.963748236	1.843398953	-1.025806505	4.49477825	4.290675402	-3.260751567	1.939976759	
**MM102**	-1.055640595	3.500440582	2.750541719	0.743574874	-0.30804489	3.633922792	4.448855293	-11.44780114	0.701201584	
**MM109**	-3.704913774	3.613946007	1.042379249	1.012696057	-5.217405139	2.860198777	4.734502188	-5.452496963	1.294794524	
**MM111_1**	-4.776052727	3.019684573	2.081593159	0.635975495	-5.728531999	3.1331777	3.282548881	-3.369435267	1.173439798	
**MM111_2**	-3.20065391	1.865941186	2.472388272	2.172724655	-4.939510971	3.533399507	1.621430839	-4.948237099	1.903386246	
**MM113_2**	0.265254138	3.31085788	3.166863496	1.62699635	0.443721128	4.478025103	2.414569406	-3.154377169	0.996704929	
**MM117_2**	-3.182646503	3.233636071	2.291212645	1.209639897	-4.017058905	4.164006224	5.947166164	-4.758405157	1.029359859	
**MM124**	-5.821263037	5.010619479	2.053472122	0.227022399	-3.259715716	3.545728518	5.513615252	-10.60447014	1.056283757	
**MM129**	-3.41328233	2.255527346	1.584545364	1.776171857	-0.897160092	3.705864664	3.172258306	-9.314670855	1.823993825	
**MM135**	-2.048251148	3.003567724	2.404522982	1.637547575	-10.92119221	4.047355751	4.237021486	-9.707525919	1.947130997	
**MM137**	-3.444021597	2.208825244	2.03967748	0.642325857	-4.677022702	3.500218487	5.181540357	-5.785284504	0.981175824	
**MM006**	-2.938206329	4.939668235	1.18240001	0.109309793	-0.968272302	3.144537875	3.756585393	-3.233800883	1.315367517	
**MM008**	-1.302949333	3.725664723	2.109737488	1.461572382	-0.665460139	4.00340418	4.615862672	-6.23546619	1.95462848	

Supplementary Table S5. log2(RPKM) expression values for 39 aminopeptidases genes and 17 housekeeping genes in MM patient samples in the FIMM dataset (*n* = 122) (7/21)

				Aminopeptidese genes					
**Gene symbol**	***KDM8***	***LAP3***	***LNPEP***	***LTA4H***	***METAP1***	***METAP1D***	***METAP2***	***MMP14***	**Gene symbol**
**Sample_ID**	ENSG00000155666	ENSG00000002549	ENSG00000113441	ENSG00000111144	ENSG00000164024	ENSG00000172878	ENSG00000111142	ENSG00000157227	ENSEMBL ID
**MM032_1**	-1.289402851	3.853425761	2.293833014	3.355970894	1.925104264	-0.855697351	3.728663153	-0.662520596	
**MM051_1**	-0.407259744	3.951547759	2.088580522	2.245401923	1.969887121	0.327313121	2.03756467	-1.537271843	
**MM053**	-1.370858617	4.498398223	2.571615843	2.855620179	1.564810601	-0.915975238	2.731325343	-3.638871869	
**MM056_1**	-2.19147853	5.033232182	3.824190381	4.599557776	2.667472688	0.017070956	4.652336978	-3.227370684	
**MM063**	-1.811868895	4.965286696	2.822634779	3.957987342	2.085175067	0.741306303	4.137169176	-5.47023437	
**MM064**	-2.235341464	7.969959649	3.349448963	2.675821934	1.611410456	-0.541503201	4.644305848	-6.363679866	
**MM065**	-0.403543121	4.26986211	2.756871986	3.133009933	2.114267921	1.95138011	3.790045758	-1.310418421	
**MM066**	-1.728059513	5.150837212	3.25452889	4.079816563	2.810548818	1.117847253	4.137387596	-4.096892338	
**MM067**	-1.729679294	4.274329961	2.168453865	3.753160177	1.750401431	1.09739021	3.965741172	-4.793556147	
**MM070**	-2.198784526	3.442908872	2.915090208	3.979781978	2.423056622	0.789092084	4.326024534	-2.866997399	
**MM074**	-1.485571867	4.092966867	2.862845704	2.786765382	2.087650307	-0.605114972	3.531126988	-2.250187722	
**MM075**	-2.383708455	5.122149386	3.338888646	4.205297096	3.189662039	-1.167239515	5.148304479	-2.233348495	
**MM078_1**	-1.65588142	5.5418344	1.940249936	4.884568597	2.034890869	0.13609123	3.984495735	0.198208421	
**MM085**	-1.481134221	4.255575995	2.841507801	3.032954837	2.277677178	-1.387648611	4.012755252	-3.398880693	
**MM086**	-0.359479969	4.993050383	3.139104113	3.783554785	2.879627698	0.67693804	4.678451493	-2.795185624	
**MM087**	-1.606284389	5.318070083	3.27521859	4.050641369	2.559863591	0.488677683	4.590012162	-4.506813431	
**MM088**	-2.455649755	3.222345253	2.354323007	5.735409978	1.298533618	-1.400476465	4.310022526	-3.7919754	
**MM089**	-0.978798089	4.081688116	2.849302448	3.260238341	2.383377349	0.480220962	3.453274254	-2.490294319	
**MM090**	-0.713061672	5.765956735	3.885752643	5.290596132	3.027248451	1.783457376	4.877964872	-2.658921682	
**MM093**	-1.645455615	4.772211377	2.991701535	3.401920285	2.416427096	-0.748752023	4.438604522	-1.658798403	
**MM094**	-0.418600601	4.985084972	2.93576356	2.984124702	2.572578287	2.011089084	4.51818213	-3.557351244	
**MM096**	-2.142085845	5.374550275	3.025305939	4.017089437	2.2873302	0.749876228	4.844693246	-3.840426918	
**MM101**	-1.536544183	4.830888256	3.387295639	3.164197861	2.857043625	0.245601732	4.182514417	-1.743448751	
**MM114**	-1.098999753	4.613234917	3.095982804	3.417720758	2.495093681	0.931053084	3.358689995	-1.432816433	
**MM115**	-1.154153648	5.743974666	3.111373649	4.120235686	2.849589016	0.16835053	4.012497844	-2.888049604	
**MM117_1**	-2.785547905	5.171432979	1.920909729	2.759265362	1.711954075	-0.469487496	3.603608385	-7.696599724	
**MM118**	-0.68156599	5.107261799	2.927914548	3.310257778	2.878418738	1.702628687	4.153227195	-0.294315722	
**MM119**	-0.088120591	5.137886198	2.961800632	3.964789132	2.3350805	0.412631504	4.001190576	-3.417219515	
**MM121**	-1.9036649	4.225035367	3.057511026	3.512440246	2.413424327	0.425046648	3.942984848	-1.716797873	
**MM122**	-1.0628097	4.571495704	2.82599896	4.776077337	2.156903036	-2.028118852	4.510828284	-1.376779425	
**MM123**	-0.502815992	4.191848721	2.832577355	3.70190803	2.94528952	0.784880528	4.112942146	-3.194865978	
**MM126**	-0.219744899	4.836342537	3.328216786	3.581103916	2.146786046	0.451198895	3.485482418	-0.299450574	
**MM128_1**	-2.187045723	4.490301274	3.360366905	4.022082806	2.867402445	-1.138123665	4.089740265	-5.180199133	
**MM131**	-1.153153467	4.712732648	3.260442992	3.579606276	2.595776382	0.526968731	4.514007347	1.633055771	
**MM132**	-2.639542958	4.265729715	2.327220464	2.632609784	1.718792897	-1.570769744	3.404784753	-2.67130257	
**MM133**	-1.687319413	3.956614777	2.279819127	3.055269554	1.25163128	-0.67161314	3.255277611	-2.547613395	
**MM134**	-1.873831394	4.110573902	2.703991662	4.404936573	2.544829534	-0.517061606	4.266178447	1.146168292	
**MM136**	-1.028580529	4.848892728	3.250583091	3.42240162	2.09681345	-0.840905106	3.348789366	-3.469810954	
**MM138**	-1.735501236	4.127798701	2.702063075	3.425619188	2.324366302	0.343190524	3.551029816	-0.266137721	
**MM139**	-0.438512851	3.710395072	1.630032714	3.039704373	2.094703121	-0.378137798	3.52465941	-2.914555325	
**MM140**	-1.223955589	4.435421775	2.875989578	3.067191968	2.597172136	-0.056464633	2.996450739	-3.1437065	
**MM026**	-2.459888973	5.48972254	2.901864897	2.094989833	1.564735408	-0.457981514	3.418517416	-2.335041221	
**MM027**	-0.403875077	6.217160274	2.533698307	3.815985462	2.801650355	-0.902114963	4.917320818	-4.945344379	
**MM028**	-1.390508932	4.380430616	3.179373462	4.50076472	2.976388174	-0.063830674	4.566301243	1.268420151	

Supplementary Table S5. log2(RPKM) expression values for 39 aminopeptidases genes and 17 housekeeping genes in MM patient samples in the FIMM dataset (*n* = 122) (8/21)

**Gene symbol**	***KDM8***	***LAP3***	***LNPEP***	***LTA4H***	***METAP1***	***METAP1D***	***METAP2***	***MMP14***	**Gene symbol**
**Sample_ID**	ENSG00000155666	ENSG00000002549	ENSG00000113441	ENSG00000111144	ENSG00000164024	ENSG00000172878	ENSG00000111142	ENSG00000157227	ENSEMBL ID
**MM030_1**	-0.678563666	4.295712397	3.862803473	3.118351139	3.206442668	1.988565865	4.839657443	-3.254542014	
**MM030_2**	-2.237891512	4.652214125	3.300698604	5.437224823	1.909488621	0.827221637	4.073340878	-1.453458741	
**MM031_2**	-1.438734796	3.965454966	2.918210524	3.833418975	1.334467107	-0.400496258	4.133473604	-3.488403759	
**MM033_4**	-0.543247317	5.085840587	2.315252518	3.818210307	3.966166658	2.096990778	4.432743441	-0.982033557	
**MM033_1**	0.019974094	5.387666587	2.933314954	3.410567492	3.28673346	1.859053286	3.451094512	-2.016073185	
**MM033_2**	-1.082874198	5.454113681	2.011893867	3.796456399	3.592808121	1.956732954	4.413741919	-2.707724416	
**MM034_2**	-2.361526087	5.150624155	3.66384142	5.847380546	1.517154509	-2.662866103	4.091985413	-0.556285286	
**MM034_1**	-2.43332904	6.06645782	3.051140566	4.365956966	2.097997995	-0.467261237	4.897406722	-1.752934093	
**MM037**	-2.603193625	4.117502766	3.096642154	4.036348832	1.528264168	-0.437111992	3.87170584	-0.12283089	
**MM041**	-1.159221108	4.798314268	3.637642752	2.882303609	2.392660097	0.744493606	4.672347713	-5.891382144	
**MM042**	0.101893403	4.428100772	2.168878855	2.485291803	2.849859171	-0.510286947	3.355969252	-3.23636596	
**MM043_1**	-0.722014497	4.642066989	3.414739619	3.389301498	3.3680796	1.002317097	4.042169553	-2.446425382	
**MM043_2**	0.620390403	4.4456455	3.000085394	3.442903091	3.008078269	1.459429516	3.950179013	-2.530515149	
**MM044**	-1.1205164	4.606958099	1.863029151	2.241652876	2.514129347	0.032720637	5.010275662	-1.295175732	
**MM045**	-1.313072977	5.279567573	3.103963353	3.959192718	3.277666935	1.537150228	4.469369723	-1.114988733	
**MM046_1**	-0.985964157	4.787198304	3.723556574	4.339861714	2.770897694	0.319565181	4.510649452	-2.162934042	
**MM046_2**	-0.286118808	4.138008927	3.162020206	4.263982867	3.123096874	1.170178802	4.670650653	-1.478975626	
**MM048_2**	-6.155309828	4.864796918	2.71453496	3.292584276	2.613520815	-0.20335903	5.090827778	-3.680738876	
**MM050**	-1.077959046	5.492499976	3.679440389	2.694862385	2.63396494	-0.804457164	4.157618613	-3.9630551	
**MM051_2**	-0.887440385	5.012604758	3.278927661	3.202765504	3.192775175	0.259344285	3.889326944	-3.066267643	
**MM054**	-1.593861976	4.478847553	1.862068658	2.298827379	1.695024306	-0.876790141	2.391790473	-2.911265566	
**MM056_2**	-2.553073837	5.292489272	2.771673183	3.942777244	2.343973725	1.425277634	3.791421749	-4.444226099	
**MM057_4**	-0.734289394	5.96798121	2.877720294	3.80418909	3.585780959	2.679000086	4.648505436	-3.343160766	
**MM057_5**	-1.50116443	5.816281587	2.633884753	4.444727422	2.758828028	1.990148493	4.67110534	-4.059305097	
**MM057_6**	-1.261161794	5.488512148	2.950057753	4.280038845	2.425542302	2.038440454	5.005536789	-6.340960423	
**MM057_1**	-0.367429097	4.482829705	2.794238572	3.995431709	3.298472443	3.079688625	4.851783034	-3.864722969	
**MM057_2**	-1.106918262	4.525108558	2.667253108	4.364347418	2.4161443	0.634088126	4.692050716	2.096884469	
**MM057_3**	-0.84269897	5.930046472	2.770626662	4.326228484	3.233839128	2.713957362	5.359369254	-4.714622209	
**MM058_1**	-1.573004445	8.473779277	3.278433084	2.572787153	3.141368712	1.526937606	3.893298065	-2.90234199	
**MM059**	-1.588007974	5.30104749	2.119983008	2.964337164	1.8730942	0.003478942	4.602216401	-2.936404541	
**MM060**	-1.401066543	6.166197769	2.645462661	4.005433015	2.776822022	0.117324412	4.458547816	0.706586614	
**MM061_1**	-1.798970442	5.738087025	1.938501045	3.191317973	2.396295008	0.010996697	3.84736092	-3.873812476	
**MM068_1**	-0.884920716	4.15246332	2.702205039	3.202797112	2.001907301	-1.843727797	3.375008052	-3.047113262	
**MM068_2**	-1.072795101	5.06968816	3.22419068	4.259046247	2.037328661	-2.449626744	4.223334568	-2.567262435	
**MM091**	-1.877819768	6.427476342	3.426814025	3.447437136	2.536020119	0.961658572	4.823532448	-2.435947602	
**MM095**	-0.76849501	4.904816733	3.430518207	2.408079268	2.659319207	0.765863286	3.187360234	-1.172812817	
**MM002_1**	-1.339946047	4.431482472	2.702956867	3.379136644	2.092490392	-0.751540627	4.031079627	-2.961153648	
**MM002_2**	-1.378297312	4.183235767	2.454000528	3.218641864	1.802068491	-1.511725072	3.873379163	-5.008588688	
**MM002_3**	-1.758504006	4.146493059	2.513731263	3.039819208	1.089606582	-1.197528532	4.075974692	-4.086442826	
**MM003**	0.120052123	4.462294944	3.067873476	3.879867143	3.370552621	1.025898447	5.89006877	-2.171601743	
**MM005**	-1.547294001	5.602832156	3.165658875	4.48690462	2.175196432	0.589281242	4.879523849	0.302831969	
**MM007**	-1.399038741	5.587903171	3.884548518	4.278130726	3.10366452	-0.18844039	4.767447691	-4.649349991	
**MM010**	-0.359552424	6.166183101	3.444412105	3.574661608	3.213394464	1.268670586	5.230609289	-2.71080274	
**MM011**	-0.006579099	5.949137591	3.442137224	3.718768248	2.998696444	1.857491056	4.967632685	-4.994264581	

Supplementary Table S5. log2(RPKM) expression values for 39 aminopeptidases genes and 17 housekeeping genes in MM patient samples in the FIMM dataset (*n* = 122) (9/21)

**Gene symbol**	***KDM8***	***LAP3***	***LNPEP***	***LTA4H***	***METAP1***	***METAP1D***	***METAP2***	***MMP14***	**Gene symbol**
**Sample_ID**	ENSG00000155666	ENSG00000002549	ENSG00000113441	ENSG00000111144	ENSG00000164024	ENSG00000172878	ENSG00000111142	ENSG00000157227	ENSEMBL ID
**MM014_4**	-0.98267522	5.070234943	2.41301941	3.144510653	2.771254572	0.644252192	4.147727859	-1.76666931	
**MM014_2**	-1.195933129	5.458842914	2.661908047	2.708478025	2.312677063	0.134843726	4.151249033	-3.798229234	
**MM017_1**	-1.458133279	6.118861276	3.23081718	3.615018488	2.411158696	0.303269463	4.812611467	-6.540148881	
**MM017_2**	-0.602420112	5.289516014	3.138741475	3.262759083	2.304873165	0.665009304	4.701888846	-3.882984476	
**MM017_3**	-1.671075657	5.331282001	3.012698882	3.526858446	2.943131936	-0.026035919	5.04361753	-4.771214734	
**MM018**	0.610642134	4.90154721	3.009962822	3.445635585	2.266716426	1.008239315	3.936234824	-1.557359425	
**MM019**	-1.312516192	5.835081962	2.394455487	3.286993948	2.798047651	0.688934103	4.257304665	-4.911677438	
**MM020**	-1.029969204	4.782390365	3.567590039	3.628752521	3.297498003	1.647950553	4.234311869	4.780041737	
**MM022_1**	0.826725088	4.626967549	3.513684845	2.59881212	1.949216524	0.04227634	3.456927839	-0.98128827	
**MM022_3**	-0.482694674	5.004274523	3.136883094	2.359143964	0.962971009	0.040491552	4.025770806	-2.293842495	
**MM024**	-0.871054609	5.692345004	3.534028589	3.392949153	3.64298652	1.501718157	4.014438298	-2.26667689	
**MM025_1**	-0.84266957	5.244292316	3.277470744	2.92001327	2.87892304	0.442619474	4.130261972	-4.316509765	
**MM036_1**	-2.766525307	5.39702953	3.073774319	4.950256999	2.878039092	-1.063048843	4.618830008	1.415459794	
**MM036_2**	-3.369244184	5.372242444	3.080544209	4.302022907	2.601386448	-1.247877311	4.420956403	1.379735511	
**MM038**	-2.214282734	3.953342647	3.583542448	4.29519119	3.15563853	-1.235451813	3.90706914	-2.679857604	
**MM039**	-1.999070532	3.489217552	2.644714535	3.57158729	2.090370723	0.775660661	4.616075216	0.356183104	
**MM049**	-1.790961986	4.609232447	3.066119498	3.014997052	2.476986092	1.672377407	3.742928056	-3.060259137	
**MM071**	-2.259645438	4.34992107	1.602384903	2.138606833	2.264374503	1.015845182	4.435378997	-3.166560557	
**MM080**	-1.150162835	5.516211886	2.661340539	3.118013071	2.291811618	1.213559753	4.21139457	-5.862533821	
**MM081**	-0.391315222	4.690984456	2.567178577	2.121097181	2.359749484	1.595796732	4.430206624	-2.924308312	
**MM082**	-1.789964647	5.911899302	2.728802318	3.381263325	2.159136666	-0.139081394	4.777108904	-6.092033232	
**MM098**	-0.891029326	5.606811414	2.951668007	2.408588228	2.401710208	0.14185471	3.802446446	-2.236683628	
**MM102**	-1.520562366	4.326543692	3.767541975	5.602912985	2.452536361	-2.212395815	3.984671634	-1.204011851	
**MM109**	-2.089720414	5.495363918	2.033117502	2.716604718	2.719169766	0.264027685	4.710444088	-4.470196808	
**MM111_1**	-1.025418741	5.8389667	2.131823387	3.210357321	2.178956759	0.820574936	5.119813816	-1.408998434	
**MM111_2**	-0.2892254	5.583613795	2.232782208	2.568911038	2.488591631	1.813316091	5.101781499	-0.262884535	
**MM113_2**	-0.763936665	4.635729874	4.093664337	5.073113675	3.331438916	0.005516396	5.547630749	0.612721956	
**MM117_2**	-1.9816715	6.137296892	2.41618668	3.080268099	2.369510998	0.855670855	4.25647167	-6.355598303	
**MM124**	-0.717274038	5.711699379	2.754584251	4.239798724	2.955179906	0.909724129	4.255994576	-4.244445133	
**MM129**	0.088620246	5.421023598	2.526057909	3.000317387	2.918699952	1.221591183	4.86401901	-3.382980174	
**MM135**	-2.236636739	5.366462125	2.376968553	3.96038919	2.142207536	0.942709368	4.522005015	-1.635904977	
**MM137**	-2.489872718	6.370458967	2.101543401	2.890056915	2.117459651	-0.568767332	4.399711664	-3.586786253	
**MM006**	-1.555996896	5.664342919	2.933922601	3.666691046	2.530439807	-1.700725682	3.890583616	-4.482019291	
**MM008**	-1.262568925	5.114198985	2.968101716	3.301937342	2.777570055	1.457887373	3.93732594	-0.354962963	

Supplementary Table S5. log2(RPKM) expression values for 39 aminopeptidases genes and 17 housekeeping genes in MM patient samples in the FIMM dataset (*n* = 122) (10/21)

	Aminopeptidese genes								
**Gene symbol**	***NAALADL1***	***NPEPL1***	***NPEPPS***	***PEPD***	***PGPEP1***	***RNPEP***	***RNPEPL1***	***TPP1***	**Gene symbol**
**Sample_ID**	ENSG00000168060	ENSG00000215440	ENSG00000141279	ENSG00000124299	ENSG00000130517	ENSG00000176393	ENSG00000142327	ENSG00000166340	ENSEMBL ID
**MM032_1**	-2.447161258	0.850950493	0.932287414	0.861644522	1.815242644	1.4692232	-0.080886529	1.961788755	
**MM051_1**	-2.458848151	3.001369701	1.810807881	1.425664772	2.881682408	1.480547528	2.496297577	3.237727995	
**MM053**	-0.040128233	1.922031172	0.258387805	2.956369086	2.469934308	1.350328998	2.067943745	1.179435764	
**MM056_1**	-4.90717374	-0.193309708	2.155460778	1.648619656	2.114265851	2.737223869	-0.915129514	0.658266711	
**MM063**	-4.032224457	0.538480288	2.240005782	1.996760903	1.254821651	0.890112911	-0.683381803	0.200037492	
**MM064**	-3.769429867	0.336152286	1.407837635	0.876256253	1.508343686	1.881860261	-0.861069625	0.345594407	
**MM065**	-1.280797919	1.579710434	2.476692762	1.04476302	1.418059286	2.732876338	0.479478555	2.612445633	
**MM066**	-2.96913531	1.395998453	2.261963212	1.286767379	2.489653017	1.970553704	0.553446459	1.629898604	
**MM067**	-3.55874361	0.710476698	1.968843898	1.113186452	1.550489897	0.563554004	-0.953082289	0.695275688	
**MM070**	-0.425668349	0.951187621	2.09734047	1.680471858	2.700470028	0.816746715	-0.480230169	2.070232722	
**MM074**	-3.023634706	-0.172530771	2.064858309	0.361199369	1.794479401	1.234106264	0.60031081	2.98606365	
**MM075**	-2.159019708	0.223462423	1.395604891	0.482454698	1.872696359	0.852412062	-1.675548498	1.190253556	
**MM078_1**	-1.226491657	-1.3271663	0.92910348	1.090050116	1.047793312	2.926413321	0.230081569	1.683292876	
**MM085**	-5.058100552	-0.974861037	1.010202294	0.397454571	1.724145631	0.636932084	-1.556283414	0.551209172	
**MM086**	-1.888209038	1.03545971	2.166754042	1.470164895	1.48531264	1.362352593	1.005641829	2.895429862	
**MM087**	-6.551886405	0.405287183	2.20289165	1.99706848	2.314899828	3.351918966	1.267720958	0.019095623	
**MM088**	-2.697604904	-0.558868976	0.508091191	1.046072232	1.136700622	0.415832106	-0.541187028	1.46835829	
**MM089**	-5.554898197	0.044213728	2.331542674	0.341633565	2.306806567	0.213185195	-0.292764807	1.803580114	
**MM090**	-5.340117012	-0.163014983	2.736888359	1.384065898	1.819130618	1.264493713	-0.401005956	0.770090245	
**MM093**	-4.696642706	0.774595804	2.141317385	1.172457689	2.248024472	1.957418031	-0.113113603	1.169802808	
**MM094**	-2.175506268	0.773375748	2.26579871	1.210559136	2.213330002	1.855693208	0.604525944	2.385936611	
**MM096**	-4.392580794	-0.264363458	2.145261921	0.6441445	2.507622177	2.018423173	1.066107994	1.60384957	
**MM101**	-3.687358811	0.737234203	1.662988849	1.264903381	1.738133727	1.937743999	0.012204689	2.143872496	
**MM114**	-5.649221229	-0.282537507	3.056583403	1.173470411	1.336759467	0.974561805	0.293313753	1.813603664	
**MM115**	-4.838144862	0.651881404	2.743945206	1.691331015	2.811199701	2.118780502	0.749272902	1.385220982	
**MM117_1**	-6.080114506	-0.734307355	0.880400005	1.018039066	1.604628893	1.45060447	-1.242429254	-0.367360436	
**MM118**	-4.515367818	0.585956857	2.29406924	1.345947375	2.056163378	2.234467878	-0.296723103	1.450677192	
**MM119**	-3.320108456	-0.451490557	2.020056413	1.563471165	0.607468099	0.761732785	-0.084721932	2.13920327	
**MM121**	-3.644633171	0.077688929	1.89039487	1.018605524	1.570227733	1.490422932	0.007054185	1.99123928	
**MM122**	-2.12958096	-0.0080976	1.399897421	1.425843563	1.67268604	1.89874786	-0.231801137	1.241112216	
**MM123**	-3.439335071	1.264625614	3.000396261	1.168308253	1.5217816	2.055187837	0.739467924	1.810307335	
**MM126**	-3.882843137	0.132997322	1.794436663	1.005733102	1.670428267	1.270819776	0.160475501	2.051293567	
**MM128_1**	-4.72161761	0.475767434	1.087965007	1.425853005	2.077148174	0.873663175	-1.000538294	-0.448857896	
**MM131**	-10.4426507	0.530015123	1.737485533	1.469238538	1.823058945	2.302180965	0.376078959	1.445277221	
**MM132**	-3.89187127	-2.544743978	0.618511109	1.705643428	0.698827965	1.04052653	-1.667949582	0.332604145	
**MM133**	-3.267029215	-0.208229299	1.317851428	1.026154124	0.939968045	1.682168046	0.022599975	1.491108899	
**MM134**	-2.90518936	-0.664670169	1.331124753	0.956239125	1.17537829	0.9173676	-1.413172215	1.625177261	
**MM136**	-2.398810185	-0.028672045	1.817113329	1.531056415	2.360329991	1.613376725	0.873687451	0.72125103	
**MM138**	-6.606925288	0.236647482	1.711303492	1.376009139	1.648021569	0.856436615	-0.382706018	0.925029537	
**MM139**	-1.459092636	-0.16588482	1.550493836	0.719421576	1.553506158	0.267564756	-0.421795249	0.778639701	
**MM140**	-5.455328363	-0.315856554	1.804512472	0.365874684	1.385514901	0.762683358	-1.073532762	1.836930339	
**MM026**	-3.58864668	-0.831260831	2.121607173	0.467180477	0.76645568	2.10588193	-0.15115475	0.938526813	
**MM027**	-2.771494219	0.326307253	2.704825716	0.794710321	1.493990236	1.181807508	-0.831580508	1.384229369	
**MM028**	-2.904827811	0.491357688	1.898264591	1.467772968	1.40437916	1.588249146	-0.379579789	1.974383525	

Supplementary Table S5. log2(RPKM) expression values for 39 aminopeptidases genes and 17 housekeeping genes in MM patient samples in the FIMM dataset (*n* = 122) (11/21)

**Gene symbol**	*NAALADL1*	*NPEPL1*	*NPEPPS*	*PEPD*	*PGPEP1*	*RNPEP*	*RNPEPL1*	*TPP1*	**Gene symbol**
**Sample_ID**	ENSG00000168060	ENSG00000215440	ENSG00000141279	ENSG00000124299	ENSG00000130517	ENSG00000176393	ENSG00000142327	ENSG00000166340	ENSEMBL ID
**MM030_1**	-3.618947972	0.385905704	3.266619708	0.416794883	2.07132849	2.103451582	1.010132223	2.316969709	
**MM030_2**	-2.359553874	0.709716281	2.514085921	1.913471058	1.861324194	2.953419947	1.175014671	1.679079076	
**MM031_2**	-1.114018823	-0.834483658	1.695134072	1.264471941	1.774351922	1.224431263	-1.240432967	0.42714702	
**MM033_4**	-0.694571222	1.375708461	2.814393847	0.766140464	2.492137501	2.291400859	0.153515914	2.253865387	
**MM033_1**	1.035754092	2.838701803	2.228733572	2.59883303	3.075257609	3.336494397	3.260573366	3.158241182	
**MM033_2**	-1.186658763	0.568306121	2.091590016	1.247829716	2.392877537	2.297992841	-0.68766064	1.919530853	
**MM034_2**	-1.183687968	0.285506304	2.273652359	2.087655262	0.850731383	2.507943519	-0.876371378	2.180023493	
**MM034_1**	-3.672418022	0.150825333	0.358803983	1.688235953	2.077275213	1.560183378	-1.022487144	1.894181337	
**MM037**	-2.770037723	-1.041713861	2.17158921	1.42053598	1.09829541	1.688543023	-1.26255111	-0.753657194	
**MM041**	-3.175361253	1.287666721	2.120912881	1.882826832	2.678686933	1.685605255	0.955170079	0.415242062	
**MM042**	-2.799364572	1.762623732	1.620382386	2.73143202	2.966531905	2.873641965	2.320504854	2.557846191	
**MM043_1**	-4.434070075	0.288598095	3.634202157	1.051821479	0.720707824	2.540596932	0.477353044	2.594647816	
**MM043_2**	-3.653103188	0.870406488	3.216132778	2.256016559	1.896635136	2.682766103	1.487191169	2.877184553	
**MM044**	-1.751696939	1.031953922	1.099807102	2.162614847	2.347175859	2.199959561	2.0446125	2.075661359	
**MM045**	-1.69446781	1.179109939	2.378398966	1.280339856	2.338929393	1.60623505	0.303053929	2.897602304	
**MM046_1**	-1.570408723	0.785107288	2.963928143	2.011708136	3.289912555	2.306211049	1.727929328	2.076570801	
**MM046_2**	-1.311573704	1.389518787	3.003981528	1.399041963	3.411826873	2.013235201	1.654768522	2.25829581	
**MM048_2**	-10.5494121	-1.015149324	1.815746924	1.199138651	1.16412409	0.679624283	-1.072079839	1.614423371	
**MM050**	-5.009534895	1.078335718	2.407048947	1.131012375	2.007446653	1.790733427	-1.175005375	2.158435164	
**MM051_2**	-3.84816258	1.335539902	3.271436757	0.69826643	1.711291973	1.652839282	-0.072410344	2.099431909	
**MM054**	-4.193209364	1.71257047	0.556132345	2.334219773	1.216262347	3.92328438	2.285730481	2.052233866	
**MM056_2**	-5.133944623	0.825641624	2.039344506	1.290916083	2.327047626	1.830539995	-0.272569349	0.498582154	
**MM057_4**	-3.277568718	1.171613218	3.136570516	1.868429485	1.534367875	1.681193276	0.474439241	2.008948193	
**MM057_5**	-2.544333202	0.242817842	3.071757884	2.097136161	1.113843547	0.726164329	-0.090322534	1.363065361	
**MM057_6**	-3.407634355	0.318243371	2.172316354	2.523783935	0.779718874	0.838882887	-0.87920243	1.031777987	
**MM057_1**	-3.172481913	1.624465177	2.712764899	1.585387763	2.268887166	1.815300474	0.80185257	2.815526651	
**MM057_2**	-2.828725962	0.93188231	2.705079442	1.477048995	2.342194941	2.433665879	1.077543899	3.253228764	
**MM057_3**	-3.072714394	0.592430979	1.715618791	2.26945601	1.457721361	0.985112015	-1.430376741	2.095179028	
**MM058_1**	-4.221015092	0.370320511	2.4438292	2.451210282	2.516084296	2.653523805	0.302669325	0.7490991	
**MM059**	-5.249302279	-1.155037146	1.822592363	1.174833837	2.01363084	2.317072512	0.425170783	1.572712529	
**MM060**	-1.525234137	0.173805794	2.102619715	1.347112322	1.038719938	1.886107859	-0.67153	0.635369187	
**MM061_1**	-4.19964537	0.673048244	1.517397565	1.368837041	1.890038011	2.786374337	-0.516461932	0.408047383	
**MM068_1**	-4.665678688	0.037157732	1.28687767	1.404171771	1.275381003	1.435978809	0.417418821	2.622043668	
**MM068_2**	-3.901029646	-0.455452068	1.196421407	1.417491702	1.630198522	1.070414357	-0.739397855	0.057753378	
**MM091**	-5.11076725	0.523970238	2.913028267	0.558682927	1.457907137	2.352649186	-1.146624523	1.3593139	
**MM095**	-3.319991515	1.381604938	2.662382641	1.73209445	1.958985753	1.74269537	0.67649263	2.865873246	
**MM002_1**	-1.666137141	-0.087951434	0.947338292	1.022569433	1.708006374	0.197967637	-1.677584306	0.436829188	
**MM002_2**	-1.804787469	-0.318571656	1.352490456	0.813210173	1.657900901	0.172036077	-0.627091005	2.231534247	
**MM002_3**	-2.19460994	-0.327592172	1.29086625	1.158920488	1.569905745	-0.220645668	-1.369688347	1.527330403	
**MM003**	-2.912723595	0.660097041	2.945705462	1.45650911	2.732068076	2.899613556	0.889003487	1.891657343	
**MM005**	-0.017714456	-0.612236661	2.057076617	1.565569607	1.074446017	2.038079725	-0.467945576	1.067623146	
**MM007**	-5.776885125	0.681708723	2.410036865	2.026999116	1.158447383	2.821640906	-0.115051425	1.552281373	
**MM010**	0.271896147	0.573661135	1.752777813	2.084543581	2.319969227	1.123290192	-0.525803109	1.354468868	
**MM011**	-6.121799714	0.709203893	2.847554626	1.560343902	2.418982155	3.69270594	0.167140798	2.91445711	

Supplementary Table S5. log2(RPKM) expression values for 39 aminopeptidases genes and 17 housekeeping genes in MM patient samples in the FIMM dataset (*n* = 122) (12/21)

**Gene symbol**	***NAALADL1***	***NPEPL1***	***NPEPPS***	***PEPD***	***PGPEP1***	***RNPEP***	***RNPEPL1***	***TPP1***	**Gene symbol**
**Sample_ID**	ENSG00000168060	ENSG00000215440	ENSG00000141279	ENSG00000124299	ENSG00000130517	ENSG00000176393	ENSG00000142327	ENSG00000166340	ENSEMBL ID
**MM014_4**	-5.12378403	0.201517295	3.166754761	2.05734505	2.546474188	3.663266262	1.541642958	2.715440068	
**MM014_2**	-10.13140863	0.160675385	1.78859466	2.11105131	2.526910996	1.784840309	-0.019735977	1.226562872	
**MM017_1**	-6.397594851	-0.664099474	1.118661899	1.250381286	1.913607979	1.955149316	-1.488522835	0.435480156	
**MM017_2**	-2.749829144	0.191798228	1.26563279	1.544078037	2.43056925	1.953465521	-0.56833039	1.42328036	
**MM017_3**	-5.094324277	-1.116907783	1.915403327	1.664040568	1.867818421	3.061665496	-0.41559163	1.787292754	
**MM018**	-4.376035825	1.220490574	2.812871558	0.710987103	0.94495191	1.661640289	0.363361845	2.914454292	
**MM019**	-0.038817151	0.095177928	1.657153615	2.004110381	1.586017526	0.933074718	-0.548527424	1.793339754	
**MM020**	-1.511210353	0.415064321	2.315379265	1.838184207	2.476193815	2.16360711	0.380869957	1.768543745	
**MM022_1**	-1.377261168	1.698627321	2.40361968	3.518950741	2.616064085	2.644870747	2.727930362	3.8852361	
**MM022_3**	-5.825376856	-0.946647641	2.81789569	1.908875227	1.341004959	1.264996616	0.18244628	4.057868816	
**MM024**	-2.835777366	1.545518565	2.667859353	2.090748888	2.190380841	1.769662273	0.808481596	1.961420029	
**MM025_1**	-4.96245163	0.245726823	1.680375828	1.413338348	1.138836786	1.732355638	-0.576643574	0.583911955	
**MM036_1**	-1.959987958	-0.758329866	2.196061416	1.747527367	1.286121664	1.627353971	-2.583507341	-1.246983195	
**MM036_2**	-2.748730384	-0.362794806	2.43984435	1.222655486	1.245907181	1.071654314	-1.796815089	0.59447726	
**MM038**	-3.678964723	-0.191599083	2.087920919	1.640316188	1.953707416	1.22412726	-1.400602422	-0.370659403	
**MM039**	-1.240100874	2.450371516	2.436220018	1.744139685	1.730448426	0.887365559	0.136918003	1.8134582	
**MM049**	-2.8637663	0.997447475	2.117817075	0.943919261	1.778306469	0.341716016	-0.549770741	1.550700074	
**MM071**	-2.861277027	0.739279649	1.650372335	1.08162729	1.373422678	1.248116367	0.817213522	1.692631488	
**MM080**	-3.543657018	-0.539505007	2.136196026	1.24071123	1.062296873	1.623525265	-0.167778851	1.556394748	
**MM081**	-3.842783716	1.256114876	3.262619077	1.349833043	2.320224925	3.18882006	1.715979637	2.106365062	
**MM082**	-4.584482386	-0.649169642	-0.02438519	0.998757096	1.460043456	-0.244407078	-1.93968064	-1.23087675	
**MM098**	-2.421560669	1.276108075	2.865802399	2.164783851	1.261159028	2.731249616	0.554740499	1.97122729	
**MM102**	-3.578980112	0.74921145	2.194153674	1.559291387	1.122220179	2.715059891	0.313145861	2.757335989	
**MM109**	-4.996818283	0.02521592	2.517333696	1.240004055	0.162269175	2.833673931	-0.637308911	1.255863082	
**MM111_1**	-9.824865118	0.16779915	2.438018346	1.081550372	1.269224855	3.78416003	-0.128909035	-0.078403914	
**MM111_2**	-5.905720096	0.984151709	3.743501009	1.291454256	1.634920371	3.728106565	1.568934573	1.042101477	
**MM113_2**	-2.426868846	0.781078995	1.633670516	2.364443734	1.240241287	2.958823629	-0.04444585	1.509203892	
**MM117_2**	-7.156460745	-0.146889142	1.845225516	1.768897776	0.98307114	2.227681662	-0.196240031	-0.111132363	
**MM124**	-5.660622062	-0.297704385	1.688580826	1.274110293	1.628499438	1.071975009	-1.082879047	-0.576564139	
**MM129**	-4.222724142	0.541349908	2.57829901	1.640295621	2.064175367	3.434698309	1.011965774	2.354623075	
**MM135**	-0.676215004	0.376262258	2.400837024	1.916737322	3.05013017	1.575455929	-0.564871588	0.142966097	
**MM137**	-3.915374825	-0.842711342	1.108675789	1.208419678	2.420284022	1.209620708	-1.524117921	0.271729897	
**MM006**	-3.278435828	-0.178914008	1.197686669	2.023444957	1.564806724	2.60606951	-1.236105983	0.178459532	
**MM008**	-3.369365802	1.229254762	2.990429875	1.706258561	1.46506576	2.141563305	1.172280369	1.239098832	

Supplementary Table S5. log2(RPKM) expression values for 39 aminopeptidases genes and 17 housekeeping genes in MM patient samples in the FIMM dataset (*n* = 122) (13/21)

	Aminopeptidese genes					Housekeeping genes			
**Gene symbol**	***TPP2***	***TRHDE***	***XPNPEP1***	***XPNPEP2***	***XPNPEP3***	***ABCF1***	***ACTB***	***ALAS1***	**Gene symbol**
**Sample_ID**	ENSG00000134900	ENSG00000072657	ENSG00000108039	ENSG00000122121	ENSG00000196236	ENSG00000204574	ENSG00000075624	ENSG00000023330	ENSEMBL ID
**MM032_1**	3.011407013	-2.840812767	1.953734202	-4.273221559	-0.226257169	3.24559661588632	6.96037800736663	1.2472109244613	
**MM051_1**	3.445118395	-5.896472984	2.747793963	-4.525427123	-0.918877511	3.56096114440767	7.2974798383447	2.5225310574189	
**MM053**	1.764428525	-7.69888384	2.070615615	-4.983883577	-2.281845125	3.40814142840279	8.05798722424296	1.33790228951546	
**MM056_1**	4.133894133	-3.673063351	2.480129579	-5.412441479	-0.066676524	3.74346742570021	6.03666342709802	2.87738106132657	
**MM063**	3.266756233	-5.220984658	1.970432873	-10.16364638	1.568377167	4.06104473469052	6.0935462139343	2.05478841108064	
**MM064**	3.452285633	-11.87174439	1.653441358	-10.1001606	-2.07416922	4.19885469278407	6.5942274174496	3.21814407507454	
**MM065**	4.615464434	-1.171168438	2.94055072	-4.19890225	0.042972694	4.49660377087986	6.63992988515032	2.34437493118945	
**MM066**	5.051752665	-5.404283414	2.687684763	-5.273157236	0.1454406	4.29329581674373	7.02731939697441	1.53567770365702	
**MM067**	4.002957622	-8.772153286	2.199132511	-6.083031656	-0.286312816	3.66208692066372	8.24558341549583	2.62101603809064	
**MM070**	2.377952795	-4.53777134	2.319210479	-3.691333554	0.104400432	4.03884259817758	7.48243077914581	2.31210243381855	
**MM074**	3.863252702	-6.352325168	2.939086438	-7.433184188	-0.271468532	3.70935687692464	6.90376576265991	1.44524391719938	
**MM075**	3.68105197	-8.324710554	1.971558474	-5.782608609	-0.248043027	4.6042239183602	5.97968777019744	1.91729796277638	
**MM078_1**	3.857244256	-5.551150076	2.944976808	-10.87759837	0.055688236	3.19444808363451	6.49180386827821	3.2125681989203	
**MM085**	4.316285652	-3.578044201	2.544317438	-4.03675803	-0.609271568	3.29130023555254	6.58321119158007	1.05403191575401	
**MM086**	2.900891435	-7.411134909	1.989502735	-3.228124872	0.807132231	4.81646839836315	8.4776662811831	2.04751266251996	
**MM087**	4.584459001	-11.28496712	3.043227092	-6.34345833	1.053397727	4.1757825141209	6.65234756509436	2.94894919310529	
**MM088**	2.911557944	-7.26621505	0.771957471	-10.53902538	-0.326789499	3.70765582290545	6.53673871961377	0.997907402801395	
**MM089**	4.467756495	-5.183148941	2.60937429	-4.389538843	0.218243583	3.76041056145067	6.74966020013408	1.34941002370988	
**MM090**	4.234005082	-6.689147923	3.109866654	-7.453617032	0.150440784	4.37668218513828	6.07608915194486	1.63835220555715	
**MM093**	3.409778096	-4.03571765	2.52968356	-10.41895197	0.03804903	4.12816864088083	8.17786353107524	2.02062127610502	
**MM094**	4.405195807	-7.490402931	2.396642772	-3.092036463	0.73665813	4.50903450449253	6.56784769440085	1.60469343644725	
**MM096**	4.776713203	-9.385724351	2.732600622	-10.78406556	0.856429179	4.71851592068368	6.47272108873626	1.31599927168514	
**MM101**	4.266391238	-4.741042226	2.271054074	-9.888321672	-0.182110106	4.83141062332908	6.63162481076533	2.18056836575089	
**MM114**	3.052282088	-11.85623313	1.803253708	-5.040255224	0.89115852	3.22281489876044	6.62370922258413	1.92945420126461	
**MM115**	4.623321893	-9.694082327	2.957777635	-10.24442663	-0.064561709	4.72274748796174	6.40998816306183	2.40307825626753	
**MM117_1**	2.610053232	-12.28712641	1.62122041	-7.345617619	-0.995047656	2.67641687435691	5.85199914423668	1.97197212932144	
**MM118**	4.1377878	-4.527113593	2.755275458	-9.164920738	-0.231903872	3.43111879858484	6.22462732394046	2.56725137007004	
**MM119**	2.482989149	-11.84904846	1.973688186	-7.75553657	-1.219755195	2.77193006282202	9.31623438452382	1.11270176935053	
**MM121**	4.545889866	-6.372175704	2.490921509	-3.843863064	-0.011738405	3.69273437606265	6.88544764086564	1.81438704109966	
**MM122**	4.131233301	-5.287580688	2.832343896	-3.706518803	-1.231170064	3.37496119725216	7.36454619782611	3.15160873299272	
**MM123**	5.311166188	-5.667306596	3.011070497	-3.638564965	-0.172685768	4.49452437562744	6.56600296052359	2.09493235368946	
**MM126**	3.45343349	-8.384598309	2.273892542	-5.695476678	-0.297332583	3.27719586875901	6.94943268362347	2.15404377374643	
**MM128_1**	4.153952442	-9.045585662	2.000719954	-10.44392687	-2.683805462	3.05236446142949	5.6894635693427	1.80918380788496	
**MM131**	4.423788763	-12.00580641	2.461291353	-10.23422262	0.951767439	4.44693376234464	7.37950508371029	2.43055656175823	
**MM132**	4.129483066	-4.795732288	1.175657498	-4.523502282	-2.042489384	2.22781834158281	4.99435375294729	1.48752547618373	
**MM133**	3.815061584	-8.758292012	1.941406533	-3.966808663	-1.068272068	1.97850889696971	6.65515538914022	1.96682474635592	
**MM134**	2.941752142	-11.86922451	1.44043699	-1.807621874	-0.66294891	3.13199841559433	7.27797621689806	2.06108194343084	
**MM136**	4.677611015	-11.42957145	2.289215249	-9.65798766	-0.394650535	3.78877096295582	6.48237576736036	1.63097572614416	
**MM138**	2.896506713	-12.25754384	1.541526833	-6.785520336	0.429960818	3.36426643838489	6.88476047337684	2.12636306712472	
**MM139**	3.187215727	-3.567460204	1.749809989	-7.857172592	-1.466938411	3.81216412836965	6.24262307266849	1.90861560935316	
**MM140**	4.047980379	-8.492415267	2.012989645	-9.890756477	-0.428885933	3.34027668003763	6.41762944228324	1.59987536752465	
**MM026**	2.662140985	-5.927762122	2.924083351	-5.7021467	-0.321529868	3.54917425466503	5.69270241040316	1.55602918771219	
**MM027**	3.365464472	-12.19883608	2.513726113	-7.257327287	-0.466978658	5.11376980086037	7.88625061647482	2.39976570405575	
**MM028**	4.202936557	-2.457830811	2.324375041	-3.449759768	-0.212227492	3.37792971619463	7.58897866099255	2.10694169382992	

Supplementary Table S5. log2(RPKM) expression values for 39 aminopeptidases genes and 17 housekeeping genes in MM patient samples in the FIMM dataset (*n* = 122) (14/21)

**Gene symbol**	***TPP2***	***TRHDE***	***XPNPEP1***	***XPNPEP2***	***XPNPEP3***	***ABCF1***	***ACTB***	***ALAS1***	**Gene symbol**
**Sample_ID**	ENSG00000134900	ENSG00000072657	ENSG00000108039	ENSG00000122121	ENSG00000196236	ENSG00000204574	ENSG00000075624	ENSG00000023330	ENSEMBL ID
**MM030_1**	4.93698699	-6.927530861	3.327658743	-3.515489457	0.50510881	5.48111388581112	6.13132215310727	2.53936808750813	
**MM030_2**	4.070586018	-12.03645176	2.879166434	-4.075043406	-0.218284241	3.34364930297205	8.04027049846687	2.91782279574533	
**MM031_2**	3.941687513	-12.44038691	2.372983581	-3.657575859	-1.405465989	1.88552761267273	5.46355142015444	2.67040213867297	
**MM033_4**	4.355291417	-4.619593778	3.315893975	-5.510975	0.836719636	5.33967978993212	6.44420397844458	2.72421377249021	
**MM033_1**	4.57669183	-6.268075267	3.308719951	-8.583954317	0.868913421	4.66645500230302	7.26946226408015	2.72711977083584	
**MM033_2**	4.206033623	-4.911834282	2.870854031	-9.070987828	0.332892454	4.332741460803	6.73470823786078	2.89016683689775	
**MM034_2**	4.091111494	-1.776654617	3.693075763	-2.31047539	-1.047263493	2.65341540852458	8.81329289311382	2.89583975515396	
**MM034_1**	3.377850446	-4.509138811	2.17889237	-5.215602317	-0.218346925	4.51510813317046	8.15266656499034	2.26680167827351	
**MM037**	3.627995993	-5.866625638	2.07549436	-6.75800686	-0.894875851	2.10861383302995	6.02369785583813	2.11853967357081	
**MM041**	4.915590357	-3.890520062	2.963029851	-5.845254621	0.593153344	4.62814099370706	6.972701985871	2.09098169567398	
**MM042**	3.424755481	-4.454150763	2.901235383	-3.96944812	0.202885891	4.09908601067303	7.47271785239059	2.8151144844108	
**MM043_1**	4.41740083	-6.845222698	3.017962284	-4.673100977	1.32584353	3.91372409227887	6.64372332111756	2.1465830046573	
**MM043_2**	4.546222257	-5.216258903	3.156756312	-1.911242912	1.312669296	4.51403537751017	7.60980244160383	2.61821022268171	
**MM044**	4.018180022	-2.061844866	3.12611683	-6.916375683	-0.287177032	3.61565554192463	8.07438486716013	2.34175684899821	
**MM045**	4.528306483	-4.737917217	2.887891914	-3.595814593	0.065403731	4.98688959125055	7.98237515252393	2.85757317447125	
**MM046_1**	4.818799918	-0.255022121	2.664899809	-2.530180969	0.871747376	4.62354021969656	7.65549226207632	2.81384011588497	
**MM046_2**	4.774187512	-2.383595027	2.751025174	-6.117069871	0.735101415	5.19152930995341	7.90408369460119	2.99865823950312	
**MM048_2**	4.046481241	-12.11256782	1.316839667	-10.34098403	-0.097087614	5.16496021484653	7.53932404975748	1.81773863833366	
**MM050**	3.504638888	-6.358565805	2.919521204	-4.40056889	-0.725553096	4.70701901925251	5.53930513939318	1.57921425776793	
**MM051_2**	4.919655208	-7.325588422	3.537013341	-3.232076536	-0.655002292	3.8691107968386	6.95908961992365	2.00558379366001	
**MM054**	1.964201853	-6.731274099	2.315064193	-6.117231585	-1.113355111	3.00792962735413	6.61408293470023	1.93079604069144	
**MM056_2**	4.352671291	-12.05465234	3.083632168	-10.28306855	0.080948108	4.46793270475121	7.00897266704445	2.86845099029819	
**MM057_4**	5.312987445	-11.03054899	3.685463937	-9.258965201	1.190511947	5.50075498022874	6.86046447572921	2.30189121452583	
**MM057_5**	4.446968901	-6.811750347	3.581472577	-6.72822255	0.457842969	5.02486006801969	6.30127701899673	2.85349912828679	
**MM057_6**	4.588776105	-12.4054183	3.259286254	-10.63383451	0.634856647	3.55615519245057	6.12506972695994	2.8041248832771	
**MM057_1**	4.795311139	-6.349454992	3.338537701	-3.599897508	1.588858654	6.02228782156641	7.24064155004523	2.22028765090947	
**MM057_2**	4.149992478	-2.977759885	3.470747642	-3.766616275	0.860712051	4.84359822858367	8.37928556423049	3.1884150807422	
**MM057_3**	4.435784827	-7.265620532	3.412894403	-10.70349011	1.861638329	4.92915813508374	6.84792000440369	2.17837141651631	
**MM058_1**	4.803211077	-4.212444135	3.088907879	-4.093887119	1.142875061	4.23627565424522	6.45248423143632	3.17396784193094	
**MM059**	4.329571678	-8.686927113	3.115654188	-10.08526832	0.310413697	3.95691329481672	6.21752686161239	2.37847480504637	
**MM060**	3.338763289	-12.04549189	2.967164306	-7.103983101	-1.69638776	3.41694717978468	6.04019924762178	2.54761913114571	
**MM061_1**	3.715556828	-2.770231291	2.159779265	-3.991217295	-0.660546889	2.62536207916491	5.85618832190499	1.81172396417327	
**MM068_1**	2.655064235	-2.159114385	2.466912957	-5.10932731	0.224274865	3.65134444369025	7.71007033026749	1.32994598981575	
**MM068_2**	3.274807014	-3.27364419	2.396680745	-3.692601571	-0.381199098	3.28041160593831	6.43218646169226	1.64862012253239	
**MM091**	5.231216757	-6.009107157	3.717990924	-10.25989118	-0.344789477	5.83225460326554	5.99650921894902	2.05079103154485	
**MM095**	3.706605619	-12.06305632	2.04293336	-7.121547528	1.292418859	4.72085489484467	7.80410977014066	2.09107266050421	
**MM002_1**	2.900986793	-2.352195536	1.689039069	-3.882424479	-0.997907975	2.67383238131506	5.99471526721365	1.9656812168432	
**MM002_2**	2.792435415	-6.429190374	1.631429695	-6.571876709	-0.87467821	2.99721226697655	7.09990402078269	2.63361237117651	
**MM002_3**	2.823996417	-12.22129003	1.571258344	-7.279781237	-0.85639607	2.40873646561422	6.85633689055663	2.29267194068513	
**MM003**	4.45206639	-4.879672053	3.642457452	-5.795148951	1.460305234	4.83303683730214	6.89152014442591	2.99103820861685	
**MM005**	4.902402546	-2.232322933	2.815191218	-7.006776131	-0.856153443	4.28215490258318	7.35644481827347	3.29950103397489	
**MM007**	3.640558296	-7.34004084	2.328262711	-9.655919891	0.004100961	3.77475164640274	7.17674648209718	2.50280277401791	
**MM010**	4.62034916	-12.15931042	2.521817993	-2.599824072	0.897965103	4.29359972696081	6.15293008105401	3.11684939420142	
**MM011**	4.264347739	-11.77241827	3.327054135	-10.00083448	0.667960532	6.18958820850488	7.92534939331229	2.92467754845321	

Supplementary Table S5. log2(RPKM) expression values for 39 aminopeptidases genes and 17 housekeeping genes in MM patient samples in the FIMM dataset (*n* = 122) (15/21)

**Gene symbol**	***TPP2***	***TRHDE***	***XPNPEP1***	***XPNPEP2***	***XPNPEP3***	***ABCF1***	***ACTB***	***ALAS1***	**Gene symbol**
**Sample_ID**	ENSG00000134900	ENSG00000072657	ENSG00000108039	ENSG00000122121	ENSG00000196236	ENSG00000204574	ENSG00000075624	ENSG00000023330	ENSEMBL ID
**MM014_4**	3.868870336	-6.821240838	3.597916377	-6.31974621	1.393245978	4.00202092569255	8.04652078988157	3.29435278884074	
**MM014_2**	2.648327128	-3.280936422	2.115311733	-5.064999564	0.054136109	3.39894691240249	7.94897319922125	2.51084631923189	
**MM017_1**	4.052443476	-8.34777369	3.051258998	-6.189166776	-0.210235145	3.94479233346429	5.98024615326614	2.94493534873198	
**MM017_2**	3.917087139	-5.230522699	2.877484264	-5.154932722	0.719909645	4.87007643120177	6.69632279642015	3.03369902156793	
**MM017_3**	4.220715835	-11.04979742	3.052094944	-5.190750783	1.337789949	4.67779913243436	7.34064679105686	3.28231322070191	
**MM018**	4.260064142	-0.965385481	2.313843061	-2.553790386	0.860283166	4.36141203827985	6.9533070570274	2.23650450038181	
**MM019**	4.276868852	-3.990243456	2.751115847	-6.306122506	-0.040817829	3.3073652241464	5.9038284331888	2.43014069818205	
**MM020**	4.302131296	-4.618212716	1.7989685	-7.174095203	0.95751003	4.27778436118815	6.51819790904272	3.21836899216053	
**MM022_1**	3.098295138	-10.72831944	3.157735497	-4.312879462	0.811271015	4.94837251946737	8.613889825974	2.22316853371684	
**MM022_3**	2.953686543	-6.431601294	2.486296365	-9.704411622	0.504263144	3.67479946814804	7.15635983180738	0.569352729223172	
**MM024**	4.813581541	0.2549245	2.866720425	-1.516705227	0.492718892	4.82945832797556	6.59181660090292	2.43023289116777	
**MM025_1**	5.076317643	-11.57000147	1.8311031	-9.798417674	0.397723516	4.33430261697006	6.34991043153092	1.47132017431641	
**MM036_1**	3.535576793	-7.978712653	1.777042783	-10.2945917	-1.319045743	3.96808089765936	5.53196309767626	2.85429958023427	
**MM036_2**	3.366173886	-5.479180845	1.807530299	-9.638334391	-1.044341614	4.47989449132922	6.00134710069923	2.35051770390846	
**MM038**	3.200452034	-5.272981403	2.108250928	-6.945316231	-0.927560999	3.38259505290136	7.02842410578549	1.73722786318937	
**MM039**	4.414591708	-12.23996813	1.767456238	-4.446016528	-0.277648196	4.66815313954843	7.17179069280536	1.84855516360173	
**MM049**	4.579118927	-11.19510634	2.36292337	-9.42352255	-0.640472003	3.32240885807162	6.90452600472229	3.21900859368182	
**MM071**	3.740425708	-6.880661929	2.881663652	-10.46663014	-0.491608132	4.77806836656544	7.06127348060372	1.94397961849586	
**MM080**	3.51576309	-8.757066695	3.140120948	-10.15540791	0.887930721	3.54755327484694	6.8526458042736	2.81247262311976	
**MM081**	4.854190294	-8.575864433	2.829547516	-9.974205643	-0.033118488	4.07035633242298	6.51653420678398	2.8150563890928	
**MM082**	3.224648982	-12.55702904	1.69608954	-10.78544525	-1.066661095	2.82092528639252	7.21666033921704	2.05031472338632	
**MM098**	5.494956475	-5.921785129	3.249668305	-4.863897153	-0.992293968	3.659175866456	5.82580790021557	2.42922995030775	
**MM102**	4.584876693	0.132961383	3.920264498	-2.643891451	-0.027301883	3.67587341038938	10.0117387243157	1.83114955579854	
**MM109**	3.828174164	-6.300886778	2.662074888	-7.549202544	-0.873404928	3.91889444042804	5.71381913312474	2.79637157560321	
**MM111_1**	4.399627903	-7.300557992	3.136181076	-9.616437043	1.086930528	3.77529299295699	5.81343885755134	3.41465506406085	
**MM111_2**	5.163990515	-8.847387435	3.655109462	-9.397731739	1.651592148	4.54954379092509	6.419183817207	3.09116921824731	
**MM113_2**	5.078185004	-5.357533691	2.744465405	-2.086596115	0.667168839	4.64976458346448	9.3067835103956	2.76088943541333	
**MM117_2**	4.069486415	-6.587166165	2.645554519	-7.478547387	-0.121171401	3.62483868651442	6.37777381889518	2.98703528982669	
**MM124**	3.99091541	-8.345768302	2.515028971	-7.491722351	-0.197161538	2.98044096745263	7.06360700853014	1.1391013892749	
**MM129**	5.159498559	-7.973506861	2.724813916	-5.284385231	0.06284374	4.86769419496972	7.68502228487962	2.45544391086412	
**MM135**	6.502120812	-8.366361925	2.713132092	-5.677240294	0.106021518	4.42433054409627	6.77978763944024	2.6604610499812	
**MM137**	3.764196217	-6.549915174	2.38932899	-10.88685584	-1.52724803	2.92258395412685	5.58237085493238	2.02218277521354	
**MM006**	2.875558191	-12.24247098	1.019724076	-10.47088719	-0.629757318	3.1718995610789	6.17308625842594	1.37166578243047	
**MM008**	4.82090679	-4.620308578	3.287319736	-4.547216233	0.063323441	4.43147038343367	7.68901337629311	1.97229535381589	

Supplementary Table S5. log2(RPKM) expression values for 39 aminopeptidases genes and 17 housekeeping genes in MM patient samples in the FIMM dataset (*n* = 122) (16/21)

	Housekeeping genes								
**Gene symbol**	***CLTC***	***G6PD***	***GAPDH***	***GUSB***	***HPRT1***	***LDHA***	***PGK1***	***POLR1B***	**Gene symbol**
**Sample_ID**	ENSG00000141367	ENSG00000160211	ENSG00000111640	ENSG00000169919	ENSG00000165704	ENSG00000134333	ENSG00000102144	ENSG00000125630	ENSEMBL ID
**MM032_1**	3.56951612495665	0.930889934423915	6.30059091471253	2.69537343855205	1.24452007505547	4.52977785737859	4.42290060580197	0.762665249241917	
**MM051_1**	3.24077204198667	0.981858772523486	6.53067654295999	3.29850164827529	0.527234612306259	4.99699172903846	3.99725130349479	0.258024068461999	
**MM053**	2.45183772755119	1.25509033950066	7.35066109233487	1.54060514220152	0.211382553937821	4.43988700689476	4.56905334052872	0.717923192919615	
**MM056_1**	4.86329557699317	-0.324589719042922	6.85299428120268	2.84356152653739	0.638345408423199	4.86667372885553	5.00684761651898	2.62301771331325	
**MM063**	4.25650495750469	0.048447204284162	5.75891953612445	2.87014607026288	0.816062142840675	5.04505270704376	4.27925667815306	2.20769149720021	
**MM064**	3.40318786224311	0.93575367243078	5.55939818346135	4.17673265844657	3.58247270234512	5.40731079457087	4.65356602399763	2.63264269140374	
**MM065**	4.90092662195435	0.640254543602264	6.00716648930756	3.38172954699413	3.17068256196627	5.72214544745329	4.35961307927262	2.43779377008345	
**MM066**	4.44794681207397	-0.049832728354314	5.41015787645259	3.48528139649519	1.44241150384895	4.63913686454978	4.58985537834014	2.19335238993386	
**MM067**	2.71182616540584	-0.275459383513566	6.11755359329303	3.09469053816694	0.22502117224736	4.63812695531604	4.05598413404758	1.15510283965066	
**MM070**	4.13065133917334	0.25009954877674	5.68324984970369	2.95449881111765	0.504885794523375	5.09068985739168	4.21267412143216	1.50113706924601	
**MM074**	4.04847724571645	1.09825917914901	5.83792176132872	2.72394205243987	1.10924732876616	4.69055855078382	4.25301938154333	1.26784744488929	
**MM075**	3.7132192427809	0.874252860581185	5.369585133378	1.65874891254782	2.4619206805445	4.4631172397707	4.43081255747748	1.78396301956444	
**MM078_1**	4.69648227888646	1.54379347916254	6.78566180619908	3.87190263733399	1.4231141403605	6.75185859954119	5.07489308320408	1.82296723737699	
**MM085**	3.22053764141627	-0.656971879917354	5.562769624309	1.59543081974979	1.08896716772429	3.97941740259767	3.8548433706405	1.14728511316225	
**MM086**	3.90978104557219	1.71140176426473	5.87789508869148	3.00421472021853	2.63536920217328	5.98113061330102	4.81014056804645	2.03642765124524	
**MM087**	5.26232851574068	-0.202595982174363	6.98072890729986	3.73422183182549	2.70098289897804	5.79783012840271	5.38561697683609	2.7799938653759	
**MM088**	3.10998954110092	-0.69640472894833	5.03547591570401	1.94032433477261	1.2063084631137	4.44182061284187	3.53034411241071	0.573938503667025	
**MM089**	4.10526430739956	-0.106097320344371	4.85604017382879	2.38788694198549	0.785005997196098	3.98594030536029	3.59924024326794	1.2057999952768	
**MM090**	4.87723426241207	-0.67682611913113	5.67990200223337	2.43264004660273	1.4628287255876	5.2265676408451	4.79930497626898	2.53615225283171	
**MM093**	4.39902452753975	0.021914400887926	7.18187163305547	3.76231911490294	1.34795528464357	5.89185144554959	5.18335652165933	1.03504883108361	
**MM094**	3.91223479979999	0.44207922172174	6.46447409047065	3.47576342713141	1.03114205223096	5.25581726413854	4.36465765996553	1.95035076215982	
**MM096**	3.90120412789047	0.895886444509647	6.91351366803316	3.78341136972363	1.14471442072109	6.57939370060566	4.32021721563664	2.68865391162716	
**MM101**	4.23238165170858	0.476119287327575	6.28586591846428	3.34306591561405	1.78267788945268	5.53734559658098	4.78374671795904	1.78477702630303	
**MM114**	5.2544448415918	0.168181870197684	6.24397428377823	2.40793012726456	1.56328198428736	4.59618560300048	4.14629779359878	1.78289515026968	
**MM115**	4.8546453025031	0.192516703941239	5.74350580518722	3.03558555911041	1.4379701860404	4.8053554818	5.19895784981784	2.29286555241725	
**MM117_1**	3.70552476520743	-1.27503741946818	6.19902865368525	2.8484498546236	0.201439407372894	4.52471497186021	4.68142427711006	1.45499228871456	
**MM118**	4.81015437475786	-0.375151971043462	6.4444244887483	2.40396210063149	2.18724638687951	5.35480472845106	4.51103588803647	2.56000826934812	
**MM119**	4.2481144474336	-0.26480760699877	6.31650488047901	3.33559336888416	0.962553376377507	5.41300989691334	5.49560757781335	1.5529872528903	
**MM121**	4.27655822377149	0.608768284759931	5.89342603618743	2.43415202089654	1.55160549567154	4.3043664121447	4.46142487535092	0.790747875859635	
**MM122**	4.25901594278443	0.566115402297038	7.39181865951863	2.70713256599136	2.06937690682328	5.84481822233519	5.68854189182071	0.227298612758527	
**MM123**	4.19602287823804	1.03046459618432	6.32773775860178	2.66973367398856	2.15029741467583	4.88369231772388	4.75130588339575	2.33323116684258	
**MM126**	4.66313162247036	-0.025836941211662	5.58741078284705	2.19879325942469	1.64340658021384	4.39636737108991	4.39767422111686	1.38376486791805	
**MM128_1**	3.8113138180437	-0.381670072359042	4.77636073465434	1.89306007198588	1.78605731839803	3.44184532156753	4.04593475202124	1.76501409250583	
**MM131**	4.427538451957	0.963452546759765	6.07275239741248	3.55482039944582	1.18759737099792	4.65634930854132	3.74656144265935	1.98211420880669	
**MM132**	4.26798093761769	-1.21740688749993	5.18241318526525	2.17886205527559	1.09385610521263	4.22941323247836	3.9102197982269	1.47755107369363	
**MM133**	3.84781234389272	0.055460366286737	5.58374142929539	2.19010005223114	1.22379985216043	4.50913947675164	4.42401314163852	0.594686571591991	
**MM134**	3.84788627885769	0.671213442390422	6.85887126272318	3.04533838139992	2.49649482107272	5.99804209186832	4.84251948656707	1.65991401896761	
**MM136**	4.91498191259354	-0.313302833259308	5.45492387418694	2.70995855523992	1.2715896113248	3.51978442450457	4.59853115327675	1.28545641449986	
**MM138**	4.36177915832861	0.322907417058925	6.30827320765551	2.30887961798103	0.837376235473863	4.80088958710264	4.51222387627888	1.69782192945266	
**MM139**	3.69891618342426	-0.621928592827983	6.35853735487404	1.88068398352733	0.967164391736219	3.74595449132142	4.18626009544691	1.64636753486262	
**MM140**	4.2168595601178	-0.121122267767305	5.39222345559328	2.05807851863229	0.993479844591587	3.20691044273248	3.91015499647163	0.780630983991152	
**MM026**	3.98173554210544	0.847027784201536	6.49763222795447	3.43479562579737	0.774424938491998	4.26415323841797	4.32062581821245	1.34554546667685	
**MM027**	3.8426614014515	1.26920198540438	6.73163284469243	2.51565129009211	1.44625148874801	6.12371106886393	4.91335955545212	1.62895514717604	
**MM028**	4.61438089212521	0.450155726108167	5.52098467108745	2.68532831731014	2.08909333839722	4.06915677313819	4.2725195441733	1.68980936721148	

Supplementary Table S5. log2(RPKM) expression values for 39 aminopeptidases genes and 17 housekeeping genes in MM patient samples in the FIMM dataset (*n* = 122) (17/21)

**Gene symbol**	***CLTC***	***G6PD***	***GAPDH***	***GUSB***	***HPRT1***	***LDHA***	***PGK1***	***POLR1B***	**Gene symbol**
**Sample_ID**	ENSG00000141367	ENSG00000160211	ENSG00000111640	ENSG00000169919	ENSG00000165704	ENSG00000134333	ENSG00000102144	ENSG00000125630	ENSEMBL ID
**MM030_1**	4.97040132012337	0.274685531145118	6.08193755502452	3.0965078516678	2.36523525761217	7.44151349262842	4.74909270454386	3.06388660826505	
**MM030_2**	4.83561328948955	2.4493208267282	7.77980161589432	3.2276017580656	2.28016722575176	6.63441122524828	6.20648561494381	1.90582200941656	
**MM031_2**	4.143490501021	0.170105942613542	4.59277590051852	2.31277328290075	1.94926984611589	3.52948745208247	4.38746165045903	2.27027914394476	
**MM033_4**	4.23537193500489	1.05719863495121	6.67659662502289	3.06487661309713	1.87191813416689	5.54735593425861	4.61995373683468	2.19961023722098	
**MM033_1**	4.27604532303557	1.68661971906757	8.27873864515566	3.78303441029277	0.795312000344542	5.33032699043863	4.96393666868266	2.26402863933298	
**MM033_2**	4.22267878353104	0.205086386291859	6.55399469540215	3.29216457966914	2.07710518231308	5.46648987439252	4.79082046690232	2.69208655537779	
**MM034_2**	4.56243532853495	2.34889881082723	7.37464672255347	2.62699570713606	2.34282145812906	4.23233757888271	6.15837181547608	0.8530773884124	
**MM034_1**	3.50405013188996	1.00673751307496	6.80596387531931	3.15929590797295	1.60223423913603	5.26708804980468	5.11752500398122	1.14736187217406	
**MM037**	4.10009785896355	-0.235174174926203	6.52319228544146	3.47254749707991	1.36102368481246	3.74174596836736	4.58333142573542	2.45121494317638	
**MM041**	4.49975756022014	0.674478201455994	5.83110568184746	3.32731922226125	1.73828209969825	5.65487804900734	4.1698965559376	3.32973476378023	
**MM042**	4.86842756931005	2.44065194283866	8.98208955384405	3.33808809020649	1.3178662199673	5.78650915436198	4.85223901357889	2.2806852214406	
**MM043_1**	5.18431406229588	1.25478979223703	6.19291113251409	2.84282696764433	3.37223218041991	3.81508473720977	4.19381354452302	2.24905831415498	
**MM043_2**	5.34981787024772	2.18998713132832	7.82965879116016	3.80720182990247	4.22282391932334	6.38217292084857	5.27153220746017	2.65346902925762	
**MM044**	5.52152369924561	2.23968008306141	7.81561674640786	3.11101743647453	2.94520148861446	4.25344781039844	4.87037562323093	1.54314429253415	
**MM045**	3.88917725001427	0.803843550404743	6.47969183958792	3.34837215006605	1.65912837778396	5.74366808364034	4.91222700816645	1.64948371835413	
**MM046_1**	4.93125880507255	2.28421677519589	6.73506699679149	3.39307617483187	2.37942627372594	5.80637912488745	5.02391468845326	2.41393817711773	
**MM046_2**	4.48239554011927	2.43425354942948	6.70878914506313	3.18343637478904	2.57595500026036	5.88730616461326	5.20034339031354	2.37222066229763	
**MM048_2**	3.97676124020311	-1.69334034108589	6.51418456530397	3.11277719354831	1.33762370093137	5.5329893144962	3.62891565519946	1.4788063098265	
**MM050**	4.39000202614197	-0.177699173575922	5.72162242648607	3.55092997001289	1.89286071590111	7.21070898989935	4.82483470564306	0.415518354214209	
**MM051_2**	4.30962330145369	0.470844480186994	7.09866795026496	3.65136070756451	2.36609940338906	6.33004343627277	5.18392971340088	0.807558927579362	
**MM054**	3.43339643022819	2.20357965464411	7.27818222455699	3.03645360569084	-0.796978677615867	5.78143701434531	4.76023236596525	1.52522992919452	
**MM056_2**	3.47046605857499	0.181114607971161	7.63379315919949	4.11078633914343	-0.172450247162368	5.4730844305375	5.03178545944195	2.81913867975618	
**MM057_4**	4.84758709794288	1.7924273987591	7.02263327752277	2.58228990758474	3.01437322967704	5.70925273909927	4.85020677317248	3.17253669461504	
**MM057_5**	4.60334441150489	0.248197538892936	6.82421487630913	2.04940704105395	2.52763356091225	5.22099498398657	5.1536217229112	2.61314210275852	
**MM057_6**	5.02409472679819	0.324833526117456	6.7387634419905	2.54723229997027	2.34933418228337	5.1711982017033	5.28515524506702	3.63764230575181	
**MM057_1**	4.41938801974298	2.29441151100395	6.7734341460005	2.58377975207915	2.51964911493808	5.91867877915564	4.47376079466723	3.25750412440194	
**MM057_2**	4.45710002696083	1.83416152470717	7.5105408969275	2.87409762386159	2.05589560220717	6.80866047943397	5.6001159060631	1.73453283329223	
**MM057_3**	3.87653379655725	0.752478053630457	6.47105249611531	1.7436042511847	1.69490807079283	5.75760595445824	5.02351019064968	3.35145223830369	
**MM058_1**	5.9148209987423	-0.483570556713588	6.83555813643244	2.00149021678571	2.39172396625169	5.9273273837455	6.40232232820264	2.62431401343939	
**MM059**	4.61847233705841	-0.552394824981332	6.50200263699635	3.30676390714724	1.10601429388935	5.17190728138379	4.63803079016041	0.526185492736325	
**MM060**	4.54028229430157	-0.181410299575036	7.44662812298055	2.87688067779562	2.47097284377897	5.74366963118419	4.87170993565392	1.94845181739903	
**MM061_1**	3.36744310816711	0.328673923467453	6.83883994328962	1.96821621609155	2.0771434048114	4.83134188545801	4.68748414752695	1.58570059509097	
**MM068_1**	3.54068425620886	1.79429036395024	6.28920127134728	3.05244110401149	3.03593888924034	4.0608154734796	4.58531571831376	1.35152311737846	
**MM068_2**	3.86263238610444	0.091209894724295	5.6690006512948	2.88582595556012	3.27871398442523	3.28166322840574	4.67084038147046	1.74839604095021	
**MM091**	4.4229150512461	-1.0104111748922	6.42559707080499	2.87636009969393	2.19054240879343	5.89154742134852	5.09529911201267	1.62547768958181	
**MM095**	4.39832412788826	0.827588167947557	6.2438845992239	2.93041405973222	1.24364360415019	4.48760757814574	4.53697515407408	2.15554391039015	
**MM002_1**	2.19786099263147	-0.510709371358384	5.52448209047537	1.52429521113013	-0.285236348717059	3.63681681320851	4.19144680655496	0.012899636219775	
**MM002_2**	3.04895516697352	0.062914958266112	5.79166242993951	1.58266240023087	0.571888793644336	4.49747933643685	4.2724177180218	0.252980744755144	
**MM002_3**	2.72276811394357	-0.705245099900101	5.61634804834394	1.7391027730721	-0.798871128521137	4.36479673290479	4.42180922450845	-0.123798076649332	
**MM003**	5.42878484641981	0.533279748148647	9.67928149843731	4.04835204862723	2.71898691661062	6.43433846710253	5.00588535048657	2.95289015698566	
**MM005**	4.56677484547648	-0.451412303349517	8.12492069170455	2.61100088693998	2.95935358521185	6.26614592603911	5.59185160019719	2.81935142989636	
**MM007**	4.856930924241	-0.17544574644098	6.92306971032813	3.65030321925718	2.48599454498771	5.33824207041738	4.8067766179319	2.1172193160763	
**MM010**	4.15674695372054	1.13268788407331	6.67703086152533	2.36301370422809	2.53425854211225	6.13048358495769	5.31807475465345	3.2716866593025	
**MM011**	4.84603636208376	0.971450408400692	7.78505060245317	3.70014799074511	3.34686563171209	6.32896811942452	5.20731021279771	2.94513948500665	

Supplementary Table S5. log2(RPKM) expression values for 39 aminopeptidases genes and 17 housekeeping genes in MM patient samples in the FIMM dataset (*n* = 122) (18/21)

**Gene symbol**	***CLTC***	***G6PD***	***GAPDH***	***GUSB***	***HPRT1***	***LDHA***	***PGK1***	***POLR1B***	**Gene symbol**
**Sample_ID**	ENSG00000141367	ENSG00000160211	ENSG00000111640	ENSG00000169919	ENSG00000165704	ENSG00000134333	ENSG00000102144	ENSG00000125630	ENSEMBL ID
**MM014_4**	6.00826923092059	2.1888156086748	8.76003540628596	3.82988854876644	2.16258518880896	6.74265920055548	5.42914943483858	2.27161971498468	
**MM014_2**	4.79716519683748	-0.325417493504677	6.22167776349451	3.53932332835281	0.954660573664792	3.87873918751167	4.31087338244672	1.87636143806693	
**MM017_1**	4.24785340630977	-0.758534174628053	7.51670164995187	2.66176905506923	1.25848651641703	5.71432722649911	5.20124670026933	2.72876774022813	
**MM017_2**	4.13479091010972	-0.173380444515377	7.1158876195619	4.0813235797736	1.96096298897556	5.23285222708828	4.45601371317828	2.3168741199317	
**MM017_3**	5.39149177167302	0.705545390455784	8.45011289662893	5.23013733747335	3.46847804726936	6.40952399785066	5.31108369954671	3.07066468216105	
**MM018**	3.98720932273856	0.545100708953911	6.3552610525513	2.07245426971855	1.29026411528701	5.09779093555044	4.23862631905614	1.60800914722652	
**MM019**	3.17624760662359	-0.431501031953581	6.31833741928458	2.70104821299531	1.13312467323947	5.11758191705672	4.47294423202799	2.15653443932377	
**MM020**	5.15982061717246	0.548097314127702	6.47289026495377	2.31630462542108	2.29024470452345	5.94692837414735	4.84978081146901	4.03822515289655	
**MM022_1**	4.82893416518426	2.37374568139121	7.40001435769109	3.23423916196581	-0.11422274233926	3.71699233179522	5.35750052758792	2.25237382241438	
**MM022_3**	4.15280219838149	-0.14965105406922	5.93882341038703	2.88164759177908	1.5236295446001	3.45996044879843	5.21254488212545	1.03388988766834	
**MM024**	4.04049194495047	1.56091197193452	5.68496886395581	1.86634627490998	2.95783099185415	5.45199800897016	4.58078647705321	2.50918016180558	
**MM025_1**	4.10072720112481	0.44545962398214	5.73424404522884	1.87330020219242	1.1311595971893	4.22145027593591	4.54032770909752	2.4405423030292	
**MM036_1**	5.8708707309175	-0.105637263682061	6.13004937932942	2.70253950853728	-0.402153567783076	4.72044662524349	4.61958893653227	1.95816621448787	
**MM036_2**	6.02572091113445	-0.753342943977757	6.17985873394732	2.56556740144756	0.483489368459803	5.17345701170933	4.75845295295811	0.952575181955741	
**MM038**	4.75796449034511	0.112965270047463	6.61188672548743	3.79239994582023	1.45710780727685	5.23006397929067	4.81013084142086	1.92367188077099	
**MM039**	3.76469611775135	1.25569771664015	6.24208598971567	2.99839545205524	2.50089362349449	5.87039324587782	4.88707288944669	1.3093066757374	
**MM049**	3.75816415150071	-0.305074071884597	5.57233744452128	2.63731515864301	0.962730197823257	4.61211856885008	3.68337866917076	1.67058704420495	
**MM071**	3.83734487907973	0.299099612179195	6.41071347166147	3.46166190128595	2.33857515405198	5.70935293003479	4.18050580426071	1.79914710732081	
**MM080**	3.98376094907497	0.872704484804242	6.6951239543591	3.02402365731639	2.02720584548152	5.18823725375046	4.49637242031059	2.30412780895688	
**MM081**	4.80065423895581	0.743827369668343	7.54640816738637	3.28634670345557	2.16495424232542	4.87354820482125	4.24857114457305	1.62572805481422	
**MM082**	2.75704527711992	-0.839833365306427	6.2244599856311	3.0178247755269	0.758028303979711	4.58432187249377	4.32454438041371	1.65756489696824	
**MM098**	4.97351229367519	1.83116174564717	7.68860857374726	3.29913987809625	1.84441378219295	4.71744899977853	5.32184770268752	1.37613854428427	
**MM102**	4.45336957266564	2.7412126947685	7.57098254155881	2.54493795279726	2.6093263401882	4.59853218242486	5.83718889898884	0.887881532162242	
**MM109**	3.96348402240903	0.055955208745002	8.68686178403211	2.73102157326981	2.28991977659479	5.75166981851331	4.86984819398511	1.22086874304012	
**MM111_1**	5.16667784650448	-1.93856115586615	7.68516905389482	3.06821123526461	1.81891889108844	5.48403313994573	5.80033453903838	1.9085894295498	
**MM111_2**	5.79289212642987	0.028367919957738	7.5928957548091	3.12353307958839	3.3067272186233	6.02630889603765	5.492328809476	2.97721551428408	
**MM113_2**	4.75843305358724	1.54750970917293	7.28880790474057	3.24871477342527	3.26581509414918	5.09296625834442	4.58194510211537	1.89839763120288	
**MM117_2**	4.53584055494412	0.098367473091851	7.64152717336743	3.22322639134351	1.56065005585612	5.06751748488328	5.39585132289528	2.66690823674988	
**MM124**	4.2688071959145	-0.801342783947263	6.33407007097977	2.72567056890114	1.04699805141413	3.90587841888988	4.6266077672567	2.28836523263713	
**MM129**	5.10911908989663	1.3654532750757	8.4064316395097	3.77559887933476	3.44029118895461	6.15930723374584	5.37511183875796	2.54357368285167	
**MM135**	4.32960824750849	1.50752315706818	6.20634597642055	3.95665112783223	2.87496892923515	5.81839392466397	4.97423101289258	3.17722087264084	
**MM137**	4.42073188622783	-1.73931000451698	6.6012891985506	2.67042643303305	0.631398801593659	5.58440698216303	4.88176371019525	0.536099517287961	
**MM006**	4.11830454081655	0.693958069983074	6.45302804444186	3.07205343128222	1.66551287742959	6.01022238339648	5.31136490677694	1.57629984946867	
**MM008**	4.31716110925626	0.755991957836452	6.58060831925686	2.74769089079549	1.86788774545325	4.88146434473269	4.90693658241904	2.14315943638363	

Supplementary Table S5. log2(RPKM) expression values for 39 aminopeptidases genes and 17 housekeeping genes in MM patient samples in the FIMM dataset (*n* = 122) (19/21)

	Housekeeping genes						
**Gene symbol**	***POLR2A***	***RPL19***	***RPLP0***	***SDHA***	***TBP***	***TUBB***	**Gene symbol**
**Sample_ID**	ENSG00000181222	ENSG00000108298	ENSG00000089157	ENSG00000073578	ENSG00000112592	ENSG00000196230	ENSEMBL ID
**MM032_1**	1.90900095607337	8.01912086028781	7.58666575874774	3.04989107158624	1.2607172557548	4.37900061296906	
**MM051_1**	3.8492461964355	6.8187328697066	6.95174539221958	3.49921588000285	2.25927513975302	4.43077328893564	
**MM053**	2.64959465127335	6.61038664314342	7.72032844852403	3.53427901111252	0.47664165055086	4.49809305078788	
**MM056_1**	2.17302085159129	10.0200148397369	9.61484195313039	2.98000501098375	2.35189298579644	5.08271856362828	
**MM063**	1.35406869313452	8.43570309810838	8.30320795724813	2.9962679009942	2.09076742339479	4.08866299330981	
**MM064**	2.90167069150736	8.39701419898563	7.47119345899687	4.45923322437565	1.13639468313514	6.03700050254277	
**MM065**	3.06702435627096	7.84475467508515	7.6709551918984	2.2750934734355	1.78728188209462	5.37948919925405	
**MM066**	3.01230916561483	8.78683168653478	8.25798096061955	3.25357722425857	2.43429872323257	4.56298593796764	
**MM067**	1.74270407742102	9.40083460872123	8.60730504221437	3.15910345405228	1.46535353974454	4.39172257465135	
**MM070**	1.61639320319801	9.20446743130581	7.89270821832674	3.62509216252702	1.51475504390312	4.74502946057672	
**MM074**	2.40225361505146	7.70075927187522	6.49019919485002	2.76573719617256	1.81598006295875	5.27002017442633	
**MM075**	1.27886185677649	8.94034235380439	8.53882130235999	3.52336653667091	0.524395016399111	5.42840908127286	
**MM078_1**	1.18658464062673	8.10894477402634	8.04801514896737	2.32152940992321	0.903306771279101	5.66895244600696	
**MM085**	0.911957167979301	8.19431915637134	7.77946735681916	2.87035539305314	0.744829381017355	4.41230397535469	
**MM086**	2.45186417289643	8.86261533781989	7.321862590333	3.49372763247629	1.16301756599366	5.33783021238866	
**MM087**	0.862618261112554	9.1617489670797	8.90499964490951	3.66067706291003	2.01249186355465	6.52234729027794	
**MM088**	1.26485787218388	9.14404997712199	7.70110197651151	1.59062867695349	0.504444692980416	4.98576849765965	
**MM089**	2.50411617836488	7.6627935804627	6.93502045995375	2.66639094061892	2.30699390281981	4.32507105382165	
**MM090**	1.72975247306378	8.93288358716817	8.22632622780014	3.91931188209067	2.76495986908792	6.15897538977217	
**MM093**	2.08665737453788	7.98271282883804	7.87596477018174	4.07908144641917	2.05168345858433	4.47528656242697	
**MM094**	3.59367442849111	8.95723433620636	8.21085567484776	3.74161520930371	2.36677292957016	5.75900772636311	
**MM096**	2.27524144382496	9.06716358347345	8.53235775533834	3.00469270632032	2.48322164939049	6.1900280807698	
**MM101**	2.97669100374644	8.50253937927236	8.52024394008776	3.04239441738945	0.910079377068534	4.75061202978275	
**MM114**	2.41596400807935	7.72827034241688	7.21845753295249	2.94302346128991	1.85100225803676	3.89139299563257	
**MM115**	2.57650826278182	8.64027564669896	8.06258867624572	4.43790743954632	2.40806435001025	5.78142447822787	
**MM117_1**	0.765317239091839	8.19858965086398	8.26188621852713	2.87230994272504	1.06959592230219	3.55474562023211	
**MM118**	3.53518975716016	7.85763626235658	7.41249226449369	2.97384482347379	1.58250639161435	4.50323472762452	
**MM119**	2.15687848418234	8.39808710228579	8.391645122471	3.32625965944125	2.23070184656244	5.23618587868095	
**MM121**	3.28869977424752	8.804448152499	7.36841381952831	2.8776216324057	2.02639620156748	4.19752234018723	
**MM122**	2.44354308331539	8.87465096963633	7.61965497506705	3.25998001588879	1.23129650123017	5.08316456687439	
**MM123**	4.20389063064923	8.63358016991036	7.53203892200444	3.40370579906592	2.14255569824279	4.05708684441954	
**MM126**	2.84607778910956	8.45585571009918	7.305233201893	3.30325473041938	1.39920747550702	4.142986408249	
**MM128_1**	-0.293883753918095	8.54052928321	7.82448691459803	3.47544292410579	1.56087867969614	4.88382860072052	
**MM131**	2.14325591869904	9.15406464610409	8.14756104265748	3.83423542053561	1.62078870927638	5.02435108109859	
**MM132**	1.71400638060732	7.610349655217	6.90681468114767	2.12186587488227	0.284263336531058	3.36472129643904	
**MM133**	2.26424464337037	8.84155461155343	7.9948347038595	2.25402195126976	1.26832524071968	2.73972644765208	
**MM134**	2.01646271386077	9.04440675389781	8.80362975084708	3.34035533199886	0.792390803602216	4.97539763095894	
**MM136**	2.30327951984937	8.45597150430181	8.0658716009155	3.73121295359784	2.32249881559789	4.28292744131157	
**MM138**	1.48195898562344	8.18868310701751	7.72691112611972	3.56471850648547	1.96710809318136	4.28012886934491	
**MM139**	1.13578368308192	9.33945955048045	7.63520624218025	2.86622397909166	1.83425379832375	3.82160634199378	
**MM140**	2.57439195797568	8.36230633829054	7.08197903072186	3.05255629493078	1.71042108578746	4.26766785198908	
**MM026**	2.281105459904	9.8603922075498	8.45707037892302	3.48635351327249	1.07534657805753	4.36510071735535	
**MM027**	3.59036673884597	8.68883623245867	7.6530868736335	3.23396558629432	2.2003974860987	6.22056589091348	
**MM028**	2.03479232464126	8.08616056435995	7.00133972989625	3.21866804855371	1.86234235911885	5.81597308666497	

Supplementary Table S5. log2(RPKM) expression values for 39 aminopeptidases genes and 17 housekeeping genes in MM patient samples in the FIMM dataset (n = 122) (20/21)

Gene symbol	*POLR2A*	*RPL19*	*RPLP0*	*SDHA*	*TBP*	*TUBB*	Gene symbol
Sample_ID	ENSG00000181222	ENSG00000108298	ENSG00000089157	ENSG00000073578	ENSG00000112592	ENSG00000196230	ENSEMBL ID
**MM030_1**	3.78989802417277	9.25316540271148	8.51939886241036	2.66279840640868	2.51478632768757	6.45596039986878	
**MM030_2**	2.52363975764723	9.88639947239267	8.90993564074822	2.02924942010269	1.27105505937392	5.16094813936295	
**MM031_2**	0.771882230772295	9.53434532726665	8.48012039275091	2.91701620313056	0.853707192321073	3.41158673233167	
**MM033_4**	2.75018915098208	8.46617716908429	8.36838454771905	2.83068408123495	1.32088799058264	5.65549253524473	
**MM033_1**	3.85749162792095	8.03926611192682	9.33136533818616	2.87639201391992	1.0322969085176	5.37859165382442	
**MM033_2**	1.29589141263246	8.81579676570343	8.6846987866685	2.56920125062199	0.545263397364794	5.41728425931318	
**MM034_2**	1.71955988087472	8.99718535030914	7.72498243868674	2.86892956807981	1.17843507174135	3.87482645011503	
**MM034_1**	1.12366329444694	9.25385616560794	8.24821127145071	3.86579345308786	1.37877226167116	5.48936086325012	
**MM037**	0.398109283186805	10.0671801687197	8.68811770210036	2.47962240801044	0.483446064670239	4.16730723033172	
**MM041**	2.74838226882433	9.10188537608574	9.1648829679177	3.66403400368067	1.74910456684699	4.97703670795239	
**MM042**	1.8996633587772	7.48520113307901	8.58638473626463	3.277124620513	1.21402047305445	7.23842785262611	
**MM043_1**	3.4500562709878	8.34152271227439	7.25594672551852	3.0668726998011	3.07846926149181	4.24993914019989	
**MM043_2**	3.4906456275464	8.45824541616592	7.85358333794136	3.49100626751206	2.95023560234718	6.36051395951662	
**MM044**	2.90429011796739	6.2033105318251	7.99738600014179	3.76182617676891	1.85943532771668	8.16797643051646	
**MM045**	3.20971287568049	8.99491773516592	7.63344666883004	3.50196400740896	2.56698259408759	5.56999877365899	
**MM046_1**	2.89737210938512	8.78746741145171	8.48679722629077	4.64357315784998	2.39473295911621	6.00770521411512	
**MM046_2**	3.23440698255475	8.7884247214019	8.190124566159	4.28344448260221	2.59733023623752	5.76365613988767	
**MM048_2**	2.42791945686625	9.3063603251597	9.06560534959236	3.63958381506686	0.87904772820763	4.00871799976141	
**MM050**	2.18757922652317	7.9247311401757	7.94565031888153	3.8158508353517	1.57005529233217	6.27382649326569	
**MM051_2**	2.97738867351261	7.90007703937695	7.26739914229014	4.1728743067525	2.72604212682923	5.48661132877307	
**MM054**	1.83902649550303	5.28789764315264	7.27578148349077	2.31466858500632	-0.541615405939295	4.92667431923033	
**MM056_2**	2.12096271758042	9.29989580337225	9.22935732769969	3.36450848150118	2.11046137262099	5.77080689830108	
**MM057_4**	4.47146881361515	9.38926435153843	8.73922810340221	3.67637219945947	2.67668640063061	6.25391794391583	
**MM057_5**	2.91810353521838	9.84149954336098	9.4931064654659	3.09713820221519	1.67077165221496	5.2956420056352	
**MM057_6**	1.96666897016429	10.2770628459855	9.81573420406992	3.04015926549641	1.30686811021665	5.15895418243518	
**MM057_1**	4.42492185893251	9.17073223529784	8.21143070946375	3.95372265242291	2.84312234645619	6.87359043120057	
**MM057_2**	2.82795791094961	8.67103634945808	8.06463446889957	2.82628362756272	2.45708695047206	6.04001353037873	
**MM057_3**	1.92629007794272	9.85305802475474	8.90416556769228	3.20354187983774	2.02544300354923	6.13525742512806	
**MM058_1**	1.68613844920371	7.96400937093645	7.10785721716567	4.05714368040736	2.24356932283187	6.30291247867981	
**MM059**	1.69681999297292	8.07409022754385	7.81634493638926	3.99311263314629	1.93154474779263	4.7832702621166	
**MM060**	2.30739963829892	8.89686599295441	8.72406103835558	3.47392724946862	0.864943968547557	5.15450112033208	
**MM061_1**	1.12389960389187	8.49494603589276	9.04506704080369	3.00237280277353	1.5276773922708	4.25962143227548	
**MM068_1**	1.74878464255679	7.07226635263154	6.63118452411406	2.54924171224221	1.77426842987175	5.20451832579478	
**MM068_2**	0.665257118104722	7.43862171875579	6.70291804135841	3.17435220122085	1.33473974570773	5.29034924595933	
**MM091**	3.64612227221585	9.17791451872469	9.00065710436566	3.93136923112079	1.06043772971648	5.56898572698073	
**MM095**	3.23880943399936	8.19849193052078	6.21749147476846	3.68551366261973	2.80302834293212	5.14509502486602	
**MM002_1**	0.593212113030711	8.77262045639181	8.0452912011943	3.14861679910989	-0.179147585087165	3.42730639775233	
**MM002_2**	1.90315915723045	8.74888255745188	7.94938374478304	3.00436545302191	-0.489300541047538	3.29442971837495	
**MM002_3**	1.1659912840194	9.3310308837056	8.13121601997696	2.94978779567097	-0.241145195052721	2.61752016155635	
**MM003**	4.08202899251779	10.0387869493598	9.73605327274442	4.11988964798484	2.46434716280712	6.04351598449035	
**MM005**	1.38405236218555	9.56210781606065	9.12961159384536	3.59837287542544	1.16669601028516	6.60095418450896	
**MM007**	2.29744625414326	8.25272929720772	7.60554239080334	3.99503263759939	1.75454810493725	6.24744692669507	
**MM010**	0.875542147361046	8.92924452370081	8.63530535343771	3.6545506902012	1.23561015005268	6.84262665382766	
**MM011**	4.11207587007349	9.00943796153334	8.14348989243081	3.72689514225823	1.87542262946654	7.34328564144541	

Supplementary Table S5. log2(RPKM) expression values for 39 aminopeptidases genes and 17 housekeeping genes in MM patient samples in the FIMM dataset (*n* = 122) (21/21)

**Gene symbol**	***POLR2A***	***RPL19***	***RPLP0***	***SDHA***	***TBP***	***TUBB***	**Gene symbol**
**Sample_ID**	ENSG00000181222	ENSG00000108298	ENSG00000089157	ENSG00000073578	ENSG00000112592	ENSG00000196230	ENSEMBL ID
**MM014_4**	2.15104210977924	8.90254820463119	8.23911514726065	2.94260822590478	2.35348845542621	6.26165355742324	
**MM014_2**	1.18141324626899	8.08653652236156	7.72808808299038	3.14059585633639	2.18196342154913	5.09801834306724	
**MM017_1**	0.806755037193914	9.19952751172893	8.86872893533964	3.76178669211146	1.57570385746867	5.43366108560881	
**MM017_2**	1.67371397522009	8.92727350580001	8.4456364333575	4.12616837198891	2.66835013094186	5.66304669639417	
**MM017_3**	1.41488840149847	9.29022485947528	9.31502795845421	3.14170447043176	2.08035993739726	7.56938176578953	
**MM018**	3.96888427648939	7.40342396784616	6.26335114562256	2.7854215929855	1.96158945914004	3.42592981836512	
**MM019**	1.35868771202538	9.26348968657011	10.244311717331	2.39419368167186	1.22345488274965	4.07572529461706	
**MM020**	2.7958064764161	7.790926590116	7.79603164586767	3.96455733257601	2.44021201682458	5.5996220627444	
**MM022_1**	3.8111286125794	5.48662915564099	6.87498710843293	3.73379965994635	2.7220666058582	6.45645794758514	
**MM022_3**	2.29513430137963	7.55598370987185	7.02103832298757	2.97844927591342	2.60765802345506	3.95168362625985	
**MM024**	3.00655752446156	8.7975246237062	8.52632734402746	3.43929413189392	2.15937077334445	6.11547705554057	
**MM025_1**	1.60081186608789	8.08882436231893	7.38406580402508	3.39351876564318	1.38798820165695	4.91716270638039	
**MM036_1**	0.04643375099583	8.76052842275904	8.77326011824809	3.23243394250655	0.145605060108743	5.29394843723068	
**MM036_2**	1.29122056162696	8.55784262625174	8.62098561251734	3.02794256358101	1.18762614054691	5.17592841712559	
**MM038**	0.09987713355929	9.2261095675272	8.31878918787866	4.09433563273378	1.24975637835224	5.13165457222962	
**MM039**	2.26049291237907	9.01630848653198	9.051481155666	3.31474488851785	1.90099949642743	5.52253043757956	
**MM049**	2.68073403273831	8.47967407890119	7.74292105416314	3.82223930933384	1.67522113032486	4.11216707393963	
**MM071**	2.1470443015965	8.83081582966656	8.83467482527321	3.24052944694955	2.18432073091475	4.7684374376153	
**MM080**	2.74132595524378	9.05530414679534	8.16409925181928	3.23648503709234	1.46478403588031	5.40988180200625	
**MM081**	3.98041692249645	7.93458219423869	7.76251516488052	3.75848824844051	1.51421695712743	4.90235099716045	
**MM082**	-0.130807504484237	9.50156323439052	8.67473876096513	3.68281533543818	0.248603109049927	4.20982558931692	
**MM098**	2.57270452782263	8.48017261342904	8.23289126921394	3.74666409475821	2.33651660847731	4.48276897613452	
**MM102**	1.22104789580576	7.78789338357756	6.87297227718333	3.08512119579868	1.10375572409374	4.91411148512464	
**MM109**	2.86960474572783	8.64391897687295	8.61510892127015	3.50498575055372	1.52714978654303	4.17095834627592	
**MM111_1**	2.24248847486738	9.07925141162133	9.4301394654079	4.0298251631705	2.04411263618259	5.19385470762953	
**MM111_2**	3.17685612863811	8.30827060668739	8.17750504324074	3.71045838278564	2.41658027806459	5.3472709736665	
**MM113_2**	2.67041650656575	9.69772942950021	8.40270665284482	3.85442092539694	2.3286139051096	7.34119534902446	
**MM117_2**	1.17488208542064	9.02500585657412	8.88119104259046	3.96738118335618	1.45871965735303	5.22775938593236	
**MM124**	1.42771177831129	9.0074994607261	9.09634273496478	4.22020834926785	1.63671487380315	4.21410948487979	
**MM129**	3.60950754401011	8.95889066740617	8.89846703101617	3.30403395576895	2.09572848222912	5.97724181130943	
**MM135**	2.00082014692194	9.80023969500385	9.53235268823632	5.71740247425088	2.2304239230194	4.8024765587515	
**MM137**	1.02947256582855	8.36435946629741	8.25659489023437	3.65658341533633	1.24262625992648	3.71241364506295	
**MM006**	0.023770864615895	8.32528047710918	7.83550534887312	3.17650208539535	0.681090325719764	4.79518323655455	
**MM008**	3.57101083816457	8.35650024891247	7.31182765440597	3.35559335247453	2.47991270849313	4.4152487870682	

Supplementary Table S6. LC-MS/MS-based proteomics label free quantitation intensity values for 17 aminopeptidase proteins in CD138+ cells isolated from MM patient samples in the FIMM dataset (*n* = 23) (1/2)

Gene symbol	ERAP1	BLMH	DNPEP	DPP3	ERAP2	LNPEP	METAP1	METAP2	NPEPPS	PEPD	
Protein name	Endoplasmic reticulum aminopeptidase 1	Bleomycin hydrolase	Aspartyl aminopeptidase	Dipeptidyl peptidase 3	Endoplasmic reticulum aminopeptidase 2	Leucyl-cystinyl aminopeptidase	Methionine aminopeptidase 1	Methionine aminopeptidase 2	Puromycin-sensitive aminopeptidase	Xaa-Pro dipeptidase	
Sample_ID	Q9NZ08	Q13867	Q9ULA0	Q9NY33	Q6P179	Q9UIQ6	P53582	P50579	P55786	P12955	UniProt ID
MM046_1	24.7113	19.2347	21.0844	22.3697	20.006	20.465	20.5704	20.7326	22.8536	22.584	
MM041	23.9385	22.1415	23.5018	23.8521	21.1689	21.0604	21.2797	21.363	23.9005	23.4966	
MM064	25.3169	22.2104	23.5301	23.4929	21.9913	18.7163	19.2722	21.2253	24.1857	23.0849	
MM011	23.6926	21.9358	21.5262	24.3608	21.8068	20.8864	20.5233	21.9245	24.8088	22.4492	
MM057_1	24.8582	22.7023	21.7074	24.3797	18.0237	20.5707	20.7946	21.5296	23.493	24.1283	
MM025_1	24.3473	22.5243	18.3807	25.1295	22.4219	20.7795	18.8189	18.6759	24.5621	23.0364	
MM033_2	21.9064	22.1319	23.3005	23.5231	18.9387	18.4204	21.6164	21.3568	24.4556	22.4273	
MM067	25.7706	22.266	24.0684	23.7447	21.3858	19.1941	18.3418	20.8676	24.0517	23.4918	
MM056_2	24.374	22.3194	18.8799	21.1254	20.4959	18.2471	22.8642	18.9744	23.0628	24.5441	
MM007	25.4574	21.4356	21.563	23.6091	19.0234	20.3902	18.5865	18.3103	24.55	23.3707	
MM078_1	25.5609	21.6251	22.413	23.9888	21.644	18.0796	18.9309	21.0485	24.0345	21.8537	
MM095	24.4312	21.1978	23.2931	22.9839	22.1795	20.856	18.472	19.4447	24.3027	22.5541	
MM066	23.5103	20.807	22.5512	23.0215	21.1538	20.736	19.1762	21.3696	23.8438	21.5221	
MM043_1	23.3683	21.9309	22.0692	23.0482	21.8045	19.2803	19.1309	19.5286	24.0665	19.3847	
MM020	23.9758	21.9205	22.1529	23.9101	21.4102	18.7155	21.6275	18.6846	24.5302	22.6953	
MM032_1	24.2657	19.0407	22.5971	23.243	22.1541	19.2696	18.7976	18.5184	24.4647	22.3936	
MM075	24.3382	21.8921	23.8179	24.0392	21.1998	18.819	21.4561	18.7368	24.3674	22.9608	
MM065	24.339	21.6968	23.4268	23.7799	19.4227	18.2856	20.3726	18.64	24.1898	23.0178	
MM074	24.9367	21.1766	22.4422	23.3359	22.9194	21.071	18.6308	21.7832	24.5299	23.0976	
MM070	24.2423	22.3367	22.2455	23.3762	20.7868	20.5967	18.9516	19.4787	24.6001	22.4085	
MM030_1	26.0273	22.6814	21.8559	22.9622	21.9495	19.2425	19.5261	20.8292	24.5134	22.846	
MM003	25.5683	23.3213	21.3351	22.6769	21.1791	20.379	19.9859	21.6774	24.1771	22.9552	
MM045	25.6829	21.5224	22.5574	22.6386	22.1405	19.0324	19.1486	18.6183	23.5211	21.9902	
	36	10	13	23	14	6	6	7	32	19	Number of peptides identified by LC-MS/MS

Supplementary Table S6. LC-MS/MS-based proteomics label free quantitation intensity values for 17 aminopeptidase proteins in CD138+ cells isolated from MM patient samples in the FIMM dataset (*n* = 23) (2/2)

Gene symbol	TPP1	XPNPEP1	DPP7	LAP3	LTA4H	RNPEP	TPP2	
Protein name	Tripeptidyl-peptidase 1	Xaa-Pro aminopeptidase 1	Dipeptidyl peptidase 2	Cytosol aminopeptidase	Leukotriene A-4 hydrolase	Aminopeptidase B	Tripeptidyl-peptidase 2	
Sample_ID	O14773	Q9NQW7	Q9UHL4	P28838	P09960	Q9H4A4	P29144	UniProt ID
MM046_1	23.0622	20.171	22.85	24.4227	25.3898	21.8087	21.4741	
MM041	23.6453	22.4343	21.8779	25.3869	24.4953	23.0703	23.7875	
MM064	24.0477	20.1249	24.9022	27.0163	24.6414	23.46	22.3764	
MM011	25.6431	22.006	25.2535	26.5284	25.2882	24.8026	22.734	
MM057_1	25.9195	22.0192	23.0519	27.0841	25.0626	20.8396	21.5957	
MM025_1	18.4328	20.417	23.9986	26.8872	25.0051	22.0862	25.1416	
MM033_2	25.0674	22.6355	26.1266	26.4826	25.7746	24.116	22.8114	
MM067	25.6	21.1613	27.3153	25.7634	25.9971	21.7843	22.3861	
MM056_2	26.313	20.1312	26.4802	27.1543	23.3142	21.6254	22.1818	
MM007	24.861	21.5761	25.0151	26.9612	25.4009	23.8632	21.1166	
MM078_1	23.9489	22.6483	22.9974	26.4638	27.1167	22.9193	23.7535	
MM095	23.6781	21.2209	23.3457	25.8649	25.3445	21.6841	21.4192	
MM066	24.6167	22.175	22.4417	25.9771	26.668	23.0343	23.4504	
MM043_1	23.9473	21.2961	26.1497	24.4172	25.6131	21.3401	22.2604	
MM020	24.5649	21.6766	26.4359	25.5937	25.2572	23.1864	23.0649	
MM032_1	24.0167	21.8801	25.1472	25.352	26.1311	21.8363	23.7747	
MM075	23.5902	20.9511	24.5484	26.2928	26.2512	22.6886	22.7326	
MM065	25.6811	22.1933	25.7032	25.2284	25.5199	24.9619	23.3307	
MM074	25.8433	22.3259	25.9252	25.7135	26.5439	21.6282	22.8011	
MM070	24.4003	22.4203	18.6387	24.1388	24.8943	21.4445	24.0693	
MM030_1	23.5602	21.3704	24.0748	25.3158	24.2887	22.4201	22.1088	
MM003	23.3209	21.9291	21.1244	24.2693	24.7248	23.1958	22.5174	
MM045	23.8452	21.0479	25.6059	26.0471	25.901	22.1177	21.9017	
	10	12	21	30	25	24	32	Number of peptides identified by LC-MS/MS

Supplementary Table S7. Statistical overview of genes identified as prognostic markers (P ≤0.05) in the FIMM dataset (*n* = 122).

Gene	*p*-value	Median overall survival, months		Hazard ratio (95% CL)
		High expression (95% CL)	Low expression (95% CL)	
XPNPEP1	0.00012	55 (49-96)	122 (94-NA)	3.263 (1.749-6.087)
DNPEP	0.0056	68 (55-100)	127 (124-NA)	2.197 (1.215-3.975)
DPP4	0.0061	122 (94-142)	74 (55-105)	0.455 (0.261-0.796)
RNPEP	0.0062	73 (49-111)	100 (94-NA)	2.271 (1.248-4.129)
LAP3	0.0085	73 (55-120)	105 (89-NA)	2.091 (1.195-3.66)
TPP2	0.014	55 (55-96)	122 (100-142)	1.916 (1.118-3.281)
NPEPL1	0.017	76 (55-105)	124 (94-127)	1.836 (1.078-3.129)
METAP2	0.019	73 (55-96)	122 (100-142)	1.857 (1.09-3.162)
DPP3	0.02	73 (55-100)	120 (105-NA)	1.956 (1.108-3.453)
BLMH	0.037	68 (55-105)	122 (94-NA)	1.815 (1.035-3.184)
XPNPEP3	0.046	76 (55-111)	122 (74-NA)	1.788 (0.989-3.233)

CL: confidence limit; NA: not applicable

Supplementary Table S8. Aminopeptidase gene somatic mutation frequencies from MM patient samples in the FIMM dataset (*n*=169) (1/12)

SampleID	*LVRN*	*BLMH*	*DPP3*	*DPP4*	*DPP7*	*DPP8*	*DPP9*	*ENPEP*	*ERAP1*	*F11*	*LAP3*	*LNPEP*	*METAP2*	*NAALADL1*	*NPEPL1*
MM026	1	0	0	0	0	0	0	0	0	0	0	0	0	0	0
MM033_4	0	0	0	0	0	0	0	0	0	0	0	0	0	0	0
MM001	0	0	0	0	0	0	0	0	0	0	0	2	0	0	0
MM034_1	1	0	0	0	0	0	0	0	0	0	0	0	0	0	0
MM037	0	0	0	0	0	0	0	0	0	0	1	0	0	0	0
MM041	0	0	0	0	0	0	0	0	1	0	0	0	0	0	0
MM042	0	1	0	0	0	0	0	1	0	0	0	0	0	0	1
MM043_2	0	0	0	0	0	0	0	0	0	0	0	0	0	0	0
MM048_1	0	1	0	0	0	0	0	0	0	0	0	0	0	0	0
MM048_2	0	1	0	0	0	0	0	0	0	0	0	0	0	0	0
MM060	0	0	0	0	0	0	0	0	0	0	0	0	0	0	0
MM063	0	0	0	0	0	0	0	0	0	0	0	0	0	0	0
MM064	0	0	0	0	0	0	0	0	0	0	1	0	0	1	0
MM065	1	0	0	0	0	0	0	0	0	0	0	0	0	0	0
MM068_2	0	0	0	0	0	0	0	1	0	0	0	0	0	0	0
MM077	0	0	0	0	0	0	0	0	0	1	0	0	0	0	0
MM078_1	0	0	0	1	0	0	0	0	0	0	0	0	0	0	0
MM078_2	0	0	0	0	0	1	0	0	0	0	0	0	0	0	0
MM086	0	0	0	0	0	0	0	0	0	0	0	0	1	0	0
MM096	0	0	1	0	0	0	0	0	0	0	0	0	0	0	1
MM005	0	0	0	0	0	0	1	0	0	0	0	0	0	0	0
MM007	0	0	0	0	0	0	0	0	0	0	0	0	0	0	0
MM010	0	0	0	0	0	0	0	0	0	0	0	0	0	0	0
MM017_2	0	0	0	0	0	0	0	0	0	0	0	0	0	0	0
MM025_1	0	0	0	0	0	0	0	0	0	0	0	0	0	0	0
MM025_2	0	0	0	0	0	0	0	0	0	0	0	0	0	0	0
MM055	0	0	0	0	0	0	0	0	0	0	0	0	0	0	0
MM098	0	0	0	0	0	0	0	0	0	0	0	0	0	0	0
MM101	0	0	0	1	0	0	0	0	0	0	0	0	0	0	0
MM103	0	0	0	0	0	0	0	0	1	0	0	0	0	0	0
MM104	0	0	0	0	0	0	0	0	0	0	0	0	0	0	0
MM107	0	0	0	0	0	0	0	0	0	0	0	0	0	0	0

Supplementary Table S8. Aminopeptidase gene somatic mutation frequencies from MM patient samples in the FIMM dataset (*n*=169) (2/12)

SampleID	*LVRN*	*BLMH*	*DPP3*	*DPP4*	*DPP7*	*DPP8*	*DPP9*	*ENPEP*	*ERAP1*	*F11*	*LAP3*	*LNPEP*	*METAP2*	*NAALADL1*	*NPEPL1*
MM115	0	0	0	0	0	0	0	0	0	0	0	0	0	0	0
MM120	0	0	0	0	0	0	0	0	0	0	0	0	0	0	0
MM122	0	0	0	0	0	0	0	0	0	0	0	0	0	0	0
MM124	0	0	0	0	0	0	0	0	0	0	0	0	0	0	0
MM135	0	0	0	0	0	0	0	0	0	0	0	0	0	0	0
MM137	1	0	0	0	1	0	0	0	0	0	0	0	0	0	0
MM139	0	0	0	0	0	0	0	0	0	0	0	0	0	0	0
MM006	0	0	0	0	1	0	0	0	0	0	0	0	0	0	0
MM002_1	0	0	0	0	0	0	0	0	0	0	0	0	0	0	0
MM002_2	0	0	0	0	0	0	0	0	0	0	0	0	0	0	0
MM002_3	0	0	0	0	0	0	0	0	0	0	0	0	0	0	0
MM003	0	0	0	0	0	0	0	0	0	0	0	0	0	0	0
MM004	0	0	0	0	0	0	0	0	0	0	0	0	0	0	0
MM008	0	0	0	0	0	0	0	0	0	0	0	0	0	0	0
MM009	0	0	0	0	0	0	0	0	0	0	0	0	0	0	0
MM011	0	0	0	0	0	0	0	0	0	0	0	0	0	0	0
MM013	0	0	0	0	0	0	0	0	0	0	0	0	0	0	0
MM014_1	0	0	0	0	0	0	0	0	0	0	0	0	0	0	0
MM014_2	0	0	0	0	0	0	0	0	0	0	0	0	0	0	0
MM014_3	0	0	0	0	0	0	0	0	0	0	0	0	0	0	0
MM014_4	0	0	0	0	0	0	0	0	0	0	0	0	0	0	0
MM015	0	0	0	0	0	0	0	0	0	0	0	0	0	0	0
MM016	0	0	0	0	0	0	0	0	0	0	0	0	0	0	0
MM017_1	0	0	0	0	0	0	0	0	0	0	0	0	0	0	0
MM017_3	0	0	0	0	0	0	0	0	0	0	0	0	0	0	0
MM019	0	0	0	0	0	0	0	0	0	0	0	0	0	0	0
MM020	0	0	0	0	0	0	0	0	0	0	0	0	0	0	0
MM022_1	0	0	0	0	0	0	0	0	0	0	0	0	0	0	0
MM022_2	0	0	0	0	0	0	0	0	0	0	0	0	0	0	0
MM022_3	0	0	0	0	0	0	0	0	0	0	0	0	0	0	0
MM023	0	0	0	0	0	0	0	0	0	0	0	0	0	0	0
MM024	0	0	0	0	0	0	0	0	0	0	0	0	0	0	0

Supplementary Table S8. Aminopeptidase gene somatic mutation frequencies from MM patient samples in the FIMM dataset (*n*=169) (3/12)

SampleID	*LVRN*	*BLMH*	*DPP3*	*DPP4*	*DPP7*	*DPP8*	*DPP9*	*ENPEP*	*ERAP1*	*F11*	*LAP3*	*LNPEP*	*METAP2*	*NAALADL1*	*NPEPL1*
MM027	0	0	0	0	0	0	0	0	0	0	0	0	0	0	0
MM028	0	0	0	0	0	0	0	0	0	0	0	0	0	0	0
MM029	0	0	0	0	0	0	0	0	0	0	0	0	0	0	0
MM030_1	0	0	0	0	0	0	0	0	0	0	0	0	0	0	0
MM030_2	0	0	0	0	0	0	0	0	0	0	0	0	0	0	0
MM031_2	0	0	0	0	0	0	0	0	0	0	0	0	0	0	0
MM032_1	0	0	0	0	0	0	0	0	0	0	0	0	0	0	0
MM032_2	0	0	0	0	0	0	0	0	0	0	0	0	0	0	0
MM033_1	0	0	0	0	0	0	0	0	0	0	0	0	0	0	0
MM033_2	0	0	0	0	0	0	0	0	0	0	0	0	0	0	0
MM033_3	0	0	0	0	0	0	0	0	0	0	0	0	0	0	0
MM034_2	0	0	0	0	0	0	0	0	0	0	0	0	0	0	0
MM035	0	0	0	0	0	0	0	0	0	0	0	0	0	0	0
MM036_1	0	0	0	0	0	0	0	0	0	0	0	0	0	0	0
MM036_2	0	0	0	0	0	0	0	0	0	0	0	0	0	0	0
MM038	0	0	0	0	0	0	0	0	0	0	0	0	0	0	0
MM039	0	0	0	0	0	0	0	0	0	0	0	0	0	0	0
MM040	0	0	0	0	0	0	0	0	0	0	0	0	0	0	0
MM043_1	0	0	0	0	0	0	0	0	0	0	0	0	0	0	0
MM044	0	0	0	0	0	0	0	0	0	0	0	0	0	0	0
MM045	0	0	0	0	0	0	0	0	0	0	0	0	0	0	0
MM046_1	0	0	0	0	0	0	0	0	0	0	0	0	0	0	0
MM046_2	0	0	0	0	0	0	0	0	0	0	0	0	0	0	0
MM047	0	0	0	0	0	0	0	0	0	0	0	0	0	0	0
MM049	0	0	0	0	0	0	0	0	0	0	0	0	0	0	0
MM050	0	0	0	0	0	0	0	0	0	0	0	0	0	0	0
MM051_1	0	0	0	0	0	0	0	0	0	0	0	0	0	0	0
MM051_2	0	0	0	0	0	0	0	0	0	0	0	0	0	0	0
MM052_1	0	0	0	0	0	0	0	0	0	0	0	0	0	0	0
MM052_2	0	0	0	0	0	0	0	0	0	0	0	0	0	0	0
MM053	0	0	0	0	0	0	0	0	0	0	0	0	0	0	0
MM054	0	0	0	0	0	0	0	0	0	0	0	0	0	0	0

Supplementary Table S8. Aminopeptidase gene somatic mutation frequencies from MM patient samples in the FIMM dataset (*n*=169) (4/12)

SampleID	*LVRN*	*BLMH*	*DPP3*	*DPP4*	*DPP7*	*DPP8*	*DPP9*	*ENPEP*	*ERAP1*	*F11*	*LAP3*	*LNPEP*	*METAP2*	*NAALADL1*	*NPEPL1*
MM056_1	0	0	0	0	0	0	0	0	0	0	0	0	0	0	0
MM056_2	0	0	0	0	0	0	0	0	0	0	0	0	0	0	0
MM057_1	0	0	0	0	0	0	0	0	0	0	0	0	0	0	0
MM057_2	0	0	0	0	0	0	0	0	0	0	0	0	0	0	0
MM057_3	0	0	0	0	0	0	0	0	0	0	0	0	0	0	0
MM057_4	0	0	0	0	0	0	0	0	0	0	0	0	0	0	0
MM057_5	0	0	0	0	0	0	0	0	0	0	0	0	0	0	0
MM057_6	0	0	0	0	0	0	0	0	0	0	0	0	0	0	0
MM058_1	0	0	0	0	0	0	0	0	0	0	0	0	0	0	0
MM058_2	0	0	0	0	0	0	0	0	0	0	0	0	0	0	0
MM059	0	0	0	0	0	0	0	0	0	0	0	0	0	0	0
MM061_1	0	0	0	0	0	0	0	0	0	0	0	0	0	0	0
MM061_2	0	0	0	0	0	0	0	0	0	0	0	0	0	0	0
MM062	0	0	0	0	0	0	0	0	0	0	0	0	0	0	0
MM066	0	0	0	0	0	0	0	0	0	0	0	0	0	0	0
MM067	0	0	0	0	0	0	0	0	0	0	0	0	0	0	0
MM068_1	0	0	0	0	0	0	0	0	0	0	0	0	0	0	0
MM069	0	0	0	0	0	0	0	0	0	0	0	0	0	0	0
MM070	0	0	0	0	0	0	0	0	0	0	0	0	0	0	0
MM071	0	0	0	0	0	0	0	0	0	0	0	0	0	0	0
MM072	0	0	0	0	0	0	0	0	0	0	0	0	0	0	0
MM073	0	0	0	0	0	0	0	0	0	0	0	0	0	0	0
MM074	0	0	0	0	0	0	0	0	0	0	0	0	0	0	0
MM075	0	0	0	0	0	0	0	0	0	0	0	0	0	0	0
MM076	0	0	0	0	0	0	0	0	0	0	0	0	0	0	0
MM079	0	0	0	0	0	0	0	0	0	0	0	0	0	0	0
MM080	0	0	0	0	0	0	0	0	0	0	0	0	0	0	0
MM081	0	0	0	0	0	0	0	0	0	0	0	0	0	0	0
MM082	0	0	0	0	0	0	0	0	0	0	0	0	0	0	0
MM083_1	0	0	0	0	0	0	0	0	0	0	0	0	0	0	0
MM083_2	0	0	0	0	0	0	0	0	0	0	0	0	0	0	0
MM084	0	0	0	0	0	0	0	0	0	0	0	0	0	0	0

Supplementary Table S8. Aminopeptidase gene somatic mutation frequencies from MM patient samples in the FIMM dataset (*n*=169) (5/12)

SampleID	*LVRN*	*BLMH*	*DPP3*	*DPP4*	*DPP7*	*DPP8*	*DPP9*	*ENPEP*	*ERAP1*	*F11*	*LAP3*	*LNPEP*	*METAP2*	*NAALADL1*	*NPEPL1*
MM085	0	0	0	0	0	0	0	0	0	0	0	0	0	0	0
MM087	0	0	0	0	0	0	0	0	0	0	0	0	0	0	0
MM088	0	0	0	0	0	0	0	0	0	0	0	0	0	0	0
MM089	0	0	0	0	0	0	0	0	0	0	0	0	0	0	0
MM090	0	0	0	0	0	0	0	0	0	0	0	0	0	0	0
MM091	0	0	0	0	0	0	0	0	0	0	0	0	0	0	0
MM092	0	0	0	0	0	0	0	0	0	0	0	0	0	0	0
MM093	0	0	0	0	0	0	0	0	0	0	0	0	0	0	0
MM094	0	0	0	0	0	0	0	0	0	0	0	0	0	0	0
MM095	0	0	0	0	0	0	0	0	0	0	0	0	0	0	0
MM097	0	0	0	0	0	0	0	0	0	0	0	0	0	0	0
MM099	0	0	0	0	0	0	0	0	0	0	0	0	0	0	0
MM100	0	0	0	0	0	0	0	0	0	0	0	0	0	0	0
MM102	0	0	0	0	0	0	0	0	0	0	0	0	0	0	0
MM105	0	0	0	0	0	0	0	0	0	0	0	0	0	0	0
MM108	0	0	0	0	0	0	0	0	0	0	0	0	0	0	0
MM109	0	0	0	0	0	0	0	0	0	0	0	0	0	0	0
MM110	0	0	0	0	0	0	0	0	0	0	0	0	0	0	0
MM111_1	0	0	0	0	0	0	0	0	0	0	0	0	0	0	0
MM111_2	0	0	0	0	0	0	0	0	0	0	0	0	0	0	0
MM113_1	0	0	0	0	0	0	0	0	0	0	0	0	0	0	0
MM113_2	0	0	0	0	0	0	0	0	0	0	0	0	0	0	0
MM114	0	0	0	0	0	0	0	0	0	0	0	0	0	0	0
MM116	0	0	0	0	0	0	0	0	0	0	0	0	0	0	0
MM117_1	0	0	0	0	0	0	0	0	0	0	0	0	0	0	0
MM117_2	0	0	0	0	0	0	0	0	0	0	0	0	0	0	0
MM118	0	0	0	0	0	0	0	0	0	0	0	0	0	0	0
MM119	0	0	0	0	0	0	0	0	0	0	0	0	0	0	0
MM121	0	0	0	0	0	0	0	0	0	0	0	0	0	0	0
MM123	0	0	0	0	0	0	0	0	0	0	0	0	0	0	0
MM126	0	0	0	0	0	0	0	0	0	0	0	0	0	0	0
MM128_1	0	0	0	0	0	0	0	0	0	0	0	0	0	0	0

Supplementary Table S8. Aminopeptidase gene somatic mutation frequencies from MM patient samples in the FIMM dataset (*n*=169) (6/12)

SampleID	*LVRN*	*BLMH*	*DPP3*	*DPP4*	*DPP7*	*DPP8*	*DPP9*	*ENPEP*	*ERAP1*	*F11*	*LAP3*	*LNPEP*	*METAP2*	*NAALADL1*	NPEPL1
MM129	0	0	0	0	0	0	0	0	0	0	0	0	0	0	0
MM130	0	0	0	0	0	0	0	0	0	0	0	0	0	0	0
MM131	0	0	0	0	0	0	0	0	0	0	0	0	0	0	0
MM132	0	0	0	0	0	0	0	0	0	0	0	0	0	0	0
MM133	0	0	0	0	0	0	0	0	0	0	0	0	0	0	0
MM134	0	0	0	0	0	0	0	0	0	0	0	0	0	0	0
MM136	0	0	0	0	0	0	0	0	0	0	0	0	0	0	0
MM138	0	0	0	0	0	0	0	0	0	0	0	0	0	0	0
MM140	0	0	0	0	0	0	0	0	0	0	0	0	0	0	0
**Frequency(%)**	**2,367**	**1,775148**	**0,5917**	**1,1834**	**1,1834**	**0,5917**	**0,5917**	**1,1834**	**1,183432**	**0,59172**	**1,183**	**1,1834**	**0,591716**	**0,59171598**	**1,183431953**

Supplementary Table S8. Aminopeptidase gene somatic mutation frequencies from MM patient samples in the FIMM dataset (*n*=169) (7/12)

SampleID	*NPEPPS*	*RNPEPL1*	*TPP1*	*TPP2*	*TRHDE*	*XPNPEP2*	*XPNPEP3*	Gene symbol
MM026	0	0	0	0	0	0	0	
MM033_4	0	0	0	0	0	0	0	
MM001	0	0	0	0	0	0	0	
MM034_1	0	0	0	0	0	0	0	
MM037	0	0	0	0	0	0	0	
MM041	0	0	0	0	0	0	0	
MM042	0	0	0	0	0	0	0	
MM043_2	0	0	0	0	0	0	0	
MM048_1	0	0	0	0	0	0	0	
MM048_2	0	0	0	0	0	0	0	
MM060	0	0	0	0	0	0	0	
MM063	1	0	0	0	0	0	0	
MM064	0	0	0	0	0	0	0	
MM065	0	0	0	0	0	0	0	
MM068_2	0	0	0	0	0	0	0	
MM077	0	0	0	0	0	0	0	
MM078_1	0	0	0	1	0	0	0	
MM078_2	0	0	0	0	0	0	0	
MM086	0	0	0	0	0	0	0	
MM096	0	0	0	0	0	0	0	
MM005	0	0	0	0	0	0	0	
MM007	0	0	0	0	0	0	0	
MM010	0	0	0	0	0	0	0	
MM017_2	1	0	0	0	0	0	0	
MM025_1	0	0	0	0	0	1	0	
MM025_2	0	0	1	0	0	1	0	
MM055	0	0	0	0	0	0	0	
MM098	0	0	0	0	0	0	0	
MM101	0	0	0	0	0	0	0	
MM103	0	0	0	0	0	0	0	
MM104	0	1	0	0	0	0	0	
MM107	0	0	0	0	0	0	0	

Supplementary Table S8. Aminopeptidase gene somatic mutation frequencies from MM patient samples in the FIMM dataset (*n*=169) (8/12)

SampleID	*NPEPPS*	*RNPEPL1*	*TPP1*	*TPP2*	*TRHDE*	*XPNPEP2*	*XPNPEP3*	Gene symbol
MM115	1	0	0	0	0	0	0	
MM120	1	0	0	0	0	0	0	
MM122	0	0	0	0	0	0	1	
MM124	0	0	0	0	1	0	0	
MM135	0	0	0	0	0	0	0	
MM137	0	1	0	0	0	0	0	
MM139	0	0	0	0	1	0	0	
MM006	0	0	0	0	0	0	0	
MM002_1	0	0	0	0	0	0	0	
MM002_2	0	0	0	0	0	0	0	
MM002_3	0	0	0	0	0	0	0	
MM003	0	0	0	0	0	0	0	
MM004	0	0	0	0	0	0	0	
MM008	0	0	0	0	0	0	0	
MM009	0	0	0	0	0	0	0	
MM011	0	0	0	0	0	0	0	
MM013	0	0	0	0	0	0	0	
MM014_1	0	0	0	0	0	0	0	
MM014_2	0	0	0	0	0	0	0	
MM014_3	0	0	0	0	0	0	0	
MM014_4	0	0	0	0	0	0	0	
MM015	0	0	0	0	0	0	0	
MM016	0	0	0	0	0	0	0	
MM017_1	0	0	0	0	0	0	0	
MM017_3	0	0	0	0	0	0	0	
MM019	0	0	0	0	0	0	0	
MM020	0	0	0	0	0	0	0	
MM022_1	0	0	0	0	0	0	0	
MM022_2	0	0	0	0	0	0	0	
MM022_3	0	0	0	0	0	0	0	
MM023	0	0	0	0	0	0	0	
MM024	0	0	0	0	0	0	0	

Supplementary Table S8. Aminopeptidase gene somatic mutation frequencies from MM patient samples in the FIMM dataset (*n*=169) (9/12)

SampleID	*NPEPPS*	*RNPEPL1*	*TPP1*	*TPP2*	*TRHDE*	*XPNPEP2*	*XPNPEP3*	Gene symbol
MM027	0	0	0	0	0	0	0	
MM028	0	0	0	0	0	0	0	
MM029	0	0	0	0	0	0	0	
MM030_1	0	0	0	0	0	0	0	
MM030_2	0	0	0	0	0	0	0	
MM031_2	0	0	0	0	0	0	0	
MM032_1	0	0	0	0	0	0	0	
MM032_2	0	0	0	0	0	0	0	
MM033_1	0	0	0	0	0	0	0	
MM033_2	0	0	0	0	0	0	0	
MM033_3	0	0	0	0	0	0	0	
MM034_2	0	0	0	0	0	0	0	
MM035	0	0	0	0	0	0	0	
MM036_1	0	0	0	0	0	0	0	
MM036_2	0	0	0	0	0	0	0	
MM038	0	0	0	0	0	0	0	
MM039	0	0	0	0	0	0	0	
MM040	0	0	0	0	0	0	0	
MM043_1	0	0	0	0	0	0	0	
MM044	0	0	0	0	0	0	0	
MM045	0	0	0	0	0	0	0	
MM046_1	0	0	0	0	0	0	0	
MM046_2	0	0	0	0	0	0	0	
MM047	0	0	0	0	0	0	0	
MM049	0	0	0	0	0	0	0	
MM050	0	0	0	0	0	0	0	
MM051_1	0	0	0	0	0	0	0	
MM051_2	0	0	0	0	0	0	0	
MM052_1	0	0	0	0	0	0	0	
MM052_2	0	0	0	0	0	0	0	
MM053	0	0	0	0	0	0	0	
MM054	0	0	0	0	0	0	0	

Supplementary Table S8. Aminopeptidase gene somatic mutation frequencies from MM patient samples in the FIMM dataset (*n*=169) (10/12)

SampleID	*NPEPPS*	*RNPEPL1*	*TPP1*	*TPP2*	*TRHDE*	*XPNPEP2*	*XPNPEP3*	Gene symbol
MM056_1	0	0	0	0	0	0	0	
MM056_2	0	0	0	0	0	0	0	
MM057_1	0	0	0	0	0	0	0	
MM057_2	0	0	0	0	0	0	0	
MM057_3	0	0	0	0	0	0	0	
MM057_4	0	0	0	0	0	0	0	
MM057_5	0	0	0	0	0	0	0	
MM057_6	0	0	0	0	0	0	0	
MM058_1	0	0	0	0	0	0	0	
MM058_2	0	0	0	0	0	0	0	
MM059	0	0	0	0	0	0	0	
MM061_1	0	0	0	0	0	0	0	
MM061_2	0	0	0	0	0	0	0	
MM062	0	0	0	0	0	0	0	
MM066	0	0	0	0	0	0	0	
MM067	0	0	0	0	0	0	0	
MM068_1	0	0	0	0	0	0	0	
MM069	0	0	0	0	0	0	0	
MM070	0	0	0	0	0	0	0	
MM071	0	0	0	0	0	0	0	
MM072	0	0	0	0	0	0	0	
MM073	0	0	0	0	0	0	0	
MM074	0	0	0	0	0	0	0	
MM075	0	0	0	0	0	0	0	
MM076	0	0	0	0	0	0	0	
MM079	0	0	0	0	0	0	0	
MM080	0	0	0	0	0	0	0	
MM081	0	0	0	0	0	0	0	
MM082	0	0	0	0	0	0	0	
MM083_1	0	0	0	0	0	0	0	
MM083_2	0	0	0	0	0	0	0	
MM084	0	0	0	0	0	0	0	

Supplementary Table S8. Aminopeptidase gene somatic mutation frequencies from MM patient samples in the FIMM dataset (*n*=169) (11/12)

SampleID	*NPEPPS*	*RNPEPL1*	*TPP1*	*TPP2*	*TRHDE*	*XPNPEP2*	*XPNPEP3*	Gene symbol
MM085	0	0	0	0	0	0	0	
MM087	0	0	0	0	0	0	0	
MM088	0	0	0	0	0	0	0	
MM089	0	0	0	0	0	0	0	
MM090	0	0	0	0	0	0	0	
MM091	0	0	0	0	0	0	0	
MM092	0	0	0	0	0	0	0	
MM093	0	0	0	0	0	0	0	
MM094	0	0	0	0	0	0	0	
MM095	0	0	0	0	0	0	0	
MM097	0	0	0	0	0	0	0	
MM099	0	0	0	0	0	0	0	
MM100	0	0	0	0	0	0	0	
MM102	0	0	0	0	0	0	0	
MM105	0	0	0	0	0	0	0	
MM108	0	0	0	0	0	0	0	
MM109	0	0	0	0	0	0	0	
MM110	0	0	0	0	0	0	0	
MM111_1	0	0	0	0	0	0	0	
MM111_2	0	0	0	0	0	0	0	
MM113_1	0	0	0	0	0	0	0	
MM113_2	0	0	0	0	0	0	0	
MM114	0	0	0	0	0	0	0	
MM116	0	0	0	0	0	0	0	
MM117_1	0	0	0	0	0	0	0	
MM117_2	0	0	0	0	0	0	0	
MM118	0	0	0	0	0	0	0	
MM119	0	0	0	0	0	0	0	
MM121	0	0	0	0	0	0	0	
MM123	0	0	0	0	0	0	0	
MM126	0	0	0	0	0	0	0	
MM128_1	0	0	0	0	0	0	0	

Supplementary Table S8. Aminopeptidase gene somatic mutation frequencies from MM patient samples in the FIMM dataset (*n*=169) (12/12)

SampleID	*NPEPPS*	*RNPEPL1*	*TPP1*	*TPP2*	*TRHDE*	*XPNPEP2*	*XPNPEP3*	Gene symbol
MM129	0	0	0	0	0	0	0	
MM130	0	0	0	0	0	0	0	
MM131	0	0	0	0	0	0	0	
MM132	0	0	0	0	0	0	0	
MM133	0	0	0	0	0	0	0	
MM134	0	0	0	0	0	0	0	
MM136	0	0	0	0	0	0	0	
MM138	0	0	0	0	0	0	0	
MM140	0	0	0	0	0	0	0	
**Frequency(%)**	**2,3668639**	**1,183432**	**0,59171598**	**0,5917**	**1,18343**	**1,183432**	**0,59171598**	

Supplementary Table S9. Aminopeptidase gene copy number variation scores from MM patient samples in the FIMM dataset (*n* = 169). (1/15)

SampleID	*AMZ1*	*ANPEP*	*LVRN*	*BLMH*	*AOPEP*	*CTSH*	*CTSV*	*DNPEP*	*DPP3*	*DPP4*	*DPP7*	*DPP8*	*DPP9*	*ENPEP*	*ERAP1*	*ERAP2*	Gene
MM032_1	-0.0975	0.307	0.0338	0.0332	0.2042	0.307	0.2042	-0.1779	0.2072	-0.1779	0.2042	0.307	0.4492	-0.3369	0.0338	0.0338	
MM051_1	-0.755	-1.1296	-0.0794	0.067	-0.0189	0.0117	-0.0189	-0.0119	-0.0184	-0.07	-0.255	0.0216	-0.0331	-0.1449	-0.0794	-0.0794	
MM053	-0.0514	-1.2361	0.5862	-0.1723	0.4213	0.3532	0.5355	-0.3912	0.354	0.032	0.0024	0.3688	0.0805	0.1448	0.5862	0.5862	
MM056_1	0.1455	0.5006	0.0042	0.1582	0.1646	0.5006	0.1646	-0.3571	0.1891	0.1666	0.1099	0.5006	0.4936	-0.3229	0.0042	0.0042	
MM062	0.0786	0.0339	-0.5005	0.0519	-0.0674	-0.17955	-0.0674	-0.1844	0.038	-0.1857	0.0475	-0.0759	0.0705	-0.3681	-0.4436	-0.4436	
MM063	0.0081	0.012	-0.0198	0.0444	0.0223	0.012	0.0223	-0.0184	0.0434	-0.0184	0.0223	-0.0014	0.0857	-0.0444	-0.0198	-0.0198	
MM064	0.3701	0.459	0.232	-0.0471	0.5218	0.459	0.5218	-0.1483	-0.0781	-0.1483	0.5218	0.4296	0.7849	-1.0909	0.232	0.232	
MM065	-0.0101	0.1105	-0.0654	0.0313	0.0778	0.1105	0.0778	-0.0343	0.0091	-0.0343	0.0778	0.1105	0.7353	-0.7433	-0.0654	-0.0654	
MM066	-0.027	-0.0156	-0.065	0.0968	0.0211	-0.0156	0.0211	-0.0711	0.0492	-0.0711	0.0211	-0.0156	0.2075	-0.1092	-0.065	-0.065	
MM067	0.2429	0.2568	-0.0112	0.3744	0.7107	0.2568	0.7107	-0.2913	0.3213	-0.4561	0.7107	0.2568	0.6302	-0.5339	-0.0112	-0.0112	
MM069	0.1898	0.4124	0.1396	-0.097	0.2028	0.4124	0.2028	-0.1456	0.2025	-0.1456	0.2028	0.4124	0.3396	-0.1699	0.1396	0.1396	
MM070	0.2034	0.2227	0.1852	0.2508	0.2342	0.2227	0.2342	-0.1521	0.224	-0.1521	0.2342	0.2227	0.2909	-0.1679	0.1852	0.1852	
MM072	-0.0112	0.2957	0.2322	0.3667	0.321	0.2957	0.321	-0.2604	0.3	-0.2768	0.321	0.2957	0.4684	-0.3076	0.2322	0.2322	
MM074	0.0033	0.0057	-0.0659	-0.4887	0.0165	0.0057	0.0165	-0.0326	-0.0049	-0.0326	0.0165	0.0057	0.1794	-0.0297	-0.0659	-0.0659	
MM075	-0.8238	0.373	0.3051	-0.0427	0.3809	0.373	0.3809	-0.1144	0.6632	-0.1144	0.3809	0.373	0.3941	-0.1393	0.3051	0.3051	
MM076	-0.035	-0.0533	-0.072	-0.0037	0.1944	-0.0533	0.1944	-0.0699	0.209	-0.0699	0.1944	-0.0533	0.3156	-0.1045	-0.072	-0.072	
MM078_1	-0.0268	-0.0085	-0.1099	0.0912	0.0044	-0.0085	0.0044	-0.0801	0.0186	-0.0801	0.0044	-0.0085	0.5883	-0.1369	-0.1099	-0.1099	
MM079	-0.0483	-0.0466	0.3454	0.0487	0.7421	-0.0466	0.7421	-0.0936	0.4107	-0.0936	0.7421	-0.0466	0.634	-0.1274	0.3454	0.3454	
MM083_1	0.001	0.0073	-0.0235	0.0845	0.0166	0.0073	0.0166	-0.0277	0.0138	-0.0277	0.0166	0.0073	0.1676	-0.052	-0.0235	-0.0235	
MM085	-0.0682	0.2245	0.2763	-0.0839	0.2159	0.2245	0.2159	-0.0645	0.2093	-0.0645	0.1709	0.2245	0.4283	-0.0228	0.2631	0.2631	
MM086	0.0159	0.0835	-0.0333	0.1559	0.0536	0.0835	0.0536	0.0291	0.2094	-0.1119	0.0536	0.0147	0.9246	-0.0988	-0.0333	-0.0333	
MM087	-0.0191	-0.0248	-0.078	0.0883	0.0175	-0.0248	0.0175	-0.0267	0.1553	-0.1024	0.0175	-0.0248	0.6223	-0.14	-0.078	-0.078	
MM088	0.0193	0.0123	-0.011	0.0877	0.0458	0.0123	0.0458	0.0083	0.8516	-0.0489	0.0458	0.0123	0.2294	-0.0588	-0.011	-0.011	
MM089	0.2046	-0.0081	-0.1051	0.0811	0.0377	-0.0081	0.0377	-0.0987	0.1115	-0.0987	0.0377	-0.0081	0.2776	-0.1188	-0.1051	-0.1051	
MM090	-0.0173	-0.0065	-0.1677	0.118	0.0626	-0.0065	0.0626	-0.0743	0.1405	-0.0743	0.0626	-0.0065	0.2254	-0.1492	-0.1422	-0.1422	
MM093	0.2536	0.2789	0.2178	0.0372	0.2841	0.2714	0.2841	-0.2267	-0.0358	-0.2267	0.2841	0.2714	0.47	-0.205	0.2178	0.2178	
MM094	-0.0705	0.7592	0.368	0.0048	0.4254	0.7592	0.4254	-0.1292	0.4288	-0.1292	0.4254	0.7592	0.591	-0.1741	0.368	0.368	
MM096	0.0025	-0.0064	-0.0289	0.0447	-0.0011	-0.0064	-0.0011	-0.0115	-0.0109	-0.0497	-0.0011	-0.0064	0.121	-0.0339	-0.0289	-0.0289	
MM100	0.0156	-0.0033	-0.0316	0.0957	0.4449	-0.0033	0.4449	-0.051	0.0097	-0.051	0.4449	0.0097	0.2649	-0.0823	-0.0316	-0.0316	
MM101	0.261	0.6357	0.2479	-0.1768	0.2929	0.6357	0.2929	-0.2698	0.2984	-0.2698	0.2929	0.6357	0.4647	-0.2659	0.2396	0.2396	
MM103	-0.106	0.2846	0.2489	-0.0016	0.3262	0.2846	0.3262	-0.212	0.3373	-0.212	0.3262	0.2846	0.5754	-0.2107	0.2489	0.2489	
MM104	0.0118	-0.0115	-0.1447	0.1188	-0.0739	-0.0115	-0.0739	-0.0835	0.1566	-0.1654	0.0673	-0.0115	0.7673	-0.1403	-0.1447	-0.1447	
MM105	-0.0493	0.3065	-0.0816	0.0311	0.3038	0.3065	0.3038	-0.0861	0.3034	-0.1133	0.3038	0.3065	0.4943	-0.102	-0.0816	-0.0816	
MM108	-0.0155	-0.0118	-0.2104	0.0813	-0.0509	-0.0118	-0.0509	-0.9191	0.1614	-1.0938	0.5943	-0.0118	0.3042	-0.1598	-0.2104	-0.2104	
MM110	0.024	0.0094	-0.0318	0.0866	0.0292	0.0094	0.0292	-0.0084	0.0193	-0.0825	0.0292	0.0094	0.2085	-0.061	-0.0318	-0.0318	

Supplementary Table S9. Aminopeptidase gene copy number variation scores from MM patient samples in the FIMM dataset (*n* = 169). (2/15)

SampleID	*AMZ1*	*ANPEP*	*LVRN*	*BLMH*	*AOPEP*	*CTSH*	*CTSV*	*DNPEP*	*DPP3*	*DPP4*	*DPP7*	*DPP8*	*DPP9*	*ENPEP*	*ERAP1*	*ERAP2*	Gene
MM113_1	0.0981	0.7424	0.2975	0.0033	0.3898	0.7424	0.3898	-0.1844	0.719	-0.1844	0.3898	0.7424	0.6314	-0.223	0.2975	0.2975	
MM114	0	0	0	0	0	0	0	0	0	0	0	0	0	0	0	0	
MM115	0	0	0.1164	0	0	0	0	0.1257	0	0.1257	0	0	0	0.195	0.1164	0.1164	
MM116	-0.0334	0.089	0.0282	0.1073	0.1045	0.089	0.1045	-0.0735	0.0074	-0.1381	0.1045	0.0816	0.3998	-0.1194	0.0282	0.0282	
MM117_1	0	0	0	0	0	0	0	0	0	0	0	0	0	0	0	0	
MM118	0.2243	0.0411	-0.0863	0.0685	0.0122	0.0411	0.0122	-0.0267	0.0394	-0.131	0.0122	0.026	0.8383	-0.1058	-0.0863	-0.0863	
MM119	0.3004	0.0089	0.081	0.0883	0.5814	0.0089	0.5814	-0.3463	-0.3445	-0.3246	0.5814	0.0563	0.1032	0.0715	0.081	0.081	
MM120	0.2538	0.0685	0.1844	-0.0332	0.0971	0.0685	0.0971	-0.0789	0.1897	-0.0789	0.2216	0.0685	0.1242	-0.086	0.1844	0.1844	
MM121	0.0039	0.0073	-0.026	0.095	0.0288	0.0073	0.0288	-0.0447	0.0147	-0.0447	0.0288	0.0073	0.2553	-0.0487	-0.026	-0.026	
MM122	-0.0071	0.0021	-0.0366	0.0493	-0.0031	0.0021	-0.0031	-0.0241	0.0126	-0.0241	0.0408	0.0021	0.1424	-0.0283	-0.0366	-0.0366	
MM123	-0.001	0.0189	-0.0487	0.1473	0.0429	0.0132	0.0429	-0.0608	0.0314	-0.0608	0.0429	0.0132	0.3272	-0.0742	-0.0487	-0.0487	
MM126	-0.0034	-0.0086	-0.0234	0.0231	0.1372	-0.0086	0.1372	-0.0251	0.0477	-0.0251	0.1372	-0.0086	0.0628	-0.0258	-0.0234	-0.0234	
MM128_1	0.0326	0.0372	-0.0272	0.1801	-1.0037	0.0372	-1.0037	-0.1478	0.0602	-0.1478	0.1604	0.0372	0.3163	-0.1022	-0.0272	-0.0272	
MM131	0.296	0.0064	-0.19	0.1746	-0.0721	0.0064	-0.0721	-0.1139	0.0161	-0.1139	0.2065	0.0064	0.366	-0.1421	-0.19	-0.19	
MM132	0.0713	0.4834	-0.1447	0.1227	0.0002	0.4834	0.0002	-0.1767	0.4766	-0.1767	0.0002	0.4834	0.9032	-0.2075	-0.1447	-0.1447	
MM133	0.1662	0.2365	-0.2809	0.1107	-0.0397	0.0628	0.07895	-0.0967	0.2439	-0.4032	0.1839	0.0628	0.526	-0.2891	-0.2809	-0.2809	
MM134	0.0411	-0.005	-0.184	0.0265	0.096	-0.005	0.096	-0.3238	0.067	-0.3238	0.096	-0.0001	0.2631	0.1852	-0.184	-0.184	
MM136	0.2435	0.0118	-0.1013	0.1611	-0.0094	0.0118	-0.0094	-0.0698	0.0175	-0.1124	0.1393	0.0118	0.3802	-0.1356	-0.1013	-0.1013	
MM138	0.3916	0.2632	-0.2605	0.2687	0.0012	0.0518	0.0012	-0.02	0.284	-0.3427	0.1949	0.0518	0.5482	-0.2413	-0.2605	-0.2605	
MM139	0.4317	0.6791	-0.3402	0.3159	-0.0336	0.4896	-0.0336	0.0017	0.304	-0.4317	0.2844	0.4896	0.597	-0.2695	-0.9595	-0.9595	
MM140	-0.1009	-0.0828	-0.1433	0.0374	0.2739	-0.0828	0.2739	-0.0967	0.3173	-0.1961	0.3621	-0.0828	0.1595	0.1905	-0.1433	-0.1433	
MM013	0.0048	-0.0132	-0.028	0.0542	0.0013	-0.0132	0.0013	-0.041	0.0005	-0.041	0.1418	-0.018	0.6527	-0.0419	-0.028	-0.028	
MM014_3	0.587	0.3512	-0.3183	0.4584	0.2001	0.08665	0.2001	-0.3347	-0.067	-0.6235	0.5491	0.1511	0.81	-0.0791	-0.5559	-0.5559	
MM016	0.2991	-0.0105	-0.1014	0.0333	0.3452	-0.0105	0.3452	-0.1162	-0.0202	-0.146	0.3452	-0.0816	0.473	-0.1775	-0.1014	-0.1014	
MM017_3	0.2179	0.4582	-0.2302	0.0239	0.3405	0.4582	0.3405	-0.1749	-0.0726	-0.1749	0.5417	0.4582	0.5601	-0.4785	-0.2302	-0.2302	
MM020	0.0703	0.4541	-0.1035	-0.0672	0.2764	0.4541	0.2764	0.051	0.1189	0.051	0.3367	0.4049	0.1377	0.031	-0.1035	-0.1035	
MM026	-0.1683	0.3882	0.3503	0.4443	0.7514	0.3882	0.7514	-0.2091	0.4133	-0.2091	0.8412	0.3882	-0.0001	0.3137	0.3503	0.3503	
MM027	0.006	-0.0029	-0.0607	0.2363	0.0909	-0.0029	0.0909	-0.5181	0.0627	-0.5181	0.6311	-0.0342	0.2408	-0.1225	-0.0607	-0.0607	
MM028	0.0001	0.0099	-0.0364	0.0743	0.0127	0.0099	0.0127	-0.086	0.0391	-0.086	0.0127	0.0099	0.1997	-0.1491	-0.0364	-0.0364	
MM029	-0.0055	-0.0142	-0.0524	0.0874	-0.0094	-0.0142	-0.0094	-0.0412	0.0019	-0.117	-0.0094	-0.0142	0.2235	-0.0874	-0.0524	-0.0524	
MM030_1	-0.1878	0.4714	0.0967	0.1973	0.0742	-0.1721	0.0742	-0.2386	0.1878	0.2209	0.4384	-0.1721	0.4361	-0.2722	0.0967	0.0967	
MM030_2	-0.1107	0.1455	0.0176	0.1238	0.1091	0.1455	0.1091	-0.1673	0.1082	0.1023	0.182	0.1455	0.2979	-0.1998	0.0176	0.0176	
MM031_2	0.1396	0.0509	0.0921	-0.0348	0.0736	0.0509	0.0153	0.0759	0.5985	0.1736	0.1755	-0.0635	0.1122	-0.0598	0.0287	0.0287	
MM032_2	0.0435	0.2846	0.2626	-0.1495	0.1063	0.2846	0.1063	-0.1231	0.0512	0.089	-0.0652	0.2846	-0.0264	0.2611	0.2626	0.2626	
MM033_4	-0.0462	0.4365	-0.8908	-0.0428	0.0174	0.4365	0.0174	-0.1052	-0.0078	-0.1052	0.0174	0.4365	0.2827	-0.0593	-0.8908	-0.8908	

Supplementary Table S9. Aminopeptidase gene copy number variation scores from MM patient samples in the FIMM dataset (*n* = 169). (3/15)

SampleID	*AMZ1*	*ANPEP*	*LVRN*	*BLMH*	*AOPEP*	*CTSH*	*CTSV*	*DNPEP*	*DPP3*	*DPP4*	*DPP7*	*DPP8*	*DPP9*	*ENPEP*	*ERAP1*	*ERAP2*	Gene
MM033_1	-0.123	0.5825	-0.1065	-0.0424	-0.0472	0.4368	-0.0472	-0.0685	-0.0508	-0.0542	-0.1781	0.4588	0.3495	-0.0595	-0.1065	-0.1065	
MM033_2	-0.0106	0.3312	-0.6202	-0.0602	0.0145	0.3312	0.0145	-0.0992	-0.0277	0.0352	-0.098	0.4994	0.3055	0.1236	-0.6202	-0.6202	
MM033_3	-0.0338	0.5279	-0.87	-0.0246	0.0255	0.5279	0.0255	-0.0307	-0.021	-0.0307	-0.0769	0.5279	0.304	0.0063	-0.87	-0.87	
MM001	-0.0217	0.0484	0.0416	-0.6242	0.0363	0.0484	0.0363	0.0344	0.0348	0.0344	0.0363	0.0652	0.0058	-0.0443	0.0416	0.0416	
MM034_2	0.0355	0.0255	-0.0102	0.1108	0.0335	0.0255	0.0335	-0.0578	0.0221	-0.0578	0.0335	0.0255	0.3345	-0.0739	-0.0102	-0.0102	
MM034_1	0.061	0.2868	-0.128	-0.0828	0.0655	0.2868	0.0655	-0.1034	0.0949	-0.3068	0.3062	0.1091	0.5217	-0.38	-0.128	-0.128	
MM035	0.0285	0.0606	-0.1661	0.1313	0.0587	0.0186	0.0587	-0.7978	0.1551	-0.1656	0.0587	0.0186	0.2826	-0.1278	-0.2842	-0.2842	
MM036_1	-0.0719	-0.1136	0.4098	-0.0458	0.4739	-0.1136	0.4739	-0.1934	-0.0925	-0.1934	0.4739	-0.1136	0.7862	-0.2247	0.4098	0.4098	
MM036_2	0.0055	-0.1228	0.3928	-0.0518	0.4323	-0.1228	0.4323	-0.1877	-0.0878	-0.1877	0.5286	-0.1228	0.7461	-0.1171	0.3928	0.3928	
MM037	-0.046	-0.6352	-0.0706	0.1053	0.5103	-0.6352	0.5103	-0.0637	0.0318	-0.0637	0.5103	0.0216	0.2159	-0.0893	-0.0706	-0.0706	
MM038	0.6036	-0.0447	0.1001	0.0569	0.3096	-0.0447	0.3096	-0.1153	0.1958	-0.3864	0.7198	-0.0447	0.7074	-0.2212	0.1001	0.1001	
MM039	-0.1134	0.4981	-0.1896	0.1066	0.3798	0.4981	0.3798	-0.1734	0.5603	-0.1734	0.5703	0.4981	0.7335	-0.2097	-0.1896	-0.1896	
MM040	0.1198	-0.0015	-0.0369	0.0802	0.0116	-0.0015	0.0116	-0.0308	0.0058	-0.0308	0.0116	-0.0015	0.116	-0.0688	-0.0369	-0.0369	
MM041	-0.0064	0.0137	-0.2723	-0.0139	0.0032	0.0137	0.0032	-0.0462	0.0561	-0.1536	0.0909	0.0029	0.2388	-0.1506	-0.2723	-0.2723	
MM042	0.00665	0.3617	0.3162	0.4542	0.2812	0.3617	0.2812	-0.1437	-0.0541	-0.1437	0.1048	0.1767	0.3924	-0.1784	0.3162	0.3162	
MM043_1	-0.0154	-0.0027	-0.0082	-0.0512	-0.003	-0.0027	-0.003	-0.017	-0.0012	-0.0174	-0.003	-0.0027	0.1041	-0.0175	-0.0082	-0.0082	
MM043_2	0.1766	0.0522	0.2132	0.1631	0.1257	0.0522	0.1257	0.2436	-0.0559	0.295	-0.1284	0.1239	-0.0564	-0.002	0.4691	0.4691	
MM044	-0.0001	0.079	0.005	0.0305	0.0294	0.079	0.0294	-0.0259	0.0132	-0.0259	0.0937	0.079	0.3262	-0.0436	0.005	0.005	
MM045	0.2035	0.3384	0.0194	0.0733	0.1486	0.3384	0.1486	0.0114	0.2401	-0.0168	0.1486	0.3384	0.2831	-0.0367	0.0194	0.0194	
MM046_1	0.0343	0.0072	0.0223	-0.0231	0.0177	0.0072	0.0177	0.0302	-0.0032	0.0302	-0.0416	0.0072	-0.0219	0.0505	0.0223	0.0223	
MM046_2	0.0343	0.0072	0.0223	-0.0231	0.0177	0.0072	0.0177	0.0302	-0.0032	0.0302	-0.0416	0.0072	-0.0219	0.0505	0.0223	0.0223	
MM047	0.2159	0.409	0.3283	0.3415	0.4747	0.409	0.4747	-0.155	0.7073	-0.155	0.4747	0.3729	0.562	-0.1992	0.3283	0.3283	
MM048_1	-0.1054	0.3767	0.2144	-0.0583	0.4	0.3767	0.4	-0.129	0.3709	-0.129	0.4	0.3767	0.4753	-0.1335	0.2144	0.2144	
MM048_2	-0.0972	0.4303	0.1494	-0.0993	0.5008	0.3866	0.5008	-0.0892	0.4026	0.1149	0.5324	0.4102	0.471	-0.0738	0.4845	0.4845	
MM049	0.5797	0.7937	0.262	-0.0065	0.4342	0.7937	0.4342	-0.2552	0.8338	-0.2858	0.4342	0.7859	0.7398	-0.2958	0.262	0.262	
MM050	-0.6249	0.0393	0.0091	0.0638	0.0481	0.0393	0.0481	-0.7303	0.0442	-0.7303	0.0481	0.0119	0.0966	-0.0059	0.0091	0.0091	
MM051_2	0.0548	0.0481	0.0701	0.1284	0.1139	-0.0467	0.055	-0.0637	0.0022	0.1258	0.1099	0.0694	0.1663	0.0119	0.0387	0.0387	
MM052_1	-0.0551	-0.0188	-0.0898	0.0587	-0.0383	-0.0188	-0.0383	-0.0594	0.1243	-0.1132	-0.0111	-0.0188	0.1574	-0.1046	-0.0898	-0.0898	
MM052_2	-2.5977	-0.1548	0.023	-0.1583	-0.0953	-0.1548	-0.0953	-0.217	-0.5225	0.0422	-1.3281	-0.1192	-0.6232	0.0404	0.023	0.023	
MM054	-0.7092	0.2638	-0.0781	0.1634	-0.0639	0.2638	-0.0639	-0.0144	0.199	-0.1767	0.2446	0.2638	0.5405	-0.141	-0.0781	-0.0781	
MM055	0.4468	0.3237	0.204	-0.5385	0.2929	0.3237	0.2929	-0.1592	0.3079	-0.1592	0.3789	0.3054	0.6352	-0.2178	0.204	0.204	
MM056_2	0.237	0.6464	0.2105	-0.1695	0.259	0.6464	0.259	0.0069	0.2867	0.0069	0.1337	0.6464	0.1404	-0.2136	0.2105	0.2105	
MM057_4	-0.0988	0.3765	0.0727	0.1959	0.4185	0.3765	0.4185	-0.1372	0.4527	-0.1372	0.4185	0.3765	0.0744	-0.1745	0.0727	0.0727	
MM057_5	0.0036	0.0119	-0.0345	0.0594	0.0081	0.0119	0.0081	-0.0396	0.5647	-0.0396	0.0081	-0.0793	0.1709	-0.0811	-0.0345	-0.0345	
MM057_6	-0.0224	-0.0642	-0.0516	0.0485	-0.0911	-0.0642	-0.0911	-0.0568	0.5786	-0.0568	0.0542	-0.0642	0.1673	-0.088	-0.0516	-0.0516	

Supplementary Table S9. Aminopeptidase gene copy number variation scores from MM patient samples in the FIMM dataset (*n* = 169). (4/15)

SampleID	*AMZ1*	*ANPEP*	*LVRN*	*BLMH*	*AOPEP*	*CTSH*	*CTSV*	*DNPEP*	*DPP3*	*DPP4*	*DPP7*	*DPP8*	*DPP9*	*ENPEP*	*ERAP1*	*ERAP2*	Gene
MM057_1	-0.0375	0.0112	-0.014	0.0313	0.022	0.0112	0.022	-0.0717	0.5189	-0.0717	0.022	0.0112	0.1452	-0.1145	-0.014	-0.014	
MM057_2	-0.0263	-0.0113	-0.0569	0.0808	0.0343	-0.0113	0.0343	-0.0825	0.0728	-0.0825	0.0343	-0.0113	0.1843	-0.1156	-0.0569	-0.0569	
MM057_3	-0.0982	0.3885	0.3525	-0.0114	0.4612	0.3885	0.4612	-0.1648	0.5219	-0.1648	0.4612	0.3885	0.3634	-0.2286	0.3525	0.3525	
MM058_1	0.154	0.0781	-0.1123	0.1417	0.1054	0.0781	0.1054	0.0464	0.0919	0.0464	0.1054	0.0781	0.2239	-0.0578	-0.1123	-0.1123	
MM058_2	1.647	0.3232	-0.306	0.6695	-0.1448	0.3232	-0.1448	-0.3445	0.9038	-0.3445	1.7234	-0.0781	2.1462	-0.9946	-0.306	-0.306	
MM059	-0.0267	-0.0169	-0.0529	0.1338	0.0511	-0.0169	0.0511	-0.0618	0.134	-0.0618	0.0511	-0.0169	0.2991	-0.1404	-0.0529	-0.0529	
MM060	0.2691	-0.0175	-0.1357	0.1318	-0.097	-0.0175	-0.097	-0.2249	0.0789	-0.1772	0.4673	-0.0175	0.8746	-0.0089	-0.1357	-0.1357	
MM061_1	-0.0125	0.0008	-0.0279	0.1149	-0.0124	0.0008	-0.0124	-0.0353	-0.0096	-0.0353	-0.0124	0.0008	0.5168	-0.0371	-0.0279	-0.0279	
MM061_2	-0.0278	-0.0008	-0.0331	0.0611	-0.0034	-0.0008	-0.0034	-0.0222	-0.018	-0.0307	-0.0034	-0.0008	0.5917	-0.022	-0.0331	-0.0331	
MM068_1	0.0054	-0.6066	-0.0157	0.0777	0.0314	0.0184	0.0314	-0.0195	0.2311	-0.0195	0.0314	0.0184	0.1328	-0.0449	-0.0157	-0.0157	
MM068_2	-0.1714	-0.3069	-0.2202	0.3978	0.2082	-0.3069	0.2082	-0.3739	0.9251	-0.3739	0.2082	-0.3069	0.9125	-0.748	-0.2202	-0.2202	
MM071	0.6165	0.6	-0.2244	-0.0288	0.3244	0.6	0.3244	-0.319	0.5397	-0.319	-0.0545	0.6	0.7728	-0.3458	-0.2244	-0.2244	
MM073	0.4564	0.4311	0.2984	-0.0769	0.7192	0.4311	0.7192	-0.1004	0.4375	-0.1961	0.7192	0.4311	0.4495	-0.1392	0.2984	0.2984	
MM077	-0.0041	-0.0446	-0.2773	0.0525	0.5312	-0.0446	0.5312	-0.2574	-0.0001	-0.2574	0.5312	-0.0446	0.0943	0.1157	-0.1914	-0.1914	
MM078_2	0.0104	-0.001	-0.0397	0.1041	0.0182	-0.001	0.0182	-0.0088	0.0073	-0.0826	0.0182	-0.001	0.7387	-0.0819	-0.0397	-0.0397	
MM080	0.0598	-0.7869	-0.0654	0.073	0.4599	-0.7869	0.4599	-0.0957	0.0121	-0.0957	0.5644	0.0348	0.204	-0.1019	-0.0654	-0.0654	
MM081	0.4436	0.09	-0.1872	0.2911	-0.03	0.1025	-0.03	0.0993	0.2884	-0.3186	0.2265	0.1025	0.6829	-0.2404	-0.1872	-0.1872	
MM082	0.5554	0.8231	0.3097	-0.0287	0.4523	0.8231	0.4523	-0.2364	0.4558	-0.2364	0.4523	0.8208	0.6772	-0.2629	0.3097	0.3097	
MM083_2	-0.0183	0.0205	-0.0274	0.0704	0.0287	0.0205	0.0287	-0.0099	0.0243	-0.06	0.0287	0.0205	0.1651	-0.0461	-0.0274	-0.0274	
MM084	0.371	0.3773	0.3621	-0.1303	0.3821	0.3773	0.3821	-0.172	0.374	-0.172	0.3821	0.3773	0.4714	-0.1637	0.3621	0.3621	
MM091	-0.0872	0.3971	0.2523	0.0161	0.4196	0.3971	0.4196	-0.1797	0.3977	-0.1797	0.4196	0.3971	1.0537	-0.2703	0.2523	0.2523	
MM092	-0.0116	-0.0037	-0.1218	0.0474	0.0027	-0.0037	0.0027	-0.0364	0.0018	-0.0364	0.0027	-0.0037	0.1237	-0.0648	-0.1218	-0.1218	
MM095	0.0085	-0.022	-0.1058	0.0608	-0.0408	-0.022	-0.0408	-0.0984	0.026	-0.1312	0.0986	-0.022	0.2101	-0.1701	-0.1058	-0.1058	
MM097	-0.0008	0.0881	-0.1391	0.1246	0.0058	0.0881	0.0058	-0.1512	0.2706	-0.1512	0.0553	0.1513	0.397	-0.1127	-0.1391	-0.1391	
MM098	0.0517	-0.518	0.06	-0.121	0.0055	-0.518	0.0055	0.1179	0.0083	0.1179	0.0055	-0.518	-0.7449	0.2239	0.1084	0.1084	
MM099	-0.0342	0.2767	-0.1354	0.1549	-0.1356	0.3118	-0.1356	0.0199	0.0756	-1.0329	0.4046	0.239	0.3856	-0.2773	-0.1354	-0.1354	
MM102	-0.0267	0.0001	0.0038	0.1086	0.0659	0.0001	0.0659	-0.0739	0.0005	-0.0739	0.0659	0.0001	0.2785	-0.0949	0.0038	0.0038	
MM107	0.7082	0.3836	-0.1428	-0.7017	0.3189	0.3836	0.3189	-0.1093	0.1213	-0.1604	0.3517	0.3836	0.603	-0.696	-0.1428	-0.1428	
MM109	0.0047	-0.0146	0.008	0.0322	0.0013	0.0128	0.0013	-0.0087	-0.0021	-0.0087	0.0013	0.0128	0.6261	0.0076	0.008	0.008	
MM111_1	-0.0691	-0.0184	-0.0898	0.1735	0.553	-0.0184	0.553	-0.0706	-0.0139	-0.1361	0.553	-0.0184	0.264	-0.1543	-0.0898	-0.0898	
MM111_2	-0.0505	-0.0148	-0.0963	0.1125	0.578	-0.0148	0.578	-0.1379	0.0507	-0.1494	0.578	-0.0148	0.9123	-0.1582	-0.1797	-0.1797	
MM002_1	-0.1335	0.6257	0.2811	-0.0595	0.6871	0.6257	0.6871	-0.2203	1.264	-0.2203	0.6871	0.6257	0.6017	-0.318	0.0575	0.0575	
MM002_2	-0.1495	0.6647	0.2746	-0.0312	0.701	0.6647	0.701	-0.1203	0.712	-0.2664	0.701	0.6647	0.6686	-0.2639	0.2746	0.2746	
MM002_3	-0.1329	0.6875	0.2698	-0.0464	0.7223	0.6875	0.7223	-0.1902	0.7139	-0.1902	0.7223	0.6875	0.6126	-0.243	0.2698	0.2698	
MM113_2	0.2578	0.0265	-0.0798	0.154	0.0744	0.0226	0.0744	-0.1065	0.0556	-0.1065	0.0744	0.0226	0.3615	-0.1403	-0.0798	-0.0798	

Supplementary Table S9. Aminopeptidase gene copy number variation scores from MM patient samples in the FIMM dataset (*n* = 169). (5/15)

SampleID	*AMZ1*	*ANPEP*	*LVRN*	*BLMH*	*AOPEP*	*CTSH*	*CTSV*	*DNPEP*	*DPP3*	*DPP4*	*DPP7*	*DPP8*	*DPP9*	*ENPEP*	*ERAP1*	*ERAP2*	Gene
MM003	0.7325	0.3259	0.113	0.4541	0.2261	0.3259	0.2124	-0.2642	0.1189	-0.3592	0.537	0.2865	1.0219	-0.4426	0.113	0.113	
MM004	0.0545	0.0488	-0.0433	0.0807	0.1456	0.0488	0.1456	-0.0862	0.1088	-0.0862	0.1456	0.0488	0.3105	-0.1081	-0.0433	-0.0433	
MM117_2	0.2983	0.5416	-0.138	0.1501	-0.079	0.5416	-0.079	-0.1525	0.0312	-0.1525	0.338	0.5381	1.1529	-0.2013	-0.138	-0.138	
MM124	-0.1004	-0.0278	-0.1013	0.1136	0.5313	-0.0278	0.5313	-0.0524	-0.0232	-0.1745	0.5313	-0.0278	0.2848	-0.1731	-0.1013	-0.1013	
MM129	0.2413	0.2802	-0.3826	0.2733	-0.239	0.0842	-0.239	0.0811	0.2177	-0.5826	0.6214	0.0842	0.8996	-0.6332	-0.3826	-0.3826	
MM130	-0.0141	0.0058	-0.0444	0.0949	0.0544	0.0194	0.0544	-0.0683	0.0142	-0.0683	0.0544	0.0194	0.2188	-0.0707	-0.0444	-0.0444	
MM005	0.5201	-0.2629	-0.3231	0.1038	-0.1704	0.6209	-0.1704	-0.4598	0.041	-0.4598	-0.1704	0.6209	0.2454	-0.3131	-0.3231	-0.3231	
MM135	0.0441	0.9663	-0.1464	0.0546	-0.0306	-0.0671	-0.0306	-0.1957	0.6273	-0.1957	-0.0306	0.5086	0.7857	-0.0031	-0.1464	-0.1464	
MM137	0.1897	0.5823	-0.4066	0.073	-0.2023	0.4094	-0.2023	-0.1328	0.644	-0.4491	0.0065	0.4094	0.8695	0.0795	-0.4066	-0.4066	
MM006	0.0227	0.0367	-0.0818	-0.7718	-0.0019	0.0367	0.0816	-0.0406	0.0466	-0.0406	0.0816	0.0367	0.2693	-0.0407	-0.0818	-0.0818	
MM007	-0.0148	0.6164	-0.1097	0.0697	-0.0157	-0.0153	-0.0157	-0.0198	0.0774	-0.1074	0.024	-0.0153	0.1767	-0.0999	-0.1097	-0.1097	
MM008	-0.0101	0.0084	-0.0956	0.1298	0.0238	0.0084	0.0238	-0.0666	0.0544	-0.0666	0.0238	0.0084	0.3049	-0.0856	-0.0956	-0.0956	
MM009	0.1082	0.1353	0.0978	-0.0124	0.0986	0.1353	0.0986	-0.0821	0.2385	-0.0307	0.0986	0.1795	0.1303	-0.0159	0.0978	0.0978	
MM010	-0.0482	0.039	0.2058	-0.007	0.4841	0.039	0.4841	-0.0936	0.4945	-0.0936	0.4841	0.039	0.5433	-0.2165	0.2058	0.2058	
MM011	0.0305	0.0471	-0.0283	0.1487	0.0461	0.0471	0.0461	-0.4058	0.0917	-0.4058	0.0461	0.0471	0.318	-0.5049	-0.0283	-0.0283	
MM014_4	0.16	0.3813	-0.1038	0.3585	0.3217	0.2807	0.3217	-0.0909	-0.1597	-0.079	0.2538	0.3237	0.2982	0.1093	-0.1038	-0.1038	
MM014_1	0.243	0.2847	-0.1078	0.3564	0.3272	0.2452	0.3272	-0.1873	-0.0748	0.0184	0.3272	0.2017	0.4229	0.1718	-0.1482	-0.1482	
MM014_2	0.1415	0.3019	-0.2341	0.3612	0.3553	0.3019	0.3553	-0.1372	-0.1333	-0.2489	0.3553	0.3019	0.4583	0.1961	-0.2341	-0.2341	
MM015	-0.0549	0.0492	-0.0672	0.1908	0.05	0.0492	0.05	-0.0965	0.0318	-0.0965	0.05	0.0492	0.3948	-0.1712	-0.0672	-0.0672	
MM017_1	-0.0455	0.3831	0.3532	-0.0548	0.3842	0.3831	0.3842	-0.1347	-0.0685	-0.1347	0.3842	0.3831	0.4671	-0.1565	0.3532	0.3532	
MM019	0.4115	0.6856	-0.1743	-0.0446	0.3691	0.6856	0.3691	-0.1561	0.6929	-0.1561	0.3691	0.6856	0.4928	-0.1725	-0.1743	-0.1743	
MM022_1	-0.045	-0.0158	-0.0259	0.0089	0.4026	-0.0158	0.4026	-0.0362	-0.045	-0.0362	0.3861	-0.0158	0.0452	-0.0233	-0.0259	-0.0259	
MM022_2	0.1465	0.0207	0.0586	0.0463	0.5682	-0.0802	0.5059	-0.0107	0.0851	0.1741	0.757	-0.022	0.1395	-0.0041	0.0133	0.0133	
MM022_3	0.1659	-0.0107	0.0248	0.017	0.5891	-0.0436	0.465	0.0182	0.1436	0.1648	0.8057	-0.0354	0.2289	-0.0247	0.0248	0.0248	
MM023	-0.1025	0.3982	0.362	-0.0587	0.3896	0.3982	0.3896	-0.1066	0.4066	-0.1066	0.3896	0.3982	0.4053	-0.1316	0.362	0.362	
MM024	-0.2619	0.2606	0.2022	0.0255	0.4131	0.2606	0.4131	-0.2247	0.4985	-0.3923	0.4131	0.2606	0.6312	-0.0266	0.2022	0.2022	
MM025_1	0.188	0.4527	0.363	0.0909	0.493	0.4527	0.493	-0.0344	0.8606	-0.2277	0.493	0.4381	0.7752	-0.263	0.363	0.363	
MM025_2	0.5189	0.5019	-0.4055	0.2049	-0.0786	0.5776	-0.0786	-0.1498	1.2452	-0.4147	0.9348	0.49	1.2281	-0.3619	-0.4055	-0.4055	
MM017_2	0.179	0.3687	0.2884	-0.0313	0.2823	0.3687	0.2823	-0.1541	0.0154	-0.1861	0.5636	0.3244	0.5963	-0.1914	0.2884	0.2884	

Supplementary Table S9. Aminopeptidase gene copy number variation scores from MM patient samples in the FIMM dataset (*n* = 169). (6/15)

SampleID	*F11*	*JMJD7*	*KDM8*	*LAP3*	*LNPEP*	*LTA4H*	*METAP1*	*METAP1D*	*METAP2*	*MMP14*	*NAALADL1*	*NPEPL1*	*NPEPPS*	*PEPD*	*PGPEP1*	Gene
MM032_1	-0.3369	0.307	0.0559	-0.3369	0.0338	-0.1106	-0.3369	-0.1779	-0.1106	-0.09	0.2072	0.0531	0.0684	0.4492	0.4492	
MM051_1	-0.1449	-0.0274	-0.0228	-0.0901	-0.0794	0.0134	-0.1449	-0.1014	0.0134	-0.0814	-0.0184	0.1072	0.045	0.0463	-0.0331	
MM053	0.1448	0.4823	-0.284	0.1403	0.5862	-0.0209	0.1448	0.1065	-0.0209	-0.0794	0.354	-0.1825	-0.2034	0.1773	0.0805	
MM056_1	-0.3045	0.5006	-0.325	-0.2914	0.0042	-0.307	-0.3229	0.1666	-0.307	-0.3148	0.1891	-0.3378	0.1582	0.4936	0.4936	
MM062	-0.3159	-0.0631	0.1158	0.0938	-0.4436	-0.3435	-0.327	-0.1857	-0.3435	-0.0176	0.038	0.0501	0.0648	0.0705	0.0705	
MM063	-0.0444	-0.0014	0.2443	-0.0444	-0.0198	-0.0012	-0.0444	-0.0184	-0.0012	-0.765	0.0434	0.0339	0.0444	0.0857	0.0857	
MM064	-0.1641	0.4296	-0.0036	-0.88	0.232	-0.0848	-1.0909	-0.1483	-0.0848	0.4262	-0.0781	-0.0305	-0.0141	0.0373	0.7849	
MM065	-0.7433	0.1105	0.0755	-0.7058	-0.0654	-0.0156	-0.7551	-0.0343	-0.0156	-0.0093	0.0091	0.003	0.0313	0.0839	0.7353	
MM066	-0.1092	-0.0156	0.1427	-0.1092	-0.065	-0.0168	-0.1092	-0.0711	-0.0168	-0.0048	0.0492	0.2483	0.0968	0.2075	0.2075	
MM067	-0.5	0.2568	-0.0687	-0.3379	-0.0112	-0.3408	-0.5339	-0.4561	-0.3408	0.1994	0.3213	-0.1512	0.368	0.6302	0.6302	
MM069	-0.1699	0.4124	0.0011	-0.1699	0.1396	-0.0982	-0.1699	-0.1456	-0.0982	-0.2709	0.2025	-0.0471	-0.097	0.3396	0.3396	
MM070	-0.1679	0.2227	-0.0837	-0.1679	0.1852	-0.1193	-0.1679	-0.1521	-0.1193	-0.1238	0.224	-0.0961	0.2508	0.2909	0.2909	
MM072	-0.3076	0.2574	-0.0852	-0.3076	0.2322	0.2103	-0.3076	-0.2604	0.2103	-0.2542	0.3	-0.1608	0.3314	0.4684	0.4684	
MM074	-0.0413	0.0057	0.0769	-0.0297	-0.0659	0.0407	-0.0297	-0.0326	0.0407	0.0164	-0.0049	-0.0102	0.0821	0.1239	0.1239	
MM075	-0.156	0.3682	-0.0464	-0.1393	0.3051	-0.1014	-0.1393	-0.1144	-0.1014	-0.0879	0.6632	-0.1768	-0.0427	0.4178	0.3941	
MM076	-0.1045	-0.0533	0.0006	-0.0684	-0.072	-0.0294	-0.1045	-0.0699	-0.0294	-0.0444	0.209	-0.0378	0.0076	0.2824	0.3156	
MM078_1	-0.1283	-0.0085	0.1011	-0.0817	-0.1099	0.009	-0.1369	-0.0801	0.009	-0.0264	0.0186	0.0377	0.0912	0.1703	0.1703	
MM079	-0.1274	-0.0466	0.5534	-0.1274	0.3454	-0.0405	-0.1274	-0.0936	-0.0405	-0.0492	0.4107	-0.0079	0.0487	0.5782	0.5782	
MM083_1	-0.052	0.0003	0.0778	-0.052	-0.0235	0.0026	-0.052	-0.0277	0.0026	-0.0064	0.0138	0.0184	0.0845	0.1676	0.1676	
MM085	-0.0228	0.2245	-0.1028	-0.0704	0.2631	-0.0786	-0.0228	-0.0645	-0.0786	-0.0508	0.2093	-0.1097	-0.0839	0.4283	0.4283	
MM086	-0.0972	0.0147	0.1977	-0.0988	-0.0333	0.0812	-0.0988	-0.1119	0.0812	0.0682	0.2094	0.0983	0.1831	0.1849	0.1849	
MM087	-0.0629	-0.0248	0.1006	-0.0618	-0.078	-0.0119	-0.1341	-0.1024	-0.0119	-0.0148	0.0119	0.0284	0.0883	0.184	0.5908	
MM088	-0.0277	0.0123	0.0922	0.0136	-0.011	-0.0053	-0.0588	-0.0489	-0.0053	0.4647	0.8516	0.0312	0.0765	0.1809	0.1809	
MM089	-0.1335	-0.0081	0.1532	-0.1188	-0.1051	0.0105	-0.1188	-0.0987	0.0105	0.006	0.1115	0.0335	0.1132	0.2357	0.2357	
MM090	-0.144	0.0014	0.1102	-0.0394	-0.1422	0.0136	-0.1855	-0.0743	0.0136	-0.0434	0.1405	0.0988	0.118	0.2254	0.2254	
MM093	-0.205	0.2714	0.0421	-0.1047	0.2178	-0.0888	-0.2088	-0.2267	-0.0888	-0.0973	-0.0358	-0.0321	0.0372	0.47	0.47	
MM094	-0.1741	0.7592	0.0059	-0.1131	0.368	-0.0806	-0.1741	-0.1292	-0.0806	-0.0992	0.4288	-0.0349	0.0048	0.591	0.591	
MM096	-0.0339	-0.0064	0.0532	-0.0339	-0.0289	0.0029	-0.0339	-0.0497	0.0029	0.0017	-0.0109	0.0026	0.0447	0.121	0.121	
MM100	-0.0823	0.0097	0.1372	-0.0049	-0.0316	0.0116	-0.0823	-0.051	0.0116	-0.8452	0.0097	0.0333	0.1027	0.2649	0.2649	
MM101	-0.2659	0.6357	-0.1412	-0.2659	0.2396	-0.233	-0.2659	-0.2698	-0.233	-0.2258	0.2984	-0.2009	-0.1441	0.4647	0.4647	
MM103	-0.2129	0.2846	0.0101	-0.2107	0.2489	-0.1045	-0.2107	-0.212	-0.1045	0.2879	0.3373	-0.0603	-0.0016	0.5754	0.5754	
MM104	-0.1475	-0.0072	0.1395	-0.2531	-0.1447	0.0608	-0.1403	-0.0835	0.0608	0.0082	0.0013	0.0315	0.1188	0.2609	0.7443	
MM105	-0.102	0.3065	0.1274	-0.102	-0.0816	-0.0078	-0.102	-0.1133	-0.0078	-0.038	0.3034	0.1666	0.0311	0.4943	0.4943	
MM108	-0.0817	0.0066	0.171	-1.03	-0.2104	-0.0102	-0.1598	-1.0938	-0.0102	-0.0319	0.1614	0.0349	0.1715	0.261	0.297	
MM110	-0.061	0.0094	0.0837	-0.0155	-0.0318	0.0064	-0.061	-0.0825	0.6025	0.0172	0.0193	0.0897	0.0866	0.1584	0.1736	

Supplementary Table S9. Aminopeptidase gene copy number variation scores from MM patient samples in the FIMM dataset (*n* = 169). 7/15)

SampleID	*F11*	*JMJD7*	*KDM8*	*LAP3*	*LNPEP*	*LTA4H*	*METAP1*	*METAP1D*	*METAP2*	*MMP14*	*NAALADL1*	*NPEPL1*	*NPEPPS*	*PEPD*	*PGPEP1*	Gene
MM113_1	-0.223	0.7301	0.0571	-0.1368	0.2975	-0.0789	-0.223	-0.1844	-0.0789	-0.111	0.719	-0.6716	0.0241	0.6314	0.6314	
MM114	0	0	0	0	0	0	0	0	0	0	0	0	0	0	0	
MM115	0.195	0	0	0.1371	0.1164	0	0.1371	0.1257	0	0	0	0	0	0	0	
MM116	-0.1387	0.0816	0.1169	-0.1194	0.0282	0.0644	-0.1194	-0.0735	0.0644	-0.0122	0.0074	0.0518	0.1085	0.305	0.3998	
MM117_1	0	0	0	0	0	0	0	0	0	0	0	0	0	0	0	
MM118	-0.1566	0.026	0.1174	-0.1058	-0.0863	0.0339	-0.1058	-0.131	0.0339	-0.014	0.0394	0.0809	0.1666	0.8383	0.8383	
MM119	0.0715	0.0563	0.3738	0.0715	0.081	0.0932	0.0715	-0.3246	0.0932	0.0474	-0.3445	-0.3705	0.0883	0.1542	0.112	
MM120	-0.086	0.0685	-0.0565	-0.086	0.1844	-0.1416	-0.086	-0.0789	-0.1416	-0.0655	0.1897	0.021	-0.0065	0.1242	0.1242	
MM121	-0.0599	0.0073	0.0838	-0.0487	-0.026	0.0175	-0.0487	-0.0447	0.0175	-0.0103	0.0147	0.0468	0.0861	0.2553	0.2553	
MM122	-0.0283	0.0021	0.0446	-0.0283	-0.0366	-0.0152	-0.0283	-0.0241	-0.0152	-0.0005	0.0126	0.0244	0.0493	0.1424	0.1424	
MM123	-0.1076	0.0132	0.1556	-0.0742	-0.0487	0.0225	-0.0742	-0.0608	0.0225	0.0316	0.0314	0.0702	0.1473	0.3272	0.3272	
MM126	-0.0258	-0.0086	0.0122	-0.0258	-0.0234	-0.0007	-0.0258	-0.0251	-0.0007	0.0027	0.0477	0.1218	0.0231	0.0628	0.0628	
MM128_1	-0.1022	0.0372	0.2042	-0.023	-0.0272	0.0689	-0.1022	-0.1478	0.0689	-0.0033	0.0602	0.1233	0.1801	0.3163	0.3163	
MM131	-0.1453	0.0157	0.182	-0.1421	-0.19	-0.2336	-0.1421	-0.1139	-0.2336	0.0509	0.0161	0.0641	0.1746	0.366	0.366	
MM132	-0.2075	0.4604	0.1092	-0.2075	-0.1447	-0.0184	-0.2075	-0.1767	-0.0184	-0.1551	0.4766	-0.7952	0.1408	0.8384	0.8384	
MM133	-0.2689	0.1732	0.3009	-0.1178	-0.2809	-0.0099	-0.2891	0.0682	-0.0099	0.4581	0.2439	-0.2597	0.2465	0.526	0.526	
MM134	0.1852	-0.0001	0.1674	-0.4392	-0.184	0.039	0.1852	-0.3238	0.039	-0.0418	0.067	0.0991	0.0241	0.2631	0.2631	
MM136	-0.1356	0.0291	0.2237	-0.0041	-0.1013	-0.0141	-0.1356	-0.1124	-0.0141	0.0338	0.0175	0.0592	0.1611	0.3639	0.3639	
MM138	-0.1907	0.1016	0.2149	-0.3096	-0.2605	0.0215	-0.2413	-0.3427	0.0215	-0.7869	0.284	-0.214	0.3762	0.5482	0.5482	
MM139	-0.1889	0.5354	0.4509	0.2558	-0.9595	-0.5267	-0.2695	0.0217	-0.5267	0.1653	0.304	-0.222	0.4011	0.597	0.597	
MM140	0.1905	-0.0828	0.0209	0.1905	-0.1433	-0.0389	0.1905	-0.1961	-0.0389	-0.0653	0.3173	-0.0676	0.0374	0.1595	0.1595	
MM013	-0.0419	-0.0037	0.0779	-0.0419	-0.028	-0.0066	-0.0419	-0.041	-0.0066	-0.8909	0.0005	0.0153	0.0542	0.1163	0.6527	
MM014_3	-0.0791	0.3829	0.0592	-0.0736	-0.5559	-0.2563	-0.0791	-0.242	-0.2563	-0.233	-0.067	-0.1827	0.4812	0.5809	0.7644	
MM016	-0.1775	-0.0816	0.0589	-0.1775	-0.1014	-0.1115	-0.1775	-0.146	-0.1115	-0.0334	-0.0202	0.0167	0.0335	0.473	0.473	
MM017_3	-0.3912	0.3557	-0.0134	-0.3415	-0.2302	-0.1679	-0.4785	-0.1749	-0.1679	-0.1491	-0.0726	0.0713	-0.02	-0.2404	0.449	
MM020	0.031	0.4379	-0.075	0.031	-0.1035	-0.1384	0.031	0.051	-0.1384	-0.3827	0.1189	-0.1163	-0.0923	0.1317	0.1377	
MM026	0.3137	0.3882	-0.0771	-0.1886	0.3503	-0.2236	0.3137	-0.2091	-0.2236	-0.157	0.4133	-0.138	0.4591	-0.0001	-0.0001	
MM027	-0.1586	0.0052	1.1304	-0.0567	-0.0607	-0.013	-0.153	-0.5181	-0.013	-0.0494	0.0627	0.0281	0.2363	0.2408	0.2408	
MM028	-0.1181	-0.0167	0.067	-0.0985	-0.0364	0.0719	-0.1177	-0.086	0.0719	-0.0091	0.0391	0.038	0.0907	0.1997	0.1997	
MM029	-0.0874	-0.0142	0.1057	-0.0874	-0.0524	0.0055	-0.0874	-0.117	0.0055	0.0137	0.0019	0.0194	0.1028	0.2235	0.2235	
MM030_1	-0.2722	0.1974	-0.0164	-0.2722	0.0967	-0.1467	-0.2722	0.2209	-0.1467	-0.1709	0.1878	-0.0491	0.1973	0.4361	0.4361	
MM030_2	-0.1998	0.1455	-0.0218	-0.1774	0.0176	-0.1776	-0.1774	0.1023	-0.1776	-0.1107	0.1082	-0.0677	0.1238	0.2979	0.2979	
MM031_2	-0.0137	0.0334	0.0147	0.0546	0.0287	-0.0532	0.0218	0.1736	-0.0532	0.0075	0.5985	0.0292	0.0414	0.0179	0.0179	
MM032_2	0.2611	0.2846	-0.1944	0.3143	0.2626	0.0147	0.2611	0.089	0.0147	-0.0747	0.0512	-0.2903	-0.1495	0.0718	0.0718	
MM033_4	-0.0593	0.4365	0.0374	-0.0593	-0.8908	-0.0236	-0.0593	-0.1052	-0.0236	-0.0188	-0.0078	0.0026	-0.0428	0.2827	0.2827	

Supplementary Table S9. Aminopeptidase gene copy number variation scores from MM patient samples in the FIMM dataset (*n* = 169). (8/15)

SampleID	*F11*	*JMJD7*	*KDM8*	*LAP3*	*LNPEP*	*LTA4H*	*METAP1*	*METAP1D*	*METAP2*	*MMP14*	*NAALADL1*	*NPEPL1*	*NPEPPS*	*PEPD*	*PGPEP1*	Gene
MM033_1	-0.0386	0.4588	-0.0922	-0.0595	-0.1065	-0.0301	-0.0595	-0.0389	-0.0301	-0.0188	-0.0508	-0.0543	-0.026	-0.0128	0.3495	
MM033_2	0.1236	0.4838	-0.0735	0.1162	-0.6202	0.0659	0.0923	0.1243	0.0659	-0.0144	-0.0277	-0.0601	-0.0279	0.1938	0.1938	
MM033_3	0.0063	0.5279	-0.027	0.0063	-0.87	0.047	0.0063	-0.0307	0.047	0.0312	-0.021	-0.022	-0.0246	0.304	0.304	
MM001	-0.0443	0.0652	-0.0269	-0.0443	0.0416	0.0418	-0.0443	0.0344	0.0418	-0.2494	0.0348	-0.0655	0.0385	0.0058	0.0058	
MM034_2	-0.0739	0.0255	0.1077	-0.0739	-0.0102	-0.0008	-0.0739	-0.0578	-0.0008	-0.0145	0.0221	0.0391	0.1108	0.2603	0.2603	
MM034_1	-0.2341	0.258	0.0447	-0.1504	-0.128	-0.2321	-0.38	-0.3068	-0.2321	-0.0396	0.0949	-0.126	0.0559	0.5217	0.5217	
MM035	-0.0808	-0.0099	0.1157	0.0564	-0.2842	0.0211	-0.1255	-0.8764	0.0211	-0.0249	0.0842	0.0881	0.1044	0.2826	0.2826	
MM036_1	-0.2247	-0.1136	0.04	0.2553	0.4098	-0.135	-0.2247	-0.1934	-0.135	-0.1145	-0.0925	-0.0705	1.0165	0.7059	0.7862	
MM036_2	-0.1171	-0.1228	-0.0006	-0.1171	0.3928	-0.0606	-0.1171	-0.1877	-0.0606	-0.1016	-0.0878	-0.073	1.0811	0.7548	0.7548	
MM037	-0.0738	0.0216	0.1435	-0.0332	-0.0706	-0.5576	-0.0893	-0.0637	-0.5576	0.0086	0.0318	-0.0014	0.1053	0.2159	0.2159	
MM038	-0.3455	0.0151	0.2821	-0.7488	0.1001	-0.8233	-0.373	-0.3864	-0.3493	0.0185	0.1958	-0.3545	0.8622	0.7691	0.9648	
MM039	-0.2097	0.4136	0.6175	-0.8585	-0.1896	0.0185	-0.9009	-0.1734	0.0185	-0.0573	0.5603	0.1047	0.177	0.7335	0.7335	
MM040	-0.0688	-0.0015	0.0825	-0.0688	-0.0369	0.0038	-0.0688	-0.0308	0.0038	0.0057	0.0058	0.0424	0.0802	0.116	0.116	
MM041	-0.1183	-0.0131	0.1979	-0.0264	-0.2723	-0.172	-0.1506	-0.1536	-0.172	-0.0382	0.0561	0.0659	0.1013	0.2258	0.2388	
MM042	-0.1784	-0.0139	-0.0427	-0.1854	0.3162	-0.0497	-0.1784	-0.1437	-0.0497	-0.0381	-0.0541	0.0674	0.4198	0.3924	0.3924	
MM043_1	-0.0175	-0.0027	0.0138	-0.0175	-0.0082	0.0007	-0.0175	-0.0174	0.0007	-0.0214	-0.0012	-0.0006	-0.0512	0.1041	0.1041	
MM043_2	-0.0391	0.2216	-0.0529	-0.0153	0.4691	0.3841	-0.0017	0.295	0.3841	-0.1021	-0.0559	-0.0149	0.0546	0.0019	0.0019	
MM044	-0.0519	0.079	0.0899	-0.0436	0.005	0.0397	-0.0436	-0.0259	0.0397	-0.2469	0.0132	0.0408	0.0305	0.1569	0.3262	
MM045	-0.0367	0.3384	-0.111	-0.0367	0.0194	-0.1262	-0.0367	-0.0168	-0.1262	-0.1332	0.2401	-0.086	0.0733	0.2831	0.2831	
MM046_1	0.0505	0.0072	-0.0185	0.0505	0.0223	0.013	0.0505	0.0302	0.013	0.0263	-0.0032	-0.0313	-0.0231	-0.0219	-0.0219	
MM046_2	0.0505	0.0072	-0.0185	0.0505	0.0223	0.013	0.0505	0.0302	0.013	0.0263	-0.0032	-0.0313	-0.0231	-0.0219	-0.0219	
MM047	-0.1992	0.3729	-0.06	-0.9158	0.3283	-0.1097	-0.1992	-0.155	-0.1097	-0.1027	0.7073	-0.0324	0.3415	0.562	0.562	
MM048_1	-0.1335	0.3767	-0.0461	-0.1335	0.2144	-0.0988	-0.1335	-0.129	-0.0988	-0.0489	0.3709	-0.0814	-0.0583	0.4753	0.4753	
MM048_2	-0.0789	0.3832	-0.1222	-0.1485	0.4845	-0.0409	-0.0738	0.1149	-0.1868	-0.1556	0.4026	-0.0425	-0.1238	0.4002	0.4002	
MM049	-0.2958	0.7859	-0.0544	-0.2958	0.262	-0.1174	-0.2958	0.0769	-0.1174	-0.1582	0.8338	-0.1319	0.0024	0.7398	0.7398	
MM050	-0.0059	0.0358	0.0855	-0.0059	0.0091	0.0232	-0.0059	-0.7303	0.0232	0.0172	0.0442	0.0653	0.0638	0.0966	0.0966	
MM051_2	0.011	0.1149	0.1008	0.0274	0.0387	0.1073	0.0551	0.1258	0.1073	0.0804	0.0631	0.1725	0.0735	0.0546	0.1528	
MM052_1	-0.1046	-0.0188	0.0504	-0.0548	-0.0898	-0.0147	-0.1046	-0.1132	-0.0147	-0.0223	0.1243	-0.0239	0.0442	0.1574	0.1574	
MM052_2	0.0404	-0.0839	-0.1355	-0.1463	0.023	0.0434	0.0404	0.0422	0.0434	-0.0447	-0.1676	-0.2957	-0.1051	-0.186	-0.186	
MM054	-0.141	0.2638	0.2243	-0.141	-0.0781	-0.0032	-0.141	-0.1767	-0.0032	-0.2759	0.199	0.1677	0.1634	0.3214	0.3214	
MM055	-0.2072	0.338	0.0804	-0.1911	0.204	-0.0558	-0.2038	-0.1592	-0.0558	0.2765	0.3277	-0.0586	0.0047	0.6352	0.6352	
MM056_2	-0.2136	0.6464	-0.2615	-0.2599	0.2105	-0.2098	-0.2136	0.0069	-0.2098	-0.2085	0.2867	-0.2768	-0.1695	0.1404	0.1404	
MM057_4	-0.1745	0.3765	-0.0457	-0.1745	0.0727	-0.1521	-0.1745	-0.1372	-0.1521	-0.1599	0.4527	-0.0575	0.1959	0.0744	0.0744	
MM057_5	-0.0811	-0.0793	0.083	-0.0811	-0.0345	-0.0725	-0.0811	-0.0396	-0.0725	-0.088	0.5647	0.034	0.2028	0.1709	0.1709	
MM057_6	-0.088	-0.0642	0.0559	-0.088	-0.0516	-0.0743	-0.088	-0.0568	-0.0743	-0.046	0.5786	0.0188	0.3116	0.1673	0.1673	

Supplementary Table S9. Aminopeptidase gene copy number variation scores from MM patient samples in the FIMM dataset (*n* = 169). (9/15)

SampleID	*F11*	*JMJD7*	*KDM8*	*LAP3*	*LNPEP*	*LTA4H*	*METAP1*	*METAP1D*	*METAP2*	*MMP14*	*NAALADL1*	*NPEPL1*	*NPEPPS*	*PEPD*	*PGPEP1*	Gene
MM057_1	-0.1145	0.0112	0.013	-0.0559	-0.014	-0.0406	-0.1145	-0.0717	-0.0406	-0.0405	0.5189	0.0325	0.0313	0.1452	0.1452	
MM057_2	-0.1156	-0.0113	0.0963	-0.1156	-0.0569	-0.0088	-0.1156	-0.0825	-0.0088	-0.032	0.0728	0.041	0.0808	0.1843	0.1843	
MM057_3	-0.2286	0.3885	0.0448	-0.2286	0.3525	-0.2011	-0.2286	-0.1648	-0.2011	-0.1915	0.5219	-0.0045	0.145	0.3634	0.3634	
MM058_1	-0.0578	0.0781	0.1436	0.7624	-0.1123	-0.5776	-0.0578	0.0464	-0.5776	-0.5807	0.0919	0.1165	0.1417	0.2239	0.2239	
MM058_2	-0.9805	1.9347	1.0508	-0.9946	-0.306	-0.5019	-0.9946	-0.3445	-0.5019	-0.613	0.9038	0.48	0.5254	1.4564	1.7752	
MM059	-0.1404	-0.0174	0.1457	-0.0659	-0.0529	-0.0292	-0.1404	-0.0618	-0.0292	-0.0306	0.134	0.074	0.1338	0.1859	0.2991	
MM060	-0.0597	-0.0209	0.1291	-0.0089	-0.1357	-0.0441	-0.0089	-0.1772	-0.0441	-0.0182	0.0789	0.0462	0.107	0.2983	0.2983	
MM061_1	-0.0371	0.0008	0.0352	-0.013	-0.0279	0.0033	-0.0371	-0.0353	0.0033	-0.0112	-0.0096	-0.0079	0.1149	0.5168	0.5168	
MM061_2	-0.022	-0.0008	0.0328	0.0135	-0.0331	-0.0215	-0.022	-0.0307	-0.0215	-0.0329	-0.018	-0.0472	0.0611	0.5917	0.5917	
MM068_1	-0.0449	0.0184	0.1239	-0.0449	-0.0157	0.0115	-0.0449	-0.0195	0.0115	0.0161	0.2311	0.0538	0.0777	0.1328	0.1328	
MM068_2	-0.748	0.0381	-0.1203	-0.4979	-0.2202	-0.0456	-0.748	-0.3739	-0.0456	-0.2533	0.9251	0.4759	0.3094	0.4795	0.9993	
MM071	-0.3458	0.6	-0.0239	-0.3458	-0.2244	-0.1877	-0.3458	-0.319	-0.1877	-0.1916	0.5388	0.3724	0.0203	0.7728	0.7728	
MM073	-0.1392	0.4311	0.0463	-0.1392	0.2984	-0.0937	-0.1392	-0.1961	-0.0937	-0.0839	0.4375	-0.0863	0.0338	0.4495	0.4495	
MM077	0.1157	-0.0099	0.0544	0.1157	-0.1914	0.0046	0.1157	-0.2574	0.0046	-0.2934	-0.0001	0.2604	0.0525	0.0943	0.0943	
MM078_2	-0.0819	-0.001	0.114	-0.0819	-0.0397	0.0047	-0.0819	-0.0826	0.0047	-0.0162	0.0073	0.0352	0.1041	0.1979	0.1979	
MM080	-0.0889	0.0348	0.093	-0.1019	-0.0654	-0.0213	-0.1019	-0.0957	-0.0213	-0.0157	0.0121	0.0132	0.073	0.204	0.204	
MM081	-0.1538	0.1426	0.2541	-0.0603	-0.1872	-0.1569	-0.2404	-0.2652	-0.1569	0.1146	0.2884	0.1717	0.3996	0.3581	0.7106	
MM082	-0.2629	0.8208	-0.0377	-0.2629	0.3097	-0.1517	-0.2629	-0.2364	-0.1517	-0.1495	0.4558	-0.0989	-0.0529	0.6772	0.6772	
MM083_2	-0.0794	0.0205	0.08	-0.0461	-0.0274	0.0346	-0.0461	-0.06	0.0346	-0.0147	0.0243	0.0101	0.096	0.1651	0.1651	
MM084	-0.1637	0.3773	-0.1016	-0.1637	0.3621	-0.127	-0.1637	-0.172	-0.127	-0.1243	0.374	-0.1383	-0.0934	0.4263	0.4831	
MM091	-0.2541	0.3971	0.0606	-0.067	0.2523	-0.1111	-0.263	-0.1797	-0.1111	-0.112	0.4275	-0.0386	0.0161	0.119	0.119	
MM092	-0.035	-0.0037	0.1445	-0.0273	-0.1218	-0.0107	-0.0648	-0.0364	-0.0107	-0.004	0.0018	0.0272	0.0474	0.0964	0.0964	
MM095	-0.154	-0.022	0.1141	-0.018	-0.1058	-0.8423	-0.1701	-0.1312	-0.8423	-0.064	0.026	0.0426	0.0608	0.2101	0.2101	
MM097	-0.1127	0.0895	0.1233	-0.1127	-0.1391	-0.0247	-0.1127	-0.1512	-0.0247	-0.0165	0.222	0.1894	0.0918	0.2963	0.4534	
MM098	0.1593	-0.6245	-0.0499	0.2239	0.1084	-0.018	0.2239	0.1179	-0.018	0.0162	0.0083	-0.0146	-0.121	-0.7449	-0.7449	
MM099	-0.2773	0.239	0.1789	-0.4757	-0.1354	-0.9541	-0.563	-1.0329	-0.3051	-0.0315	0.0756	0.0807	0.1549	0.3856	0.3856	
MM102	-0.0949	0.0001	0.1146	-0.0949	0.0038	-0.0317	-0.0949	-0.0739	-0.0317	0.0082	0.0005	0.0107	0.1086	0.2785	0.2785	
MM107	-0.1382	0.3836	0.5043	-0.1209	-0.1428	0.0001	-0.696	-0.1604	0.0001	-0.0665	0.1213	-0.0337	0.4861	0.1038	0.6388	
MM109	0.0076	0.0128	0.0167	0.0076	0.008	0.0099	0.0076	-0.0087	0.0099	0.0269	-0.0021	-0.0033	0.0479	0.0632	0.0632	
MM111_1	-0.1543	-0.0184	0.6783	-0.0991	-0.0898	-0.0633	-0.1543	-0.1361	-0.0633	-0.0324	-0.0139	0.0049	0.1735	0.1806	0.264	
MM111_2	-0.1545	-0.0148	0.728	-0.1582	-0.1797	-0.0148	-0.1582	-0.1494	-0.0148	-0.0146	0.0507	0.0417	0.4597	0.2625	0.8396	
MM002_1	-0.2783	0.6257	0.1501	-0.1779	0.0575	-0.1737	-0.318	-0.2203	-0.1737	-0.1064	0.7339	-0.0189	0.03	0.6017	0.6017	
MM002_2	-0.2747	0.6647	0.1333	-0.1424	0.2746	-0.0963	-0.2639	-0.2664	-0.0963	-0.1254	0.712	-0.0445	0.0313	0.5755	0.6686	
MM002_3	-0.243	0.6875	0.1381	-0.1853	0.2698	-0.1156	-0.1853	-0.1902	-0.1156	-0.1108	0.7139	-0.0372	0.0179	0.6126	0.6126	
MM113_2	-0.1403	0.0226	0.1698	-0.1403	-0.0798	-0.0036	-0.1403	-0.1065	-0.0036	-0.0099	0.0556	0.0826	0.1397	0.3615	0.3615	

Supplementary Table S9. Aminopeptidase gene copy number variation scores from MM patient samples in the FIMM dataset (*n* = 169). (10/15)

SampleID	*F11*	*JMJD7*	*KDM8*	*LAP3*	*LNPEP*	*LTA4H*	*METAP1*	*METAP1D*	*METAP2*	*MMP14*	*NAALADL1*	*NPEPL1*	*NPEPPS*	*PEPD*	*PGPEP1*	Gene
MM003	-0.3524	0.2865	0.0854	-0.0989	0.113	0.0752	-0.4148	-0.3592	0.0752	-0.1073	0.0595	-0.2625	0.4261	0.1153	1.026	
MM004	-0.1081	0.0737	0.0433	-0.012	-0.0433	0.0548	-0.1081	-0.0862	0.0548	-0.0239	0.1088	-0.0085	0.0807	0.3105	0.3105	
MM117_2	-0.2306	0.5381	0.2282	-0.2013	-0.138	-0.08	-0.2013	-0.1525	-0.08	-0.9283	0.0312	0.1396	0.258	0.317	0.5582	
MM124	-0.1731	-0.0278	0.0918	0.4806	-0.1013	-0.0248	-0.1731	-0.1745	-0.0248	-0.0071	-0.0232	-0.0116	0.1136	0.2848	0.2848	
MM129	-0.545	0.1688	0.4688	-1.3921	-0.3826	-0.0563	-0.6332	-0.2081	-0.0563	0.3428	0.2177	0.3963	0.4344	0.6179	0.9567	
MM130	-0.1123	0.0194	0.0409	-0.0707	-0.0444	0.0706	-0.0707	-0.0683	0.0706	-0.0073	0.0142	0.0274	0.116	0.2188	0.2188	
MM005	-0.3131	0.6209	0.0416	-0.7536	-0.3231	-0.1285	-0.7928	-0.4598	-0.1285	0.3233	0.041	0.1433	-0.1929	0.2454	0.2454	
MM135	-0.1542	-0.0563	0.1262	-0.1142	-0.1464	-0.0355	-1.1616	-0.1957	-0.0355	-0.0374	0.6273	0.0097	0.0546	0.7857	0.7857	
MM137	0.1252	0.4723	0.0877	0.3274	-0.4066	0.0567	0.0795	-0.4491	0.0567	-0.0728	0.644	-0.0063	0.2212	0.8695	0.8695	
MM006	-0.0407	0.0367	0.1764	-0.0407	-0.0818	-0.8002	-0.0407	-0.0406	-0.8002	-0.8504	0.0466	0.0525	0.1512	0.2693	0.2693	
MM007	-0.0999	-0.0149	0.0687	-0.0638	-0.1097	0.0385	-0.0999	-0.1074	0.0385	0.0371	0.0774	0.016	0.0575	0.1348	0.1348	
MM008	-0.0856	0.0084	0.1362	-0.0856	-0.0956	0.01	-0.0856	-0.0666	0.01	0.0099	0.0544	0.0285	0.1373	0.3049	0.3049	
MM009	-0.0273	0.1795	-0.1487	-0.0459	0.0978	-0.0165	-0.0159	-0.0307	-0.0165	-0.0356	0.2385	-0.0614	-0.0338	0.1303	0.1303	
MM010	-0.2165	0.039	1.1137	-0.1235	0.2058	-0.0694	-0.2165	-0.0936	-0.0694	-0.0815	0.5112	0.4566	-0.007	0.5433	0.5433	
MM011	-0.1385	0.0057	0.1765	-0.149	-0.0283	0.0184	-0.5099	-0.4058	0.0184	-0.1273	0.0917	0.0867	0.1234	-0.1037	0.318	
MM014_4	0.1612	0.3063	-0.1055	0.1448	-0.1038	-0.1382	0.1093	-0.079	-0.1382	-0.1545	-0.1597	-0.142	0.5374	0.2511	0.2511	
MM014_1	0.2806	0.3258	-0.0474	0.3027	-0.1482	-0.135	0.2653	-0.1533	-0.135	-0.1706	-0.0748	-0.1062	0.3286	0.3095	0.3095	
MM014_2	0.1961	0.319	-0.0242	0.2075	-0.2341	-0.143	0.1961	-0.2489	-0.143	-0.1747	-0.1333	-0.1233	0.3531	0.4583	0.4583	
MM015	-0.1712	0.0047	0.1729	-0.0835	-0.0672	0.0582	-0.1712	-0.0965	0.0582	-0.1631	0.0318	0.1049	0.1827	0.2473	0.4304	
MM017_1	-0.1565	0.3831	-0.053	-0.1565	0.3532	-0.1213	-0.1565	-0.1347	-0.1213	-0.1112	-0.0685	-0.0758	-0.0548	-0.0466	0.4671	
MM019	-0.1725	0.6856	-0.0168	-0.1725	-0.1743	-0.1082	-0.1725	-0.1561	-0.1082	-0.1181	0.7521	-0.0706	-0.0964	0.4928	0.4928	
MM022_1	-0.0233	-0.0158	0.0082	-0.0233	-0.0259	-0.0005	-0.0233	-0.0362	-0.0005	0.0018	-0.045	-0.0369	0.0157	0.0452	0.0452	
MM022_2	-0.0092	0.0385	0.0591	-0.0924	0.0133	-0.048	0.0192	0.0152	-0.048	-0.0788	0.0851	0.0329	0.0223	0.0932	0.0932	
MM022_3	-0.0328	0.0603	0.0733	-0.0861	0.0248	-0.0603	0.0028	0.1648	-0.0603	-0.0736	0.1436	0.0719	0.0512	0.0701	0.0701	
MM023	-0.1146	0.3937	-0.0707	-0.1316	0.362	-0.0928	-0.1316	-0.1066	-0.0928	-0.1024	0.4066	-0.0689	-0.0676	0.3808	0.4053	
MM024	-0.0016	0.3156	0.0656	0.1591	0.2022	-0.1217	-0.0266	-0.3923	-0.1217	-0.2258	0.4985	0.3644	-0.2571	0.6312	0.6312	
MM025_1	-0.2213	0.4381	0.1397	-0.1861	0.363	-0.1938	-0.263	-0.2277	-0.1938	-0.1049	0.8606	0.0086	0.0909	0.7752	0.7752	
MM025_2	-0.3325	0.5526	0.3544	-0.292	-0.4055	-0.2743	-0.3619	-0.1498	-0.2743	0.1615	1.2062	-0.0133	0.2687	0.8649	1.1099	
MM017_2	-0.231	0.3244	0.0329	-0.1914	0.2884	-0.1332	-0.1914	0.09005	-0.1332	-0.1263	0.0154	-0.0362	-0.0313	0.0616	0.5963	

Supplementary Table S9. Aminopeptidase gene copy number variation scores from MM patient samples in the FIMM dataset (*n* = 169). (11/15)

SampleID	*RNPEP*	*RNPEPL1*	*TPP1*	*TPP2*	*TRHDE*	*XPNPEP1*	*XPNPEP2*	*XPNPEP3*	Gene
MM032_1	-0.0563	-0.1779	0.0612	-0.2732	-0.1106	-0.1331	-0.1972	0.119	
MM051_1	-0.2143	-0.1009	-0.0123	-0.8163	0.0134	-0.0844	0.0457	-0.717	
MM053	-0.044	-0.278	0.3962	-0.737	-0.0209	-0.1053	-0.7828	-1.0814	
MM056_1	0.5174	-0.3571	0.1584	-0.3176	-0.307	-0.311	-1.0951	-0.2825	
MM062	-0.1559	0.0965	0.0457	-0.0479	-0.4272	-0.0999	-0.1817	0.1054	
MM063	0.0042	-0.0184	0.0434	-0.8516	-0.0012	0.0002	-0.0066	0.0597	
MM064	-0.0722	-0.1483	-0.1149	-1.0506	-0.0848	-1.0534	0.2991	-0.9027	
MM065	0.5944	-0.0343	0.0091	-0.0361	-0.0156	-0.0223	-0.0325	-0.6622	
MM066	0.1976	-0.0711	0.0492	-0.0849	-0.0168	-0.014	-0.0872	0.1524	
MM067	-0.2727	-0.2913	0.3213	-0.4285	-0.3408	-0.2138	-0.3477	0.02	
MM069	-0.0961	-0.1456	0.2025	-0.1476	-0.0982	-0.1023	-0.1364	0.0078	
MM070	-0.1286	-0.1521	0.224	-0.6129	-0.1193	-0.1225	-0.1337	-0.0699	
MM072	-0.2557	-0.2604	0.3503	-1.0694	0.2103	-0.2307	-0.2494	-0.085	
MM074	0.014	-0.0326	-0.0049	-0.0455	0.0407	0.003	-0.5881	0.0742	
MM075	-0.0604	-0.1144	0.4198	-0.1353	-0.1014	-0.0919	1.1492	-0.0025	
MM076	-0.0634	-0.0699	0.209	-0.0895	-0.0294	-0.0646	-0.2389	-0.0146	
MM078_1	-0.0537	-0.0801	0.3935	-0.1141	0.009	-0.0216	-0.0237	-0.249	
MM079	-0.0615	-0.0936	0.43	-0.1245	-0.0405	-0.0612	-0.0756	0.0715	
MM083_1	0.0038	-0.0277	0.0138	-0.0745	0.0026	-0.0144	-0.0153	0.0729	
MM085	-0.063	-0.0645	0.2093	0.2425	-0.0786	0.2047	0.0007	-0.1105	
MM086	-0.0388	0.0291	0.1233	-1.0137	-0.1903	-0.0445	0.644	0.2078	
MM087	0.6966	-0.0267	0.0119	-1.0018	-0.0119	-0.0374	-0.0442	0.0938	
MM088	0.0258	0.0083	0.078	-0.2916	-0.0053	0.0207	-0.1596	0.0981	
MM089	0.3101	-0.0173	-0.0056	-0.1059	0.0105	-0.0196	-0.0263	0.1265	
MM090	-0.0606	-0.0743	0.036	-0.9876	0.0136	-0.0199	-0.021	-0.7271	
MM093	0.2632	-0.2267	-0.0358	-0.6947	-0.0888	-0.1273	-0.1101	0.0392	
MM094	-0.0661	-0.1292	0.4288	-0.1555	-0.0806	-0.1012	-0.9346	-0.0017	
MM096	-0.0407	-0.0115	-0.0109	-0.0523	0.0029	-0.0109	0.0061	0.0369	
MM100	-0.009	-0.051	0.0097	-0.9103	0.0116	0.0006	-0.0421	0.0881	
MM101	0.47	-0.2698	0.2684	-0.277	-0.233	-0.2321	0.6051	-0.1391	
MM103	-0.1415	-0.0765	0.3373	-0.2032	-0.1045	-0.1305	-0.1611	0.0118	
MM104	0.2153	-0.0835	0.0013	-0.1234	-0.1614	-0.0971	-0.39	0.669	
MM105	-0.0547	-0.0861	0.3034	-0.6075	-0.0078	-0.1031	-0.0745	0.0222	
MM108	0.4949	-0.9191	-0.0068	-1.0758	-0.0102	-0.1091	-0.0985	0.1459	
MM110	0.0227	-0.0084	0.0031	-0.9823	-0.0109	0.0014	-0.9551	-0.7909	

Supplementary Table S9. Aminopeptidase gene copy number variation scores from MM patient samples in the FIMM dataset (*n* = 169). (12/15)

SampleID	*RNPEP*	*RNPEPL1*	*TPP1*	*TPP2*	*TRHDE*	*XPNPEP1*	*XPNPEP2*	*XPNPEP3*	Gene
MM113_1	-0.122	-0.1844	0.719	-0.2125	-0.0789	-0.1356	-0.1766	0.0312	
MM114	-0.0736	0	0	0	0	0	0	0	
MM115	0.0807	0.1257	0	0	0	0	0	0	
MM116	-0.0691	-0.0735	0.0074	-0.216	0.0644	-0.0262	-0.0668	0.1385	
MM117_1	0	0	0	0	0	0	0	0	
MM118	0.4444	-0.0267	0.0394	-0.8306	0.0339	-0.0367	-0.1215	-0.1527	
MM119	0.6433	-0.3463	-0.359	-0.9162	0.0932	0.0707	-0.8987	-0.8858	
MM120	-0.0734	-0.0789	0.1897	0.0027	-0.1416	-0.0785	-0.134	0.0051	
MM121	-0.0047	-0.0447	0.0147	-0.0517	0.0175	-0.0269	-0.5142	0.1026	
MM122	-0.0113	-0.0241	0.0126	0.0307	-0.0152	-0.0179	-0.0254	0.0375	
MM123	0.0135	-0.0608	0.0314	-0.0891	0.0225	-0.0363	-0.826	0.1677	
MM126	-0.0079	-0.0251	0.0477	-0.698	-0.0007	-0.0169	-0.704	0.0085	
MM128_1	0.0324	-0.1478	0.0602	-1.0383	0.0689	0.0119	0.9835	-0.7839	
MM131	-0.038	-0.1139	0.0161	-0.1127	-0.2336	-0.0235	-0.0698	0.1979	
MM132	-0.1051	-0.1767	0.4162	-0.3598	-0.0184	-0.0732	-0.1518	0.1428	
MM133	-0.1915	-0.0721	-0.2162	-0.2522	-0.3627	-0.2467	-0.0584	0.4426	
MM134	0.398	-0.3238	0.0635	-0.4074	0.039	-0.0375	0.9132	-0.0959	
MM136	-0.0744	-0.0266	0.0175	-0.0951	-0.0141	-0.0152	-0.0585	0.1663	
MM138	0.0256	-0.0018	-0.2376	-1.0418	-0.3886	-0.1687	0.1089	0.2873	
MM139	-0.153	0.0017	-0.1849	-0.7072	-1.0014	-0.0778	-0.7741	-0.2262	
MM140	-0.1211	-0.0967	0.2812	-0.1776	-0.0389	-0.1174	-0.1085	0.0454	
MM013	0.5046	-0.041	0.0495	-0.9052	-0.0066	-0.0257	-0.0351	0.0603	
MM014_3	0.2009	-0.3347	-0.0189	-1.1993	-0.2563	-0.165	-0.3915	0.1047	
MM016	0.2859	-0.1162	-0.0202	-0.7106	-0.1115	-0.0561	-0.1164	0.0956	
MM017_3	0.7527	-0.1749	-0.0389	-0.9325	-0.1679	-0.0528	-1.0394	0.079	
MM020	0.0101	0.1661	0.1189	-0.1779	-0.1384	-0.108	-0.8735	-0.071	
MM026	0.3683	-0.2091	0.3955	-1.1479	-0.2236	0.3529	-0.183	-0.0809	
MM027	-0.0226	-0.5181	-0.0092	-0.151	-0.013	-0.0334	-0.0395	0.2242	
MM028	-0.0335	-0.086	0.0391	-0.117	-0.0171	-0.0217	0.0344	0.107	
MM029	0.5028	-0.0412	0.0019	-0.0892	0.0055	-0.0526	-0.9201	0.0864	
MM030_1	0.1005	-0.2386	0.1878	-0.7179	-0.1467	-0.0333	-0.2161	-0.0096	
MM030_2	0.1254	-0.1673	-0.0753	-0.4588	-0.1776	-0.0749	-0.1855	-0.1074	
MM031_2	-0.0761	0.1192	0.57	0.0203	-0.0532	0.0114	0.1324	0.0559	
MM032_2	-0.0397	-0.2131	0.0512	0.1386	0.0147	-0.0746	-0.3739	-0.2384	
MM033_4	0.3517	-0.1052	-0.0078	-0.8056	-0.0236	-0.0158	-0.0879	0.0753	

Supplementary Table S9. Aminopeptidase gene copy number variation scores from MM patient samples in the FIMM dataset (n = 169). (13/15)

SampleID	*RNPEP*	*RNPEPL1*	*TPP1*	*TPP2*	*TRHDE*	*XPNPEP1*	*XPNPEP2*	*XPNPEP3*	Gene
MM033_1	0.408	-0.126	-0.11	-0.1835	-0.0301	-0.0598	-0.0249	-0.0358	
MM033_2	-0.1109	-0.0992	-0.0435	-0.1881	0.0659	-0.0361	-0.0159	-0.0416	
MM033_3	-0.0539	-0.0307	-0.021	-0.7625	0.047	0.0028	-0.0506	-0.0064	
MM001	0.4555	0.0344	0.0348	-0.6172	0.0418	0.0513	-0.6255	0.0358	
MM034_2	-0.0248	-0.0578	0.0221	-0.0765	-0.0008	-0.0357	-0.115	0.1253	
MM034_1	-0.2225	-0.1034	0.205	-1.4194	-0.2321	-0.1284	-0.4236	0.0759	
MM035	0.4125	-0.7303	0.0468	-0.8487	0.0211	-0.0035	-0.0314	-0.6312	
MM036_1	0.8143	-0.0845	-0.1295	-0.2423	-0.135	-0.1732	-0.2118	-0.0148	
MM036_2	0.8075	-0.132	-0.0878	-0.2482	-0.0943	-0.145	-0.1644	-0.0448	
MM037	0.7356	-0.0153	-0.7429	-0.0666	-0.2847	-0.0684	-0.5395	0.1213	
MM038	0.9235	-0.1153	-0.3315	-0.7546	-0.5287	-0.268	-0.6532	0.1954	
MM039	-0.1415	0.2082	0.6937	-0.2041	0.0185	-0.7253	-0.127	0.0867	
MM040	-0.0051	-0.0308	0.0058	-0.0505	0.0038	-0.0099	-0.0655	0.0819	
MM041	-0.0228	-0.0462	0.0164	-0.1183	-0.172	0.024	0.0293	0.144	
MM042	-0.025	-0.2057	-0.1206	-0.1884	-0.0497	-0.1577	-0.1423	-0.062	
MM043_1	0.5102	-0.017	-0.0012	-0.8943	0.0007	-0.0113	0.0234	0.007	
MM043_2	0.8661	-0.0535	0.0663	-0.7204	0.1703	-0.0049	1.8061	-0.0632	
MM044	0.1442	-0.0259	0.0132	-0.1917	0.0397	-0.015	-0.4619	0.0757	
MM045	-0.1312	0.0114	0.2401	-0.2085	-0.1262	-0.1374	-0.1388	-0.002	
MM046_1	0.0144	0.0302	-0.0032	0.048	0.013	0.009	-0.0118	-0.0659	
MM046_2	0.0144	0.0302	-0.0032	0.048	0.013	0.009	-0.0118	-0.0659	
MM047	-0.1206	-0.155	0.4042	-0.2918	-0.1097	-0.115	0.3639	0.0168	
MM048_1	0.0925	-0.129	0.3709	-0.0847	-0.0988	-0.0932	-0.1368	-0.0585	
MM048_2	0.4142	-0.0474	0.4592	-0.0445	-0.2203	-0.0585	-0.1624	-0.0666	
MM049	-0.1937	-0.2552	0.8338	-0.3187	-0.1174	-0.1629	-0.2861	0.0299	
MM050	0.4439	-0.7303	0.0442	-0.7335	0.0232	0.0212	0.012	-0.6484	
MM051_2	0.0797	0.0344	0.0559	-0.4631	0.1073	0.1008	0.0598	-0.4233	
MM052_1	0.8025	-0.0594	0.1243	-0.1141	-0.0147	-0.0779	-0.0705	0.0369	
MM052_2	0.7922	-1.6292	-0.3316	0.0113	0.0434	-0.0858	-0.1824	-0.3175	
MM054	0.7792	-0.0144	0.199	-0.9322	-0.0032	0.0314	-0.8713	0.2932	
MM055	-0.1085	-0.1592	0.3277	-0.3184	-0.0558	-0.2399	-0.0565	0.0172	
MM056_2	0.482	0.0069	0.2867	-0.1841	-0.2098	-0.2183	-1.0603	-0.3081	
MM057_4	0.1853	-0.1372	0.4664	-0.207	-0.1521	-0.1672	-0.2744	0.6007	
MM057_5	0.0654	-0.0396	0.5647	-0.1365	-0.0725	-0.0727	-0.1651	0.7727	
MM057_6	-0.0217	-0.0568	0.5786	-0.116	-0.0743	-0.0792	-0.1635	0.8145	

Supplementary Table S9. Aminopeptidase gene copy number variation scores from MM patient samples in the FIMM dataset (*n* = 169). (14/15)

SampleID	*RNPEP*	*RNPEPL1*	*TPP1*	*TPP2*	*TRHDE*	*XPNPEP1*	*XPNPEP2*	*XPNPEP3*	Gene
MM057_1	-0.0064	-0.0717	0.5189	-0.0838	-0.0406	-0.0489	-0.0294	0.6739	
MM057_2	-0.0145	-0.0825	0.0728	-0.1013	-0.0328	-0.0385	-0.0543	0.1328	
MM057_3	-0.0798	-0.1648	0.5219	-0.2765	-0.2011	-0.1323	-0.2703	0.6612	
MM058_1	0.0731	0.0464	0.0919	-0.6164	-0.5776	0.0769	0.0248	0.167	
MM058_2	0.0854	-0.3445	0.1263	-1.0787	-0.5019	0.0814	-0.5304	1.0895	
MM059	0.5127	-0.0618	0.134	-1.0174	-0.0292	-0.0118	-0.9656	0.1707	
MM060	0.7657	-0.2249	-0.01	-0.8266	-0.0441	-0.0887	-0.0983	-0.4831	
MM061_1	0.7919	-0.0353	-0.0096	-0.7994	0.0033	-0.0169	0.7339	0.0399	
MM061_2	0.8234	-0.0222	-0.018	-0.8536	-0.0215	-0.0049	0.7615	0.0032	
MM068_1	0.0175	-0.0195	0.0034	-0.6616	0.0115	0.0094	-0.0103	0.101	
MM068_2	-0.0408	-0.3739	0.1883	-1.2745	-0.0456	-0.0929	-0.4277	0.6394	
MM071	-0.1246	-0.319	0.361	-1.3228	-0.1877	-0.2335	-0.4027	-0.0234	
MM073	-0.142	-0.1184	0.4375	-0.1779	-0.0937	-0.0881	-0.1339	-0.8406	
MM077	0.686	-0.2574	-0.0001	-0.9348	0.0046	-0.5268	-0.9031	0.0819	
MM078_2	-0.0338	0.0297	0.4764	-0.0714	0.0047	-0.0114	-0.061	-0.6757	
MM080	0.9231	-0.0957	0.0121	-1.0474	-0.0213	-0.0353	-0.0747	0.0704	
MM081	1.2053	0.0482	-0.1463	-1.0599	-0.1569	0.0434	-1.0406	0.3612	
MM082	-0.2119	-0.2364	0.4558	-0.2621	-0.1517	-0.1663	-1.1993	-0.0263	
MM083_2	-0.0138	-0.0099	0.0058	-0.102	0.0346	-0.0169	-0.0188	0.0583	
MM084	-0.1386	-0.172	0.374	-1.0143	-0.127	0.1812	-0.02	-0.1161	
MM091	0.0087	-0.1797	0.4275	-0.1762	-0.1111	-0.1207	-0.1072	0.0725	
MM092	0.003	-0.0364	0.0018	-0.0428	-0.0107	-0.0208	-0.0064	0.058	
MM095	0.2172	-0.0063	0.026	-0.0808	-0.8423	-0.0336	-0.0445	0.121	
MM097	0.0339	-0.1512	-0.025	-0.1676	-0.0247	-0.127	-0.3138	0.1085	
MM098	-0.4263	0.9029	0.0083	0.1294	0.0372	0.0561	0	0.8092	
MM099	0.5016	0.0199	0.1211	-1.0318	-0.3051	-0.8589	-0.2012	-0.5826	
MM102	0.0006	-0.0739	-0.0119	-0.1219	-0.0317	-0.0175	-0.1071	0.091	
MM107	-0.0736	-0.096	0.1213	-0.1707	-0.0888	-0.0683	-0.8168	0.0266	
MM109	0.9477	-0.0087	-0.0021	-0.9318	0.0099	0.0049	-0.0356	-0.8781	
MM111_1	1.186	-0.0706	-0.0139	-1.0091	-0.0633	-0.1148	-0.0641	0.0914	
MM111_2	0.8819	-0.0499	0.0507	-1.0777	-0.0148	-0.1189	-0.1436	0.1493	
MM002_1	-0.1921	-0.0398	0.6646	-0.2091	-0.1737	-0.1298	-0.1944	0.0636	
MM002_2	-0.1777	-0.1203	0.682	-0.2315	-0.0963	-0.1547	-0.3085	0.0359	
MM002_3	-0.1275	-0.1902	0.7072	-0.1933	-0.1156	-0.1302	-0.2215	0.0453	
MM113_2	0.0589	-0.1065	0.0556	-0.1415	-0.0036	-0.0366	-0.083	0.1946	

Supplementary Table S9. Aminopeptidase gene copy number variation scores from MM patient samples in the FIMM dataset (*n* = 169). (15/15)

SampleID	*RNPEP*	*RNPEPL1*	*TPP1*	*TPP2*	*TRHDE*	*XPNPEP1*	*XPNPEP2*	*XPNPEP3*	Gene
MM003	0.529	-0.2642	-0.2773	-1.1437	0.0752	-0.1385	-0.0493	0.0366	
MM004	0.0077	-0.0862	0.0336	-0.1691	-0.0484	-0.012	-0.0287	0.087	
MM117_2	0.4331	-0.1525	0.0026	-1.1542	-0.08	-0.0784	-0.1448	0.2495	
MM124	0.5022	-0.0952	-0.0677	-0.1548	-0.0248	-0.0593	-0.055	0.1023	
MM129	1.4344	-0.1834	-0.4	-0.7304	-0.777	-1.206	1.0153	0.1456	
MM130	0.4316	-0.0683	0.0142	-0.1539	0.0185	-0.0384	-0.1468	0.109	
MM005	0.5373	-0.4598	0.0761	-0.4567	-0.1285	-0.2838	0.032	-0.1861	
MM135	-0.1217	-0.2206	0.0877	0.8774	-0.0355	-0.0999	-0.1679	0.1131	
MM137	0.2678	-0.1328	0.0846	-1.2188	-0.4668	-0.1446	-0.0817	0.1243	
MM006	0.9533	-0.0406	0.0466	-0.9449	-0.8002	0.035	-0.0645	0.15	
MM007	0.4829	-0.0198	-0.0113	-0.943	-0.0211	-0.0228	-0.018	-0.7874	
MM008	-0.0584	-0.0666	0.0012	-0.0878	0.01	-0.0133	-0.0715	0.1334	
MM009	-0.0519	-0.0821	0.2137	-0.0219	-0.0165	-0.0396	0.0567	-0.0497	
MM010	-0.0295	-0.0936	0.5112	-0.1193	-0.0694	-0.0711	-0.0648	-0.0008	
MM011	0.493	-0.4058	0.0832	-0.9619	0.0184	-0.0686	-0.8478	0.2018	
MM014_4	0.2682	-0.1755	-0.1872	-0.828	-0.1382	-0.1745	-0.1172	-0.1161	
MM014_1	0.2613	-0.078	-0.173	-0.6301	-0.1412	-0.0017	-0.158	-0.0852	
MM014_2	0.3611	-0.1372	-0.1318	-0.9918	-0.143	-0.1754	-0.2026	-0.029	
MM015	-0.0031	-0.0965	0.0318	-0.1718	-0.0283	-0.0201	-0.1282	0.2198	
MM017_1	0.464	-0.1347	-0.0685	-0.9949	-0.1213	-0.1296	-0.9724	-0.0419	
MM019	-0.1171	-0.1561	0.6978	-0.1712	-0.1082	-0.1085	-0.1437	0.1704	
MM022_1	0.1805	-0.0362	-0.045	-0.7024	-0.0005	-0.0255	-0.0301	-0.0131	
MM022_2	0.5948	0.0553	-0.0837	-0.9542	-0.0164	-0.0088	-0.1689	0.0682	
MM022_3	0.5601	0.1065	-0.0732	-0.821	-0.0129	-0.0174	-0.1866	0.1363	
MM023	-0.0947	-0.9155	0.4066	-0.1217	-0.0928	-0.1161	-0.139	-0.0448	
MM024	-0.1972	0.0132	0.436	-0.3895	-0.2891	-0.2199	0.4446	-0.6009	
MM025_1	0.0382	-0.0344	-0.0578	-0.1931	-0.1938	-0.3766	-0.1397	0.1451	
MM025_2	-0.041	-0.1498	-0.1485	-0.3501	-0.2743	-0.8641	-0.2354	0.4144	
MM017_2	0.3296	-0.1541	-0.0755	-0.8426	-0.1332	-0.1367	-0.9351	0.0317	

Supplementary Table S10. Summary of live cells, and CD138+CD38+ plasma cells present in the BM-MNC samples after 72h incubation in DMSO (control) (*n* = 15).

	Live cells		CD138+CD38+ cells	
Sample ID	cell count	Live/Singlet cells (%)	cell count	CD138+CD38+/Live cells (%)
MM007	9852	37.7	349	3.54
MM010	18525	59.65	125	0.68
MM012	17960	63.00	2093	11.65
MM021	6489	30.27	548	8.44
MM025_1	31431	73.29	110	0.35
MM037	18560	68.36	851	4.58
MM082	14337	46.75	257	1.80
MM087	11538	39.75	2431	21.07
MM106	24702	79.18	223	0.90
MM112	10419	61.49	70	0.68
MM124	5594	53.27	559	10.00
MM125	18542	53.72	1636	8.82
MM127	11467	57,93	2157	18.81
MM128_1	12843	49.79	6913	53.82
MM128_2	28045	50.44	554	1.97

Average number of cells in the control DMSO wells for the particular cell populations are indicated as cell counts. The fraction these cells make of all singlet cells, or of all live cells in the DMSO wells is indicated as a percentage.
BM-MNC: bone marrow mononuclear cell

Supplementary Table S11. Melflufen, melphalan, selinexor, bortezimob, and 4-HC EC50 values from 15 MM patient sample CD138+CD38+ plasma cells with sample disease stage indicated

	EC50 (nM)					
Sample ID	Melflufen	Melphalan	Selinexor	Bortezomib	4-HC	Disease stage
MM007	**0.001414**	**290.1**	NA	NA	NA	RRMM
MM010	**2.185**	**53.28**	**31.30**	NA	NA	RRMM
MM012	**0.2316**	1070	61.43	12.31	>1E+4	RRMM
MM021	**1.9e-21**	**237.1**	**32.25**	NA	NA	RRMM
MM025_1	**0.01096**	1638	**19.68**	NA	NA	RRMM
MM037	**0.000285**	**264.2**	**1.568**	**4.239**	NA	RRMM
MM082	**2.992**	1473	**41.00**	**1.628**	NA	RRMM
MM087	13.81	1004	**21.00**	**4.645**	3800	NDMM
MM106	**1.655**	1939	64.70	NA	NA	NDMM
MM112	**0.9372**	2667	**21.36**	**6.781**	NA	NDMM
MM124	**0.04291**	**556**	**39.30**	**1.227**	1900	RRMM
MM125	**0.8468**	3718	48.20	11.45	NA	NDMM
MM127	43.51	4000	46.70	NA	NA	NDMM
MM128_1	134.5	14781	**35.39**	24.18	>1E+4	NDMM
MM128_2	16.19	5920	NA	NA	NA	RRMM
Median	0.9372	1473	35.39	5.713	NA	

EC50: half maximal effective concentration; MM, multiple myeloma; NDMM: newly diagnosed multiple myeloma; RRMM: relapsed/refractory multiple myeloma; 4-HC: 4-hydroperoxycyclo-phosphamide; NA: not available

Supplementary Table S12. Mean log2(RPKM) values for the 39 aminopeptidase genes in melflufen high sensitivity (*n* = 5) and low sensitivity (*n* = 5) myeloma samples.

	High sensitivity samples (n=5)		Low sensitivity samples (n=5)			
Aminopeptidase	mean Log2 RPKM	standard deviation	mean Log2 RPKM	standard deviation	p-value	adjusted p-value
LAP3	5.206	0.635	5.605	0.710	0.296	1
ERAP2	4.985	0.496	4.990	1.170	0.531	1
METAP2	4.309	0.347	4.589	0.449	0.296	1
TPP2	4.568	1.232	4.131	0.563	1	1
ERAP1	3.850	0.625	4.188	0.238	0.676	1
DPP7	4.079	0.762	3.852	1.051	1	1
LTA4H	3.887	0.557	3.622	0.418	0.403	1
LNPEP	3.078	0.567	3.045	0.449	1	1
METAP1	2.522	0.668	2.634	0.416	1	1
XPNPEP1	2.293	0.349	2.381	0.534	0.835	1
DPP3	2.371	0.580	2.468	0.838	1	1
DPP8	2.328	0.473	2.158	0.274	0.531	1
NPEPPS	2.070	0.365	1.373	0.879	0.296	1
BLMH	2.080	1.267	2.046	0.555	0.403	1
RNPEP	1.778	0.640	1.466	1.372	0.676	1
PEPD	1.610	0.337	1.655	0.447	0.676	1
PGPEP1	1.615	0.831	1.831	0.590	0.676	1
JMJD7	1.659	0.564	1.441	0.275	0.531	1
DNPEP	1.296	0.296	1.138	0.518	0.835	1
DPP9	0.803	0.814	0.966	0.881	0.676	1
METAP1D	0.334	0.629	0.267	0.941	1	1
NPEPL1	-0.007	0.678	0.132	0.519	0.676	1
TPP1	0.190	0.934	-0.083	0.939	0.676	1
XPNPEP3	-0.117	0.485	-0.384	1.544	1	1
CTSH	0.256	2.145	-0.117	1.781	1	1
RNPEPL1	-0.720	0.457	-0.479	1.177	0.676	1
AOPEP	-0.652	0.460	-0.902	0.875	0.531	1
KDM8	-1.560	0.836	-1.585	0.718	1	1
DPP4	-3.367	2.173	-4.522	1.906	0.210	1
LVRN	-4.358	1.461	-3.939	0.917	1	1
ENPEP	-4.768	3.525	-4.295	0.554	0.403	1
MMP14	-2.994	2.009	-4.969	1.461	0.095	1
ANPEP	-3.743	1.996	-6.890	2.395	0.060	1
NAALADL1	-3.969	2.202	-4.549	2.919	0.835	1
AMZ1	-4.908	1.638	-5.405	1.937	0.676	1
CTSV	-7.683	2.920	-5.118	2.281	0.144	1
XPNPEP2	-7.876	1.809	-7.530	3.347	1	1
TRHDE	-8.298	2.094	-10.327	2.494	0.210	1
F11	-9.087	1.959	-7.910	3.135	0.676	1
